# Research on visual search behaviors of basketball players at different levels of sports expertise

**DOI:** 10.1038/s41598-023-28754-2

**Published:** 2023-01-25

**Authors:** Peng Jin, Zhigang Ge, Tieming Fan

**Affiliations:** 1 Department of Physical Education, Southeast University, Nanjing, China; 2 No. 9 Middle School, Nanjing, China

**Keywords:** Psychology, Health occupations

## Abstract

This study aims to examine visual search strategies of skilled basketball players in an anticipation task. This study selected 48 experienced and inexperienced basketball players. The participants were grouped into novice and expert groups based on their experience. The participants were asked to look at series of pictures of offensive patterns of play in a basketball game from a third person perspective and chose one of the three options: passing, shooting and break through. This study measured and recorded the response time, key pressing results, and eye movements. Eye movement data were recorded using the Tobii X-3 120 eye tracker. The results showed that, the expert group demonstrated superior anticipation performance, and were more accuracy. In addition, the results showed that participants in the expert group and faster predicted the offensive way faster than the novice group. The results also showed that experienced basketball players employed a simple and efficient visual search strategy including greater fixation counts and longer fixation duration on more informative areas than the novice group. Furthermore, the visualization metrics showed that the expert group had a more concise fixation trajectory and focused mainly on key information area. Generally, expert players exhibited a more efficient and effective visual search strategy demonstrating better performance on anticipation tasks.

## Introduction

The ability to anticipate and make accurate decisions on time is fundamental to high performance in sports, particularly team ball games and racket sports^1,2^. This claim is supported by visual search studies that use eye registration techniques to examine where expert players direct their line of sight^3,4^. Visual search is one of the key factors behind anticipation. It is also one of the common methods used to gain insight into how athletes make decisions in complex and time-constrained situations.

Visual search, the process of finding a target from a set of irrelevant distractor items^5,6^, is an important way for people to obtain and process exterior information to interact with the environment effectively^7^. Regarding motor behavior, visual search ability is a key factor in determining the effectiveness of some professional work, including drivers^8^, beach lifeguards^9^, and many military tasks^10^. Visual search is also important in individual sports like judo^11^, cycling^12^, tennis^13^, and team sports like soccer^14^, volleyball^15^, baseball^16^, and handball^6^. Visual search can reveal how athletes process visual information. In addition, visual search can also reflect the cognitive abilities of athletes and aid in decision-making and judgment response which affect performance. Therefore, exploring the visual search characteristics of athletes helps select elite athletes and determine the most effective training that can improve special perceptual skills.

Sports scientists have devoted their efforts to investigating the visual search characteristics of athletes over the past years. Many studies have found differences in visual search strategies between athletes with different expertise in open sports such as soccer^4^, volleyball^17,18^, and futsal^19^. Few studies have indicated that experienced athletes demonstrate fewer but longer fixation to more important areas than novice athletes^20–22^. On the contrary, some authors have reported that experienced athletes have more fixations and shorter durations than novice athletes^23,24^. Several studies have demonstrated that visual search strategies are influenced by the type of response^25^, the number of players involved^26^, methods applied^27^, and task complexity^28^. These divergent results illustrate that experienced athletes use a different visual search strategy depending on various conditions. Many studies have focused on visual attention as a whole rather than the various parts of visual attention and their role and status in problem discovery. As a result, it is impossible to know what visual attention characteristics benefit experienced players. Therefore, this study aims to investigate the three independent areas of interest (AOI) that constitute the whole offensive situation and their corresponding eye movement characteristics.

Basketball requires players to have a strong ability to process the visual information of fast movement to make judgments and decisions within a short time^29^. During a basketball match, players must analyze a lot of visual information, including factors such as teammates, opponents, and offensive or defensive game patterns^30^. The visual search ability of basketball players directly affects the processing quality and speed, this means that effective visual search strategies help players have better match performances. The development of "small ball tactics" in basketball has gradually become mainstream^31^. The game has the characteristics of "strong confrontation"^32^, "fast rhythm"^33^, and "multi transformation"^34^, which requires athletes to have higher visual search abilities. Therefore, exploring the visual search strategy of experienced basketball players can help athletes improve their performance. In addition, it can help coaches and scientists understand how expert players make decisions and process information^35^.

Most visual search studies in basketball have been limited to closed skills like free throws^36^ or jump shots^37^. As a result, many aspects of expert basketball players’ gaze behavior in open-play are still unknown. This study extended on the work by Maarseveen M V et al.^38^, which further analyze the visual search behaviors and decision-making of offensive ball handlers. This study used eye-movement registration techniques and an anticipation task, to compare the differences in visual search strategy between experts and novice athletes. The results reported in this study included key-press response time, response accuracy and gaze behavior. Visualization was added to make the results more intuitive. Participants were required to predict the offensive choice of the ball carrier in attack game scenarios (passing to the teammate, shooting at the basket or driving toward the basket). This study hypothesized that experience basketball players would exhibit a more efficient and effective visual search strategy than the novice group. It was assumed that experienced players have longer fixation duration and more fixation counts on critical AOI, more concise fixation trajectory and focus mainly on key information. In addition, it was assumed that experienced athletes could fixate on areas for longer periods and fewer periods on irrelevant areas. As a result, experienced athletes can make sports decisions with shorter response time and higher response accuracy.

## Methods

### Participants

The sample size was estimated using G*Power3.1.9 software. The sample size considerer the effect size of 0.80, an alpha level of 0.05, the power of 0.80, and two tails. In total, 48 male basketball players were recruited. The participants were divided into an expert group (n = 24, 12 guards and 12 forwards) and a novice group (n = 24, 11 guards and 13 forwards) according to their basketball experience and skills. The expert group comprised eight professional players from China Basketball Association (CBA) teams and 16 college players from China University Basketball Association (CUBA) teams. The average age of the participants was 20.36 years (SD = 2.72), On the other hand, the average years of training for the participants were 9.46 years (SD = 2.68). In addition, all the players were national first-level athletes. The novice group comprised 24 physical education undergraduate students from the Nanjing Sport Institute. The average age of the participants in the novice group was 21.65 years (SD = 2.19), The novice group had an average of 2.46 years of training experience (SD = 2.97). All participants reported normal or corrected-to-normal levels of visual function and were right-handed. In addition, the participants gave their written informed consent following the Declaration of Helsinki. The study was approved by the ethics committee of the Shanghai University of sport (NO.2015003SUS), the participants who completed this study were compensated for their time.

### Apparatus

This study used the Tobii Pro X3-120 (Tobii Technology Danderyd, Sweden) eye tracker to measure the gaze behavior during the presentation of stimuli at a constant frame rate of 120 Hz per second utilizing the “corneal reaction techniques”. The data collected using the Tobii Pro X3-120 eye tracker was recorded using a high-performance Dell notebook computer. The numeric keys are also used to judge the response of keys. The study also used one Dell LCD monitor(23 inches, resolution of 1920 × 1080 pixels, refresh rate 120 Hz) to present experimental materials. The eye tracker device was placed under the Dell external monitor and 55-65 cm from the subjects to ensure the stability and high accuracy of eye tracking. Measures of gaze behaviors were calculated using the Tobii studio version 3.3.8 software.

### Test stimuli

The test stimuli were based on Adobe Premiere Pro CC software from NBA games. The stimuli included a series of pictures of offensive patterns of play in a basketball game from a third person perspective. Highly experienced basketball coaches assessed the images before being used in this study. AdobePhotoShopCS6 software processed the selected pictures to ensure consistent brightness and saturation. In total, 21 pictures, including six practice pictures and 15 test pictures were included in this study. On the other hand, the clips composed of three types of offensive scenario diagrams; passing, breakthrough and shooting. All the visuals were made into JPG files and imported into Tobii Pro Lab software, which was also used to program and present the experiment. The participants were required to make judgments on the pictures and press the keys to complete the task.

### AOI

Areas of Interest (AOIs) enable numerical and statistical analysis based on regions or objects of interest in stimuli. Researchers can divide and define AOI based on their experimental hypotheses and research objectives. The Tobii Pro Lab software allows the researcher to divide the AOI before or after the experiment. Therefore, and the size, shape and position of the AOI can be controlled according to the needs of the researcher. This study identified three AIOs based on the literature and the research goals (see Supplementary Figure S1). The three AOIs were determined by the coaches based on the evaluation of threat area during attack: K-AOI = key areas of interest(ball hander, ball hander defender, screener/roller, screener/roller defender, best choice teammate), R-AOI = related areas of interest(functional space, i.e., areas of free space in front of the defensive player), I-AOI = irrelevant areas of interest (the remaining space undefined)^3,39^.

### Procedure

The participants sat on a chair facing the monitor where the test film was presented. After adjusting the sitting position, the participants were asked to tap their right-hand index finger, middle finger, and ring finger on the keys numbered “1,2,3” on the notebook. The participants sat at a distance of 60 cm from the external monitor and a standard 9-point calibration procedure was performed. After calibration, the participants were show the instructions about the task (Instruction: Several pictures of the basketball game will appear on the screen. Each clip will present for a 5000 ms. Please observe carefully and make a keypress selection within 5000 ms. "1" represents pass, "2" represents shoot, and "3" represents breakthrough). After 10 s, the participants received six practice trials to ensure that they were familiar with the experiment procedure (all subjects performed more than 60 key presses to get familiar with the key position). Finally, if the participants had no questions, there was a reminder indicating the beginning of the experiment, and the experiment began after three seconds.

**Figure 1:**
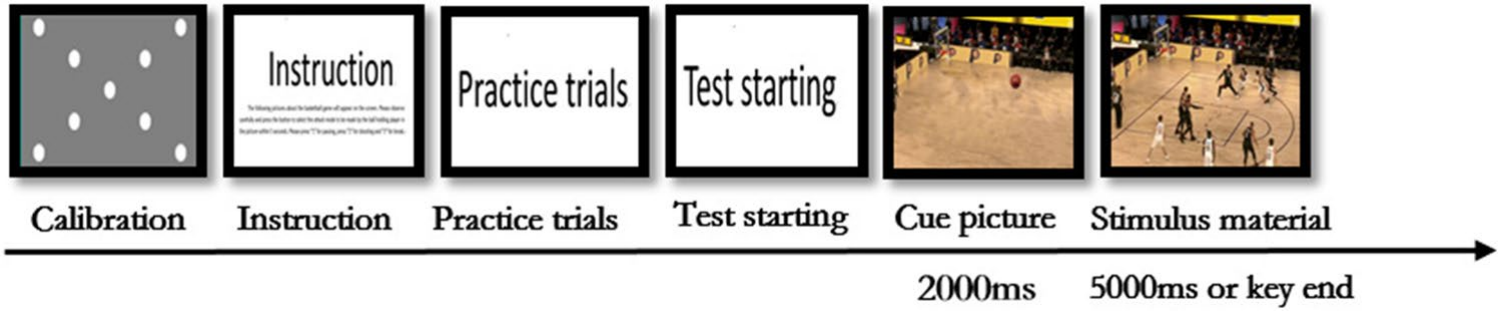
Illustration of the sequential phases included in a visual search task.

The study was conducted in trials, and each trial started with a cue picture displayed for 2000 ms. The only image on the screen was the position information of the basketball. The participants were required to only focus on the position of the basketball. The cue was also to inform the participants that the test starts immediately. After 2000 ms, the game scene film was displayed for 5000 ms, the participants were asked to respond as quickly and as accurately as possible to the filmed stimuli. During the presentation, participants were required to press the keystrokes to make a judgement. The next cue picture appeared if the participants failed to press any key within the given timeframe. The correct answers were assessed when all five coaches reached a consensus. The Tobii Pro X3-120 eye-tracker used in this experiment collected date on eye movement and recorded the reaction time and key pressing results of the participants. Participants completed 15 trials, and each test session was completed in approximately 10 min. (see Fig. 1).

### Ethics statement

The study was performed in accordance with the recommendations of the “ethics committee of the Shanghai University of sport” with written informed consent from all participants. The participants gave their written informed consent in accordance with the Declaration of Helsinki. The protocol was approved by the “ethics committee of the Shanghai University of sport (No. 2015003SUS)”.

## Dependent variables and data analysis

### Anticipation test

*Response time (RT)*: the time taken for the participants to press the keystrokes to make a judgment. Response time was measured from when the image was displayed to when the key was pressed..

*Response accuracy (RA)*: the participants was measured using the number of accurately identified images.

### Eye tracking data

*Fixation:* A fixation was defined as a condition in which the eye remained stationary within a tolerance of movements up to 3° for a period greater than 100ms^40^.

*Fixation duration*: The total duration of the fixations inside this area of interest during an interval (Tobii Prolab_UserManual).

*Fixation count*: The number of fixations occurring in this area of interest during an interval (Tobii Prolab_UserManual).

### Visualization

*Gaze plot*: A gaze plot is a visualization tool for showing the location, order and time spent at locations on the stimulus. This study used a gaze plot to reveal the time sequence of looking or where and when the participants looked.

*Heat map*: A visualization tool for showing how looking is distributed over the stimulus. This is a visualization that can effectively reveal the focus of visual attention for dozens or even hundreds of participants at a time.

### Data analysis

Data were analyzed using Tobii Pro Lab (version1.21.21571). The eye movement data collected in this experiment were sorted and analyzed using the Tobii Pro Lab software. The reaction time and key selection are automatically recorded by the Participant Events in the Analysis Module. The collected data was then exported to SPSS 23.0 as an EXCEL sheet. Statistical data analyses were done on SPSS 23.0, and the sampling rate of 85% was decided as the standard in this experiment. Each test film was divided into three AOIs for statistical analysis. The gaze plot and heat map are presented in the visualization module. This study performed t-tests to analyze response accuracy, response time, percentage of fixation duration in AOI, percentage of fixation counts in AOI according to expertise (experts versus novices). The standard level of significance was set as *p* < 0.05.

## Result

### Anticipation test

#### Response time (RT)

**Figure 2:**
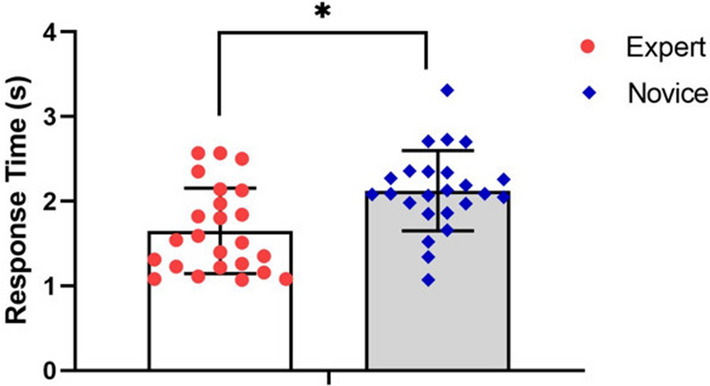
Comparison of the response time between groups.

Figure 2 presents the means and standard deviations of the response time between experts and novices, means and standard deviations calculated as the average overall clips for each individual, as well as the grand average for the group. The K–S test was used, which presented normal distribution. On this basis, the independent sample T-test was performed on response time of the two groups. As shown in Fig. 2, the experts group had a significantly faster response time than the novice group (t =  − 3.35, *p* < 0.05; d = 0.96).

#### Response accuracy (RA)

**Figure 3:**
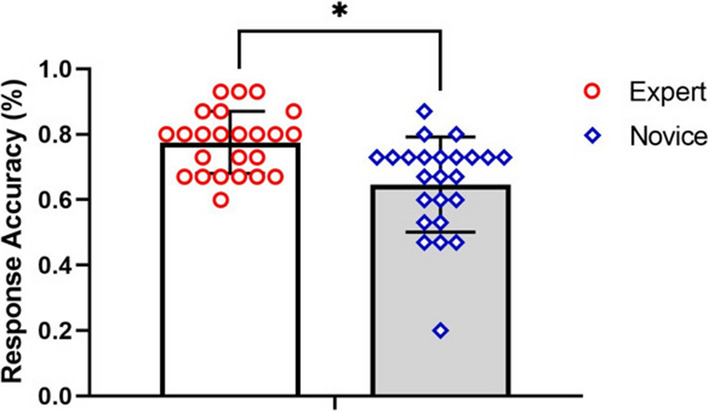
Comparison of the response accuracy between groups.

The difference in response accuracy between the two groups is shown in Fig. 3. The difference in RA is presented as an average and standard deviation. This study used the K-S test, which presented normal distribution, and an independent sample t-test which compared the two groups. The results in Fig. 3 show that the response accuracy of expert players was greater than that of novice players (t = 3.63, *p* < 0.05; d = 0.89). This result indicates that expert players can make better judgments than novice payers in visual search tasks.

### Eye tracking data

#### Percentage of fixation duration in AOI

**Figure 4:**
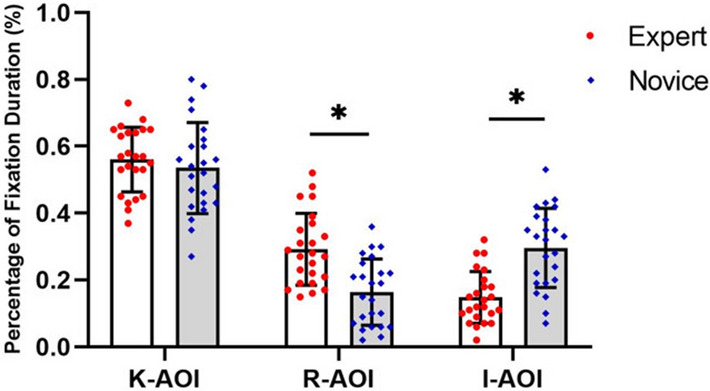
Comparison of the percentage of fixation duration in AOIs between groups.

The independent t-test results show that expert players and novice players spent more time fixated on K-AOI (M = 0.56 ± 0.10 for the expert group vs. M = 0.53 ± 0.14 for the novice group). However, there were no differences between the two groups (t = 0.75, *p*˃0.05; d = 0.25). The study found that expert players demonstrated a greater percentage of fixation duration in R-AOI than novice players (M = 0.29 ± 0.11 vs. 0.17 ± 0.09, t = 4.30, *p* < 0.05; d = 1.19). Also, expert players spent a smaller percentage of fixation duration in I-AOI than novice players (M = 0.15 ± 0.07 vs. 0.30 ± 0.12, t =  − 5.13, *p* < 0.05; d = 1.53) (see Fig. 4).

#### Percentage of fixation counts in AOI

**Figure 5:**
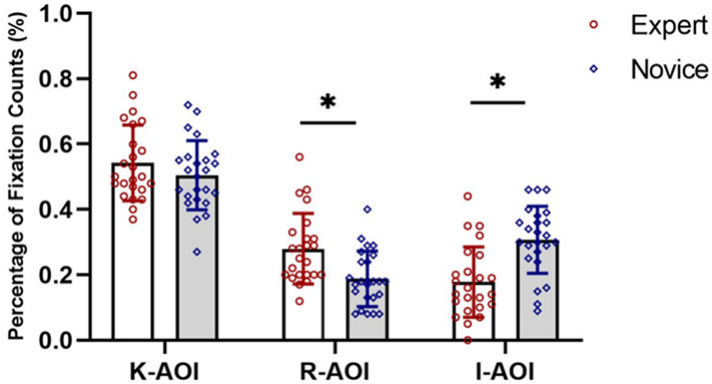
Comparison of the percentage of fixation counts in AOIs between groups.

The results show that although the expert and novice groups spent more percentage of fixation counts in K-AOI (M = 0.54 ± 0.12 vs. M = 0.50 ± 0.10), there was no difference between the two groups in terms of the percentage of fixation counts in K-AOI (t =  − 1.17, *p* > 0.05; d = 0.36). The results also show that the expert group had a greater percentage of fixation counts in R-AOI than novice players (M = 0.28 ± 0.11 vs. M = 0.19 ± 0.08, t =  − 1.17, *p* < 0.05; d = 0.94). On the contrary, the novice group had a higher percentage of fixation counts in I-AOI than the expert group (M = 0.18 ± 0.11 vs. 0.31 ± 0.10, t =  − 4.25, *p* < 0.05; d = 1.24) (see Fig. 5).

### Visualization

#### Gaze plot

**Figure 6:**
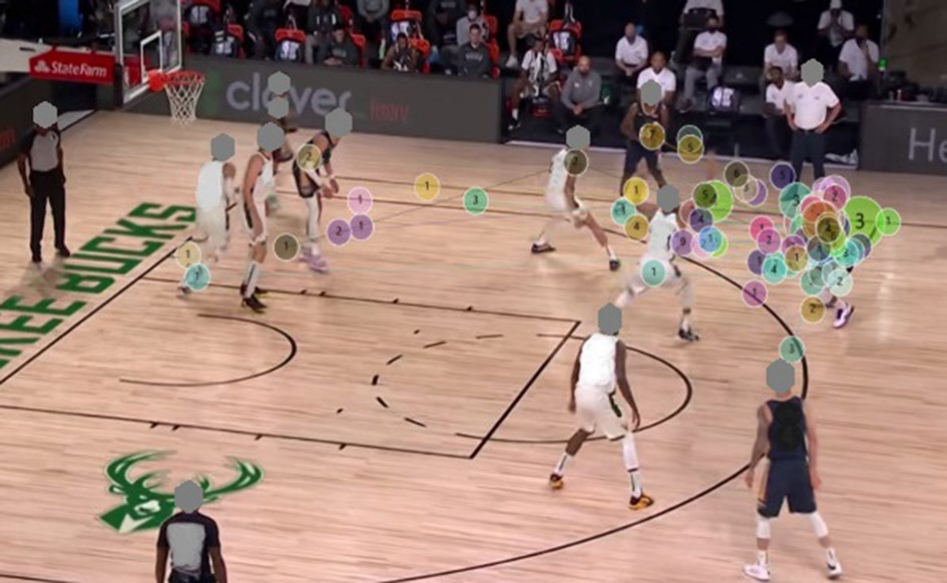
Aggregated Gaze Plot for experts.

**Figure 7:**
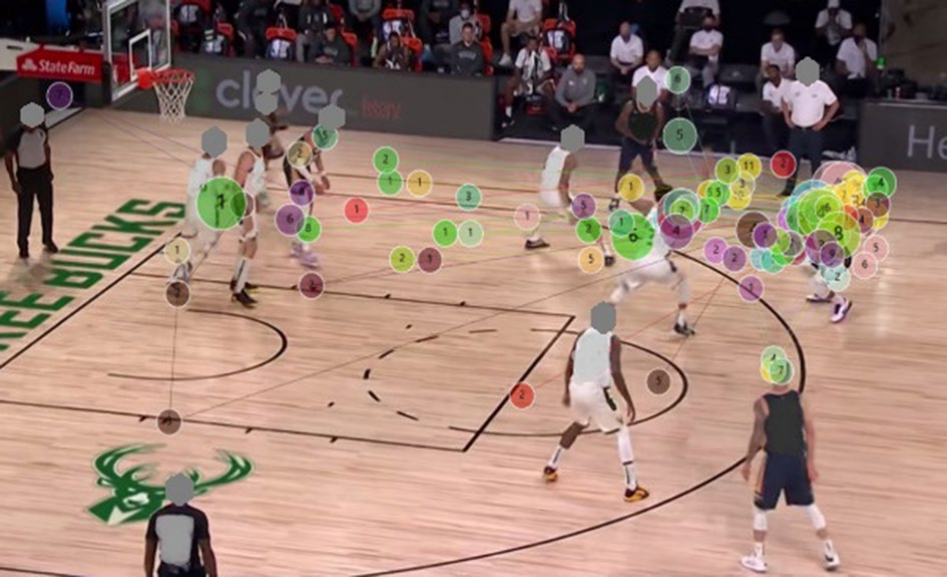
Aggregated Gaze Plot for novices.

**Figure 8:**
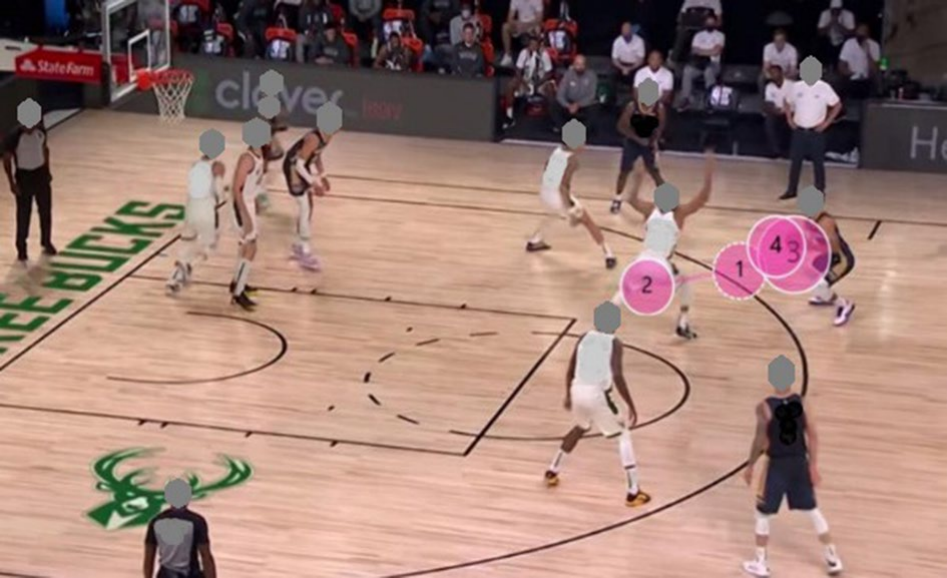
Example of Gaze Plot for experts.

**Figure 9:**
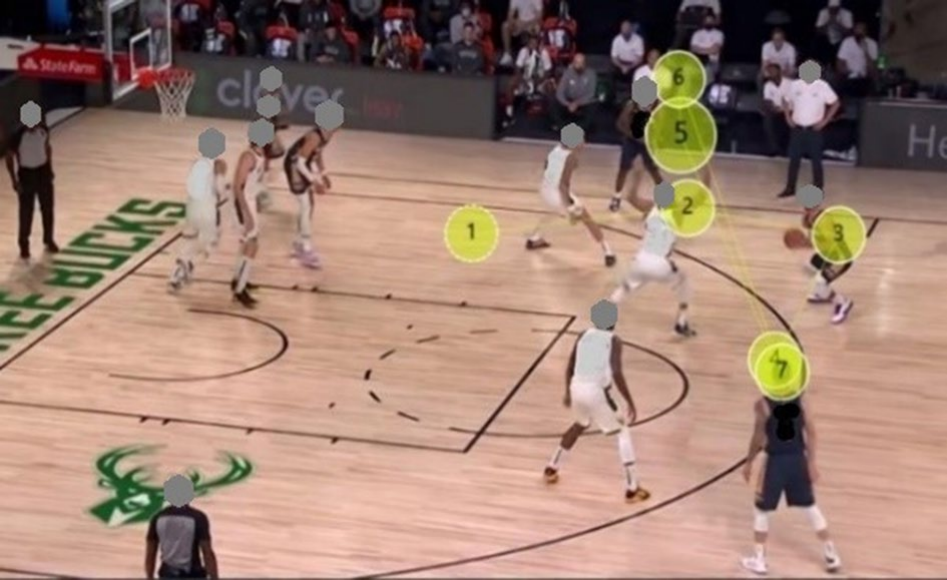
Example of Gaze Plot for novices.

The gaze plot illustrates that the gaze trajectories of the two groups are different. The expert group had fewer fixation counts, and the fixation (dot) was concentrated in the K-AOI and R-AOI. On the contrary, the fixation counts of the novice group were scattered and irregular (see Figs. 6, 7, 8, and 9).

#### Heat map

**Figure 10:**
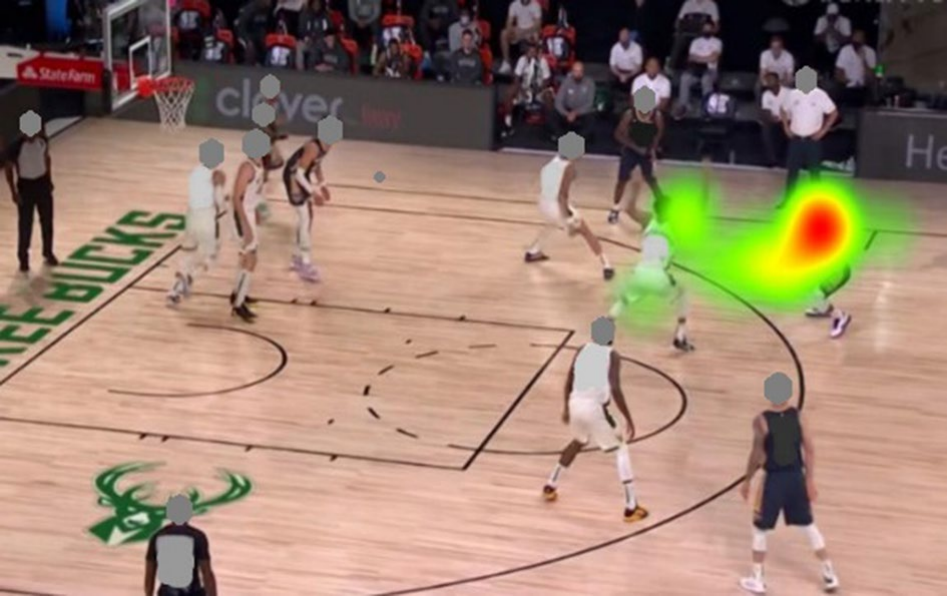
Heat map for experts.

**Figure 11:**
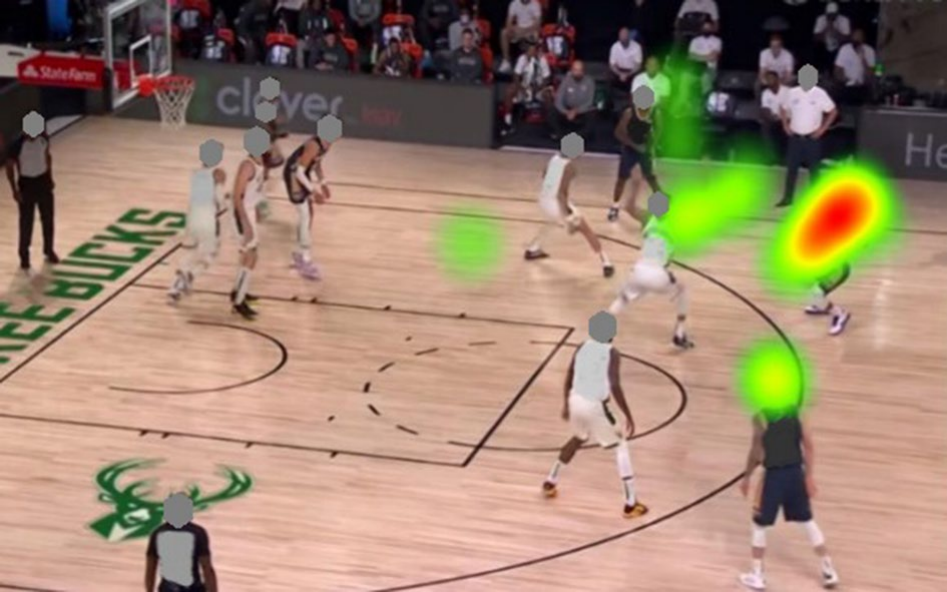
Heat map for novices.

The heat map shows that the attention of the expert groups was more focused than the attention of the novice group. The heat map also shows that the expert group pays more attention to the K-AOI and R-AOI, while the novice group not only pays attention to the K-AOI but also allocate more attention resources to the I-AOI (see Figs. 10 and 11).

## Discussion

The general aim of the current study was to analyze the difference in visual search strategies between expert and novice basketball players using film-based offensive scenarios of a basketball game. The results supported the hypothesis: expert basketball players have higher response accuracy and shorter response time. In addition, the results of the eye tracking date and visualization also showed that expert athletes have more advantages in judging and processing key information areas and more reasonable fixation distribution than novice players.

As predicted, the expert group was faster in response time and more accurate in predicting offensive ways than the novice group. This result is consistent with the previous research involving players with different skill levels^24,41,42^. This is likely because expert players have accumulated domain-specific knowledge and structural information through a lot of time spent in competition and training. Therefore, when presented with similar competitive scene, experts develop strong sense of identity and use fewer cognitive steps to make decisions resulting in a shorter response time, this concept was also supported by Dittrich’s research^43^. In contrast, less-skilled players cannot successfully anticipate because they lack episodic and task-related expertise. As a result, novice players took a lot of time to respond and had a low response accuracy. However, this study is inconsistent with previous findings, especially studies relating to research in volleyball and French boxing^44,45^. They failed to find a significant difference in response time between different groups, which could be attributed to the specific demands and sport type. In addition, analysis of the gaze behaviors can better explain the anticipation process when expert players respond in a short time.

Unlike previous researchers, who investigated the differences in gaze behaviors between different skilled-level players, most studies in this research field have reported that the expert groups employ more efficient strategies that involved more number of fixations and shorter fixation duration than their novice counterparts^14,17,24,26^. This research has illustrated that expert players exhibit superior performance on the film-based test. However, several studies in this area adopts a holistic research approach where the experimental materials are considered as a whole and not divided into regions. There needs to be more differentiated research on the role and status of the distinct region.

In the study, we were mainly interested in examining the difference between two groups (expert and novice) of basketball players on percentage of fixation duration and Percentage of fixation counts on AOIs. The results showed that the expert and novice groups had a 50% fixation duration on the K-AOI. This result illustrates that experienced and inexperienced players realized that the K-AOI is the essential location to supply the critical information to understand the scenario better. This result is consistent with previous studies that reported that players prefer to focus on the players controlling the ball and the defenders because these factors are the most important elements for the players to extract effective information^46^. This visual feature helps experienced athletes improve their visual processing efficiency and respond correctly.

This study found skilled-based differences in the percentage of fixation duration and fixation counts on R-AOI and I-AOI. The study found that experienced players prefer spending a higher percentage of fixation duration and percentage of fixation counts on R-AOI and a lower percentage on I-AOI than the novice groups. In addition, it has been established that experienced players make more fixations of longer durations on relevant AOIs. The work by other studies also support our results. Researchers assessed expertise in chess and found that the experienced chess players pay more attention to the middle of the board than their chess pieces. This result suggests that experts are better at selectively allocating their attention and ignoring irrelevant areas^47,48^. On the other hand, experienced basketball players utilize their experience and knowledge to direct their attention to the R-AOI because those locations contain the functional space between the ball hander, the corresponding teammate, and their defenders to analyze their offensive choices. On the contrary, novice players pay attention to all the visual stimuli on the field due to the possibility that lack of experience. As a result, novice players have invested more attention resources in I-AOI.

These results are consistent with the existing evidence that the players with higher percentage of correct anticipation distribute more attention towards the functional place, however, the players with lower percentage of correct anticipation prefer to look more on the space outside the players^38^. Similar results have also been reported in other team ball sports such as soccer and volleyball^46,49^. Nonetheless, these results support the information reduction hypothesis: experts should exhibit longer fixation duration and more fixation counts on task-relevant areas and shorter fixation durations and fewer fixation counts on task-irrelevant areas^50^. In addition, these findings support the guide search theory which states that experienced participants will systematically move their focus from one AOI to another, guided by experience or previous knowledge^51^. However, some studies have found an opposite results pattern. These studies have reported that experienced players made fewer fixations of shorter durations on the relevant region^52,53^. These results are supported by the long-term working memory theory^54^. Experienced players encode and retrieve information more rapidly from long-term working memory, contributing to their shorter fixation durations than novices. This is because experts use parafoveal regions to extract information from many visual cues^11,55^.

Heat maps create a more user-friendly pattern for visualizing eye-tracking metrics for multiple subjects. Results show that K-AOI received the same attention (50%) from all subjects. As a result, it can be concluded that K-AOI is the most important decision area. However, the biggest difference in attention allocation between the experts and the novices is the attention allocation on R-AOIs and I-AOIs. The expert players invest more attention to the R-AOIs, which may be responsible for the "fast and accurate" anticipation tasks. In contrast, the novice players pay more attention to the I-AOIs, indicating that the novice group focused on more scattered areas and could not obtain the most critical information in a short time.

Gaze plot can comprehensively and intuitively illustrate the spatio-temporal characteristics of the visual search of participants, and is useful in showing a user’s sequence of fixation. The gaze plot resluts show that the fixation of the expert players is mostly concentrated in the K-AOI and R-AOI, and the fixation trajectory is simple and clear. Therefore, researchers have concluded that expert players know where the critical and relevant cues for proper decision-making are in the game scenario. In contrast, novice players fixate more on the I-AOI and show complex fixation points, irregular and more looking back trajectories. The fixation strategy of novice athletes reduces the efficiency of visual information processing and wastes limited attention resources. This strategy has been attributed to why novice players have slower response time and lower accuracy when performing decision-making task^38^.

There were several limitations to the present study. The main shortcoming was that the stimulating materials could not be presented from a first-person perspective, and future research should take stimulating pictures from the perspective of players. Second, this study utilized only offensive situations, yet a real match situation involves not only offensive situations but also defensive situations. Therefore, this study encourages future studies to use defensive situations to understand visual search strategies better. Third, video-based tests were not used in our study. Dynamic stimulation materials are more in line with the reality of the game. As a result, this study encourages future studies to use dynamic contexts to improve the ecological validity of the experiment. Additionally, this study did not take into account the relationship between observable cues and decision-making. Therefore, future researchers should try to demonstrate this relationship. Meanwhile, further investigation should employ a broader range of metrics to enrich our understanding about characteristics of visual search strategies among expert players. Finally, the present study is not a Randomized Controlled trial, it is impossible for our paradigm to organize participants randomly, it is always possible that highly practiced basketball players had better visual search strategies to start with. Therefore, a longitudinal design is necessary to observe the process of the change in visual search abilities longitudinally in basketball players as time goes on in the future study.

In summary, the results of the present study indicate that experienced basketball players exhibit a more efficient and effective visual search strategy than novice players. They employed a higher fixation duration and greater fixation counts on more informative areas when looking at the game-situation pictures. These visual search characteristics illustrate that expert basketball players demonstrate a faster response time and greater response accuracy in a decision-making test.

## Supplementary Information

Supplementary Figure S1.

Supplementary Information 2.

Supplementary Information 3.

Supplementary Information 4.

## Data Availability

The datasets generated and analyzed during in this study are available from the corresponding author upon reasonable request.
