# Supplementary figures and images for: Research on visual search behaviors of basketball players at different levels of sports expertise
Source: Sci Rep. 2023 Jan 25;13:1406. doi: 10.1038/s41598-023-28754-2 (PMC9876905; doi:10.1038/s41598-023-28754-2)

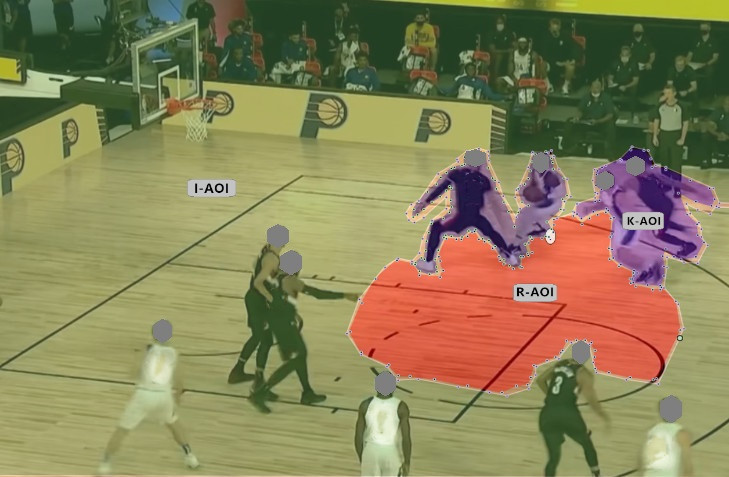

Supplement: Supplementary file 1 — Supplementary Figure S1. [file 41598_2023_28754_MOESM1_ESM.jpg]

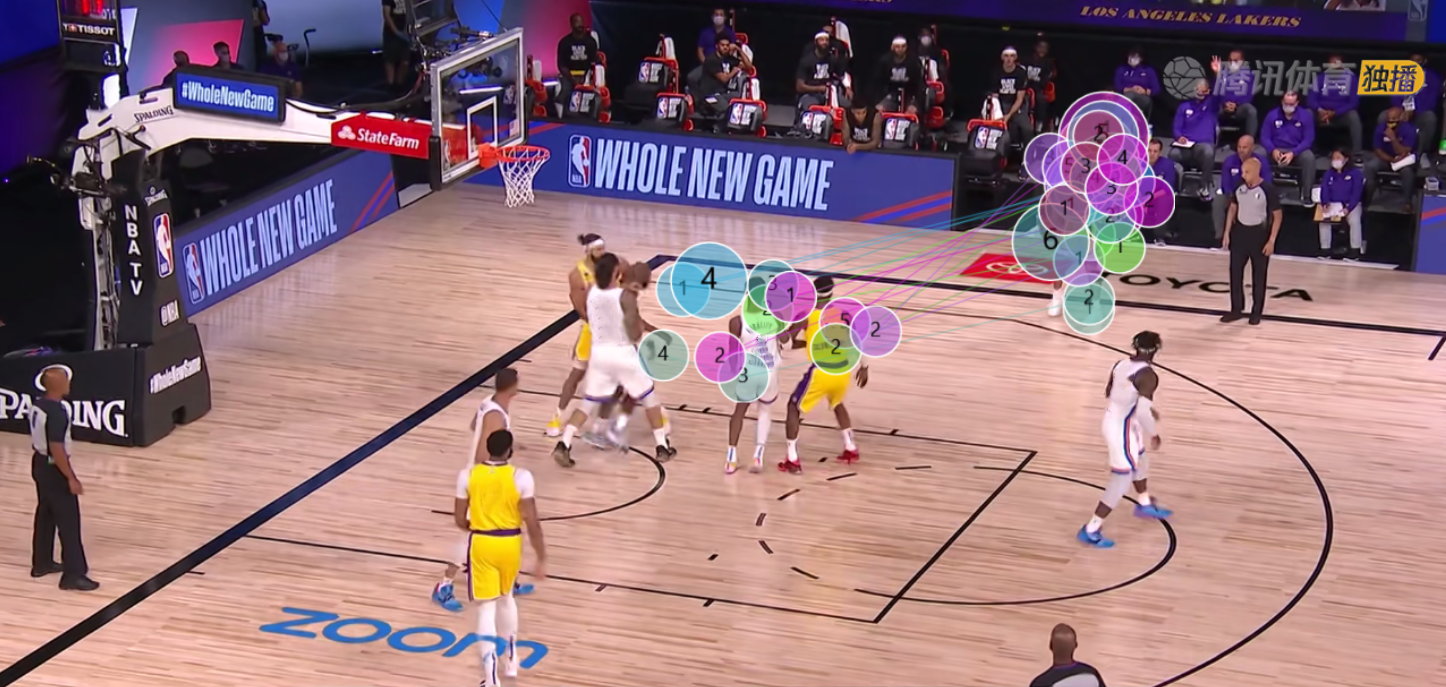

Supplement: Supplementary file 2 — Supplementary Information 2. [file 41598_2023_28754_MOESM2_ESM.zip › Gaze Plot/E10.png]

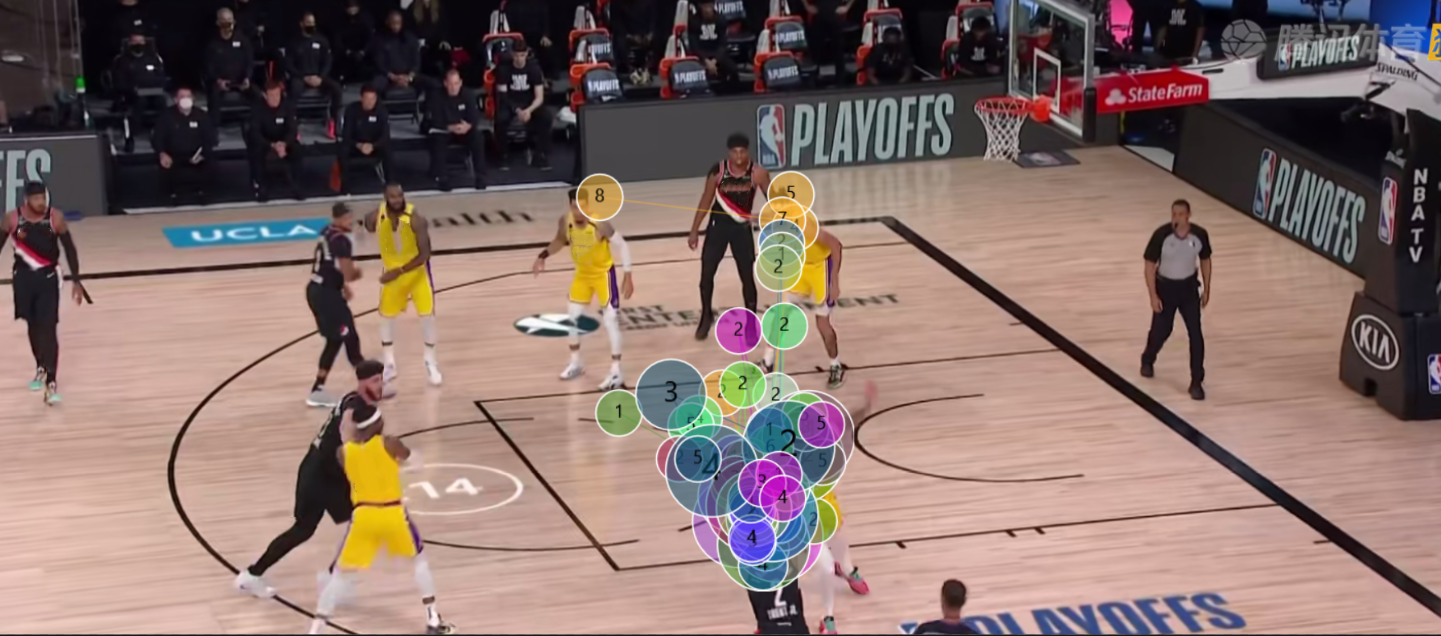

Supplement: Supplementary file 2 — Supplementary Information 2. [file 41598_2023_28754_MOESM2_ESM.zip › Gaze Plot/E11.png]

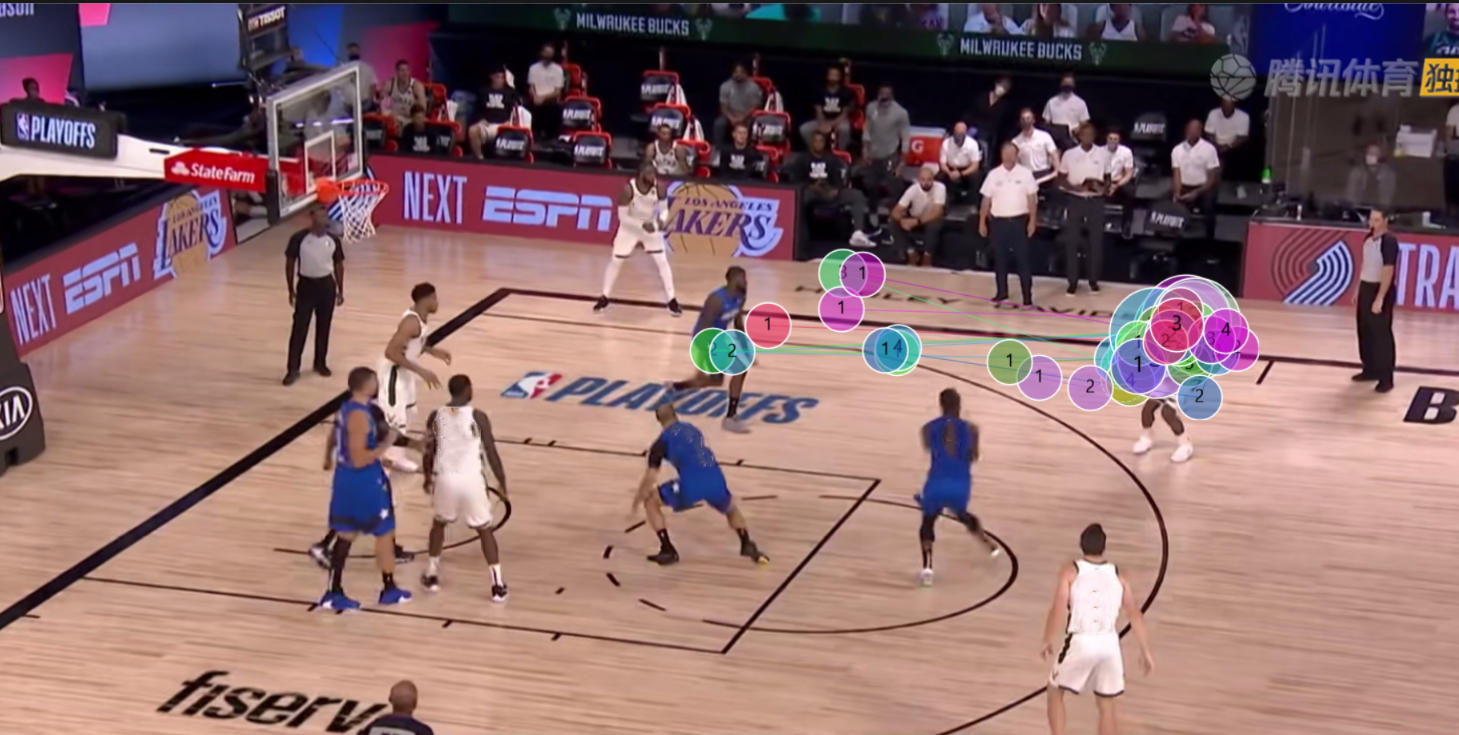

Supplement: Supplementary file 2 — Supplementary Information 2. [file 41598_2023_28754_MOESM2_ESM.zip › Gaze Plot/E12.png]

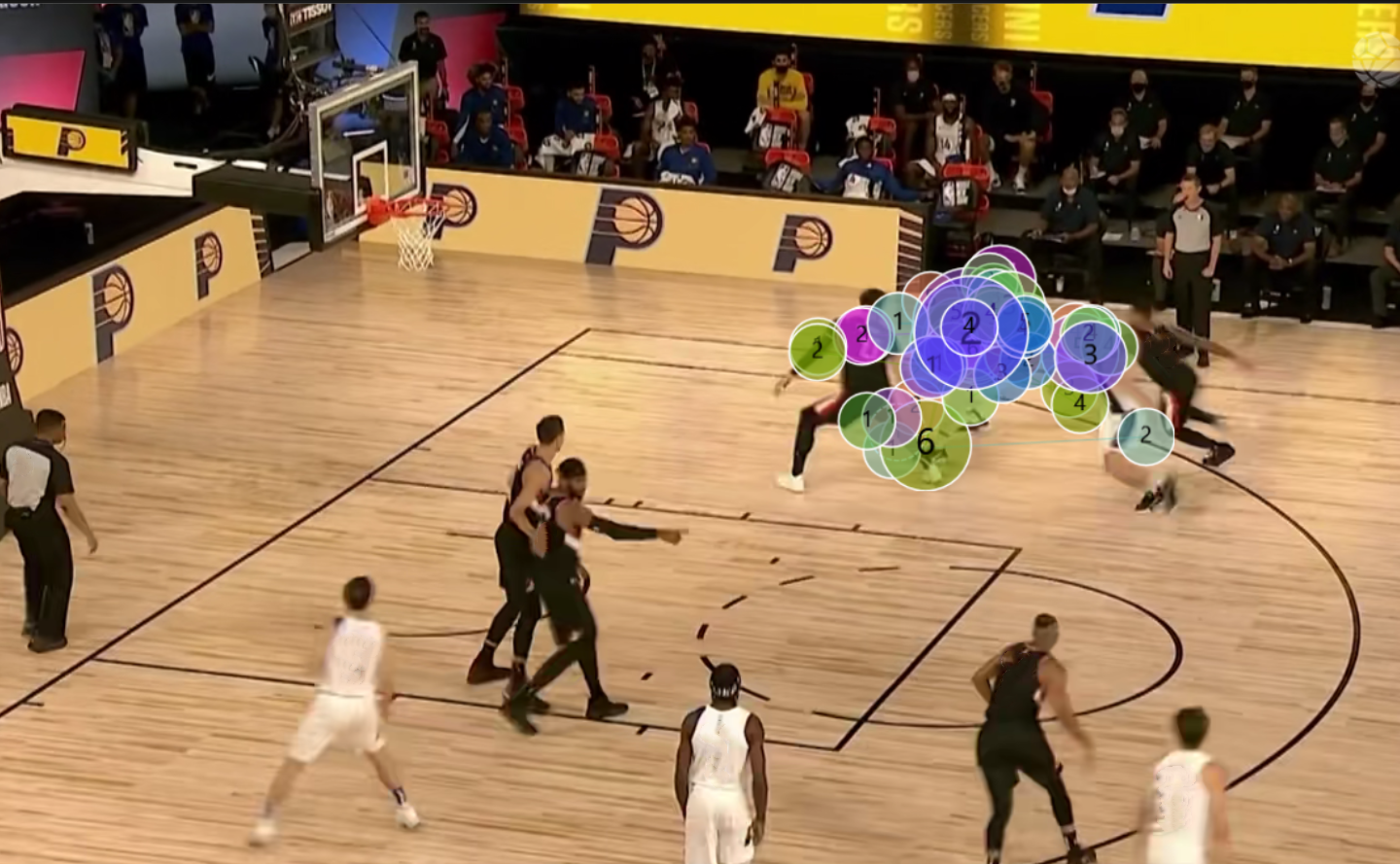

Supplement: Supplementary file 2 — Supplementary Information 2. [file 41598_2023_28754_MOESM2_ESM.zip › Gaze Plot/E13.png]

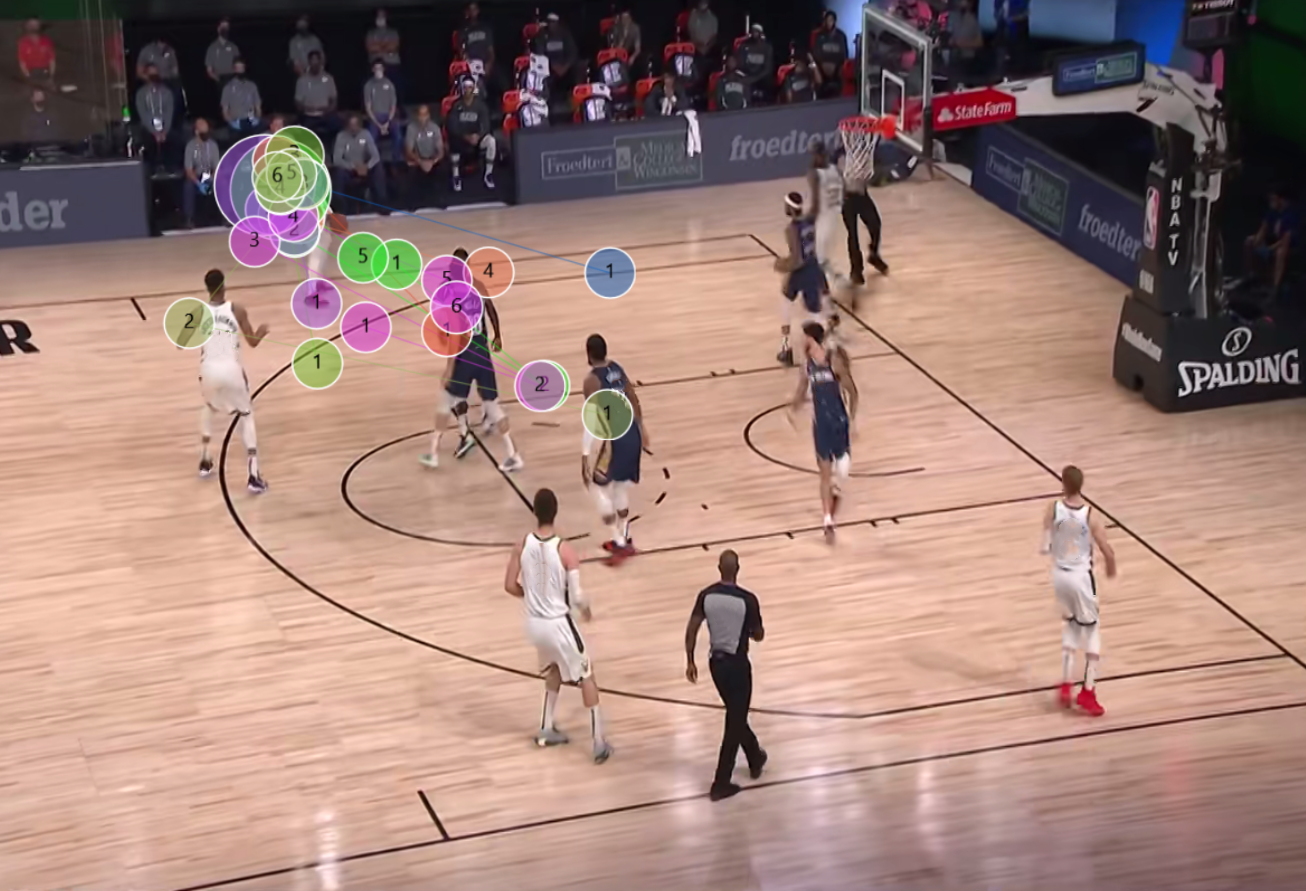

Supplement: Supplementary file 2 — Supplementary Information 2. [file 41598_2023_28754_MOESM2_ESM.zip › Gaze Plot/E14.png]

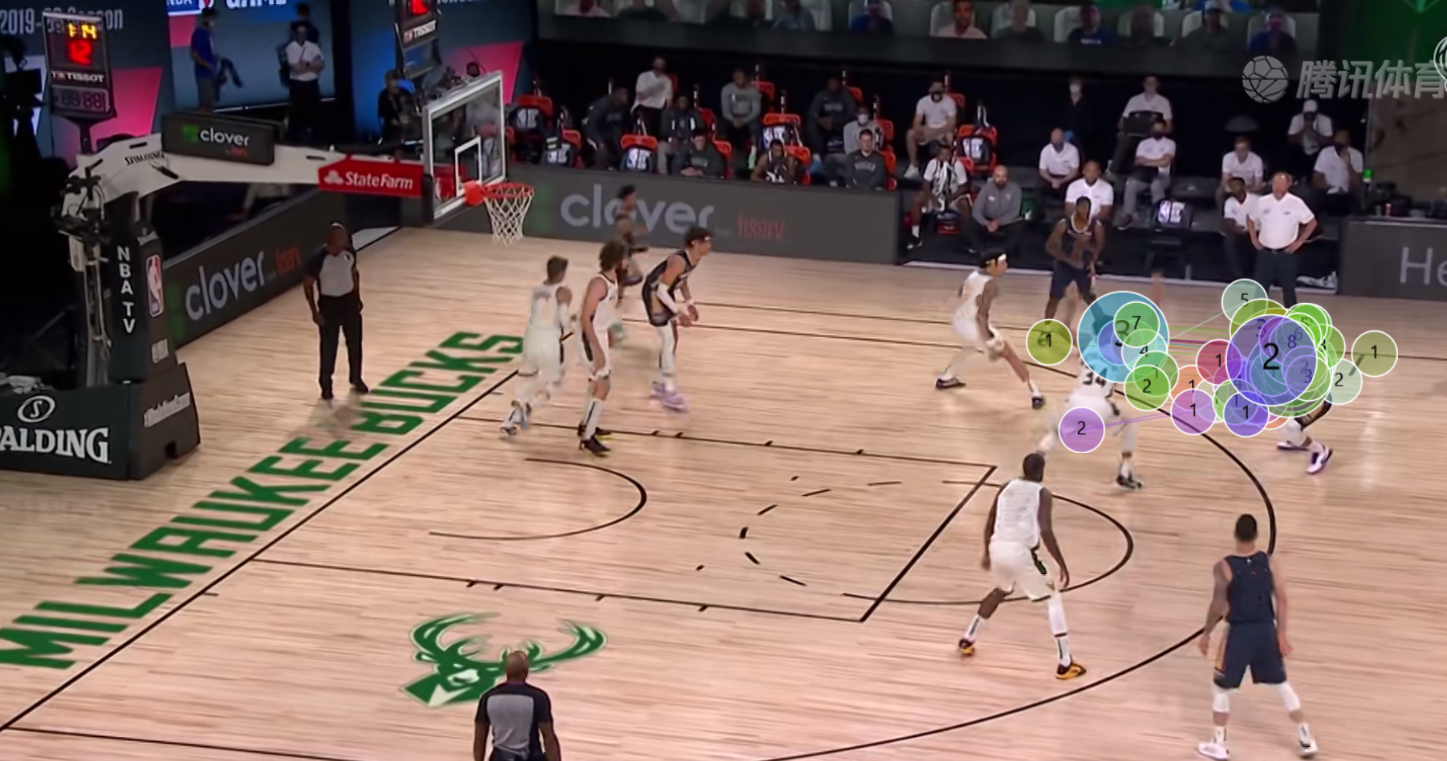

Supplement: Supplementary file 2 — Supplementary Information 2. [file 41598_2023_28754_MOESM2_ESM.zip › Gaze Plot/E15.png]

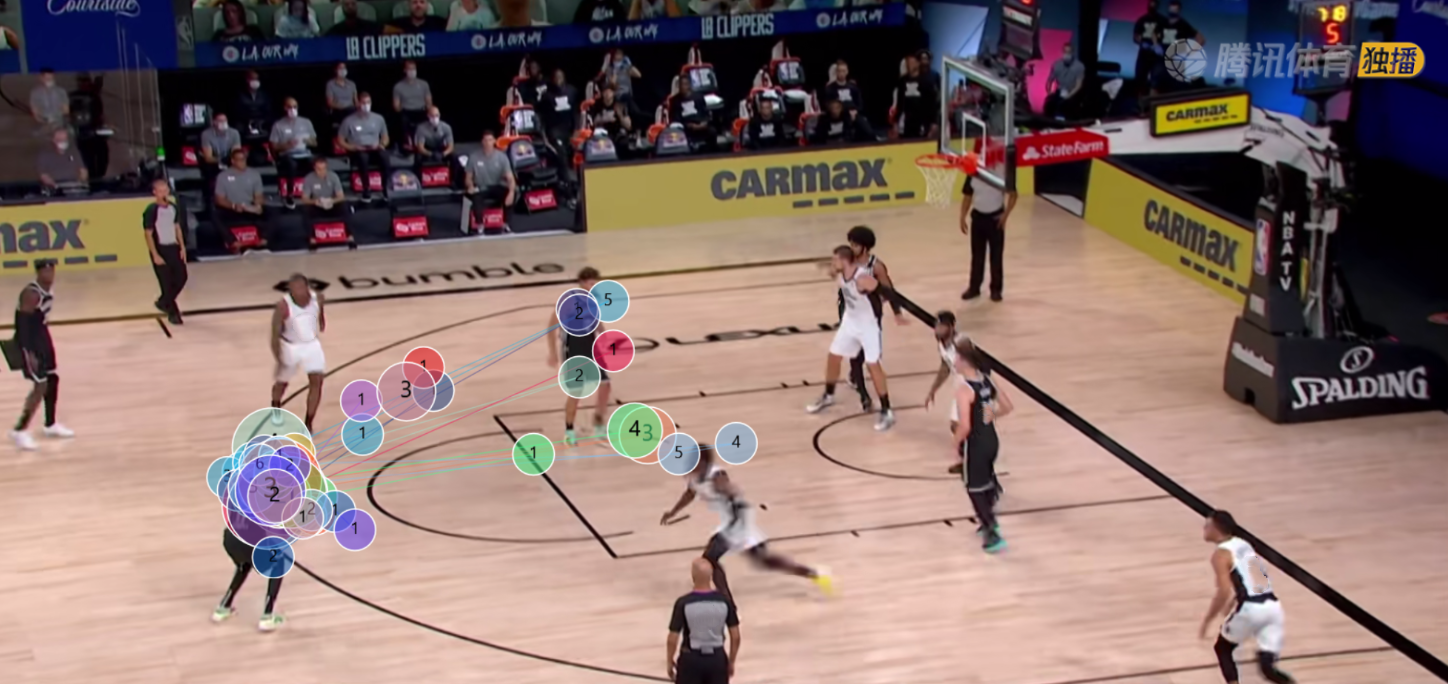

Supplement: Supplementary file 2 — Supplementary Information 2. [file 41598_2023_28754_MOESM2_ESM.zip › Gaze Plot/E16.png]

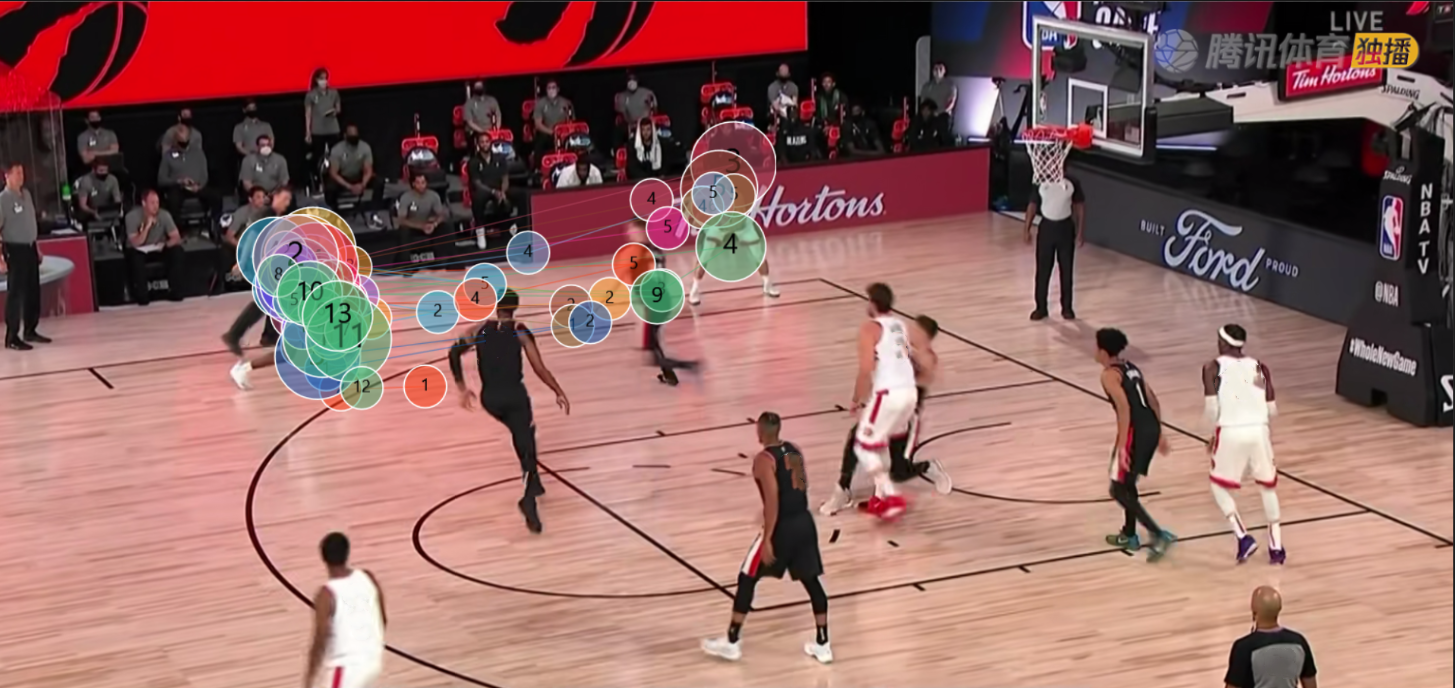

Supplement: Supplementary file 2 — Supplementary Information 2. [file 41598_2023_28754_MOESM2_ESM.zip › Gaze Plot/E17.png]

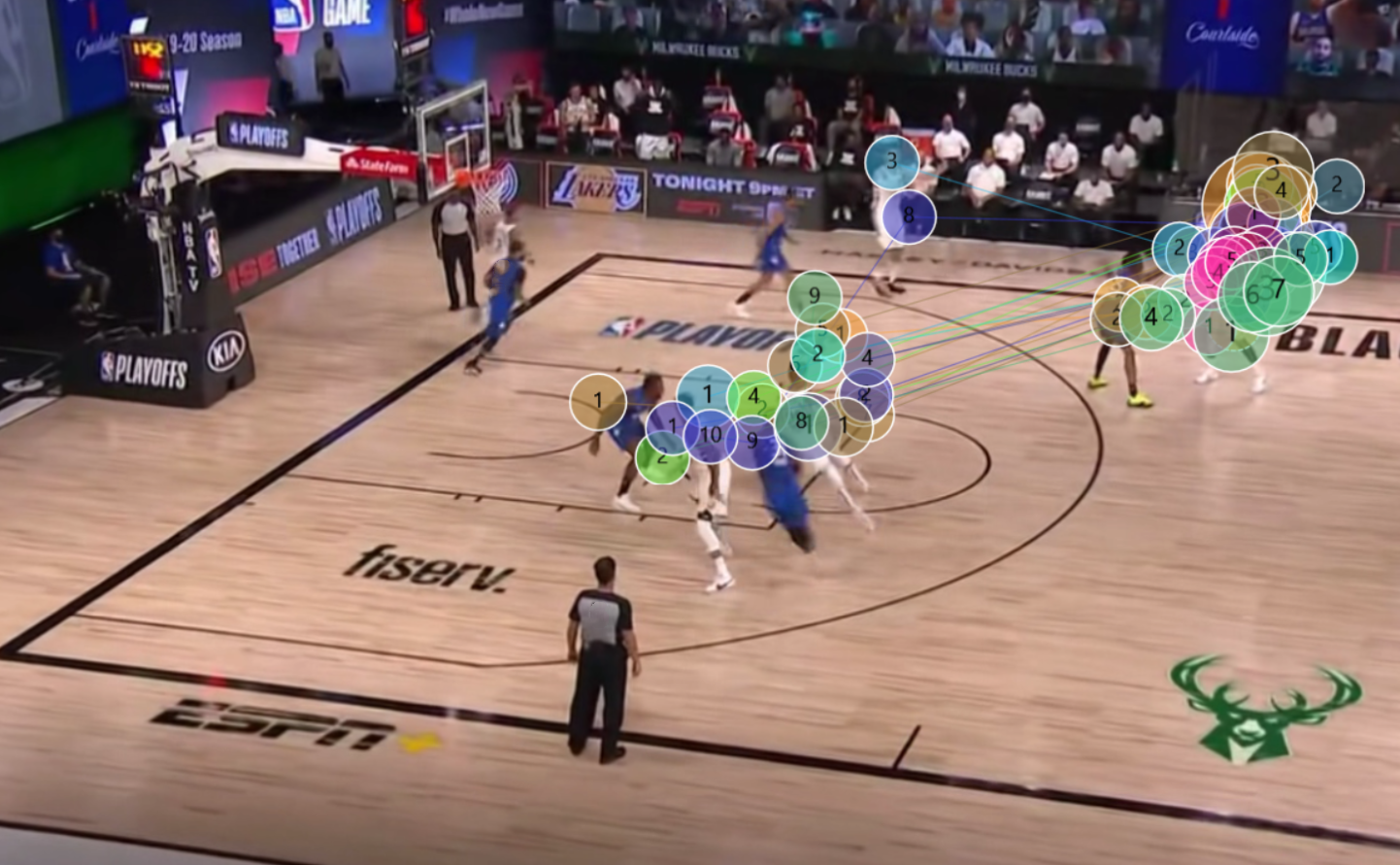

Supplement: Supplementary file 2 — Supplementary Information 2. [file 41598_2023_28754_MOESM2_ESM.zip › Gaze Plot/E18.png]

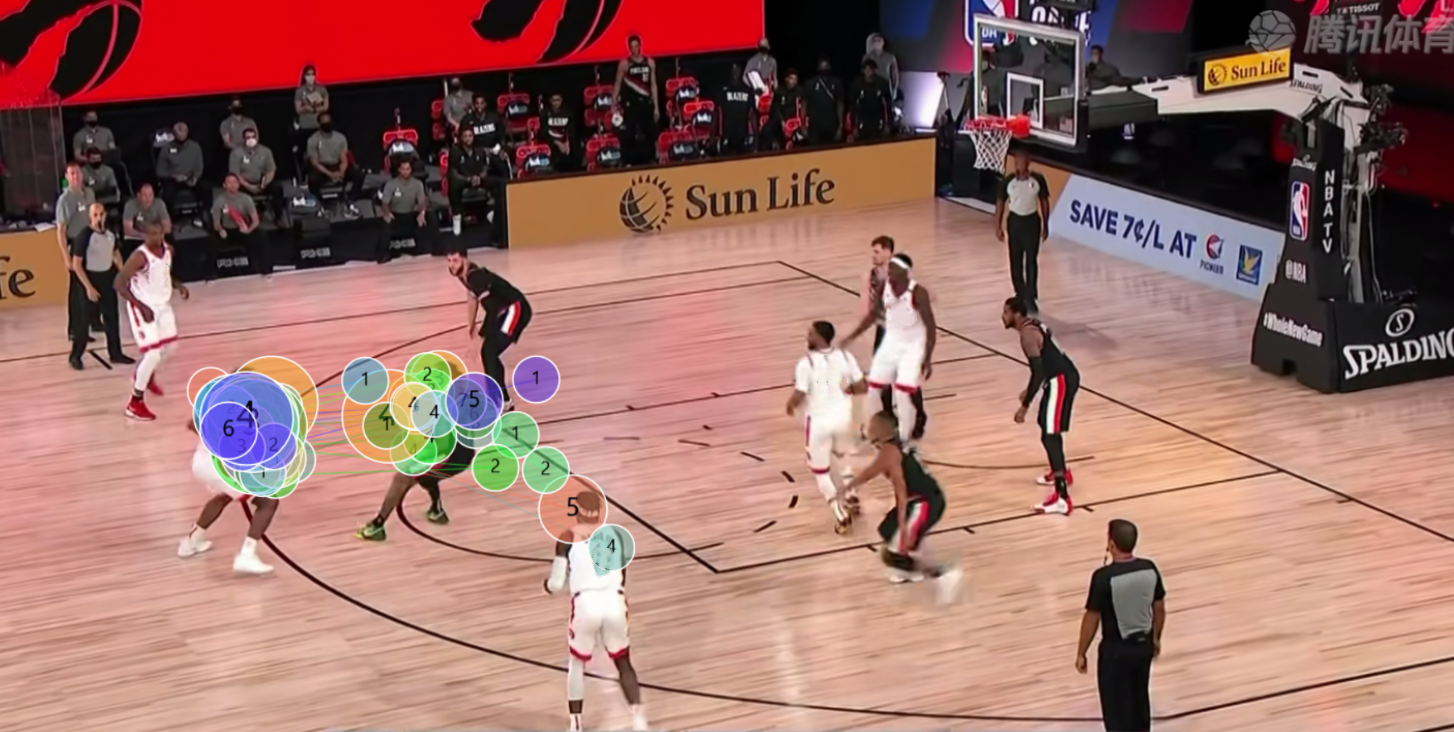

Supplement: Supplementary file 2 — Supplementary Information 2. [file 41598_2023_28754_MOESM2_ESM.zip › Gaze Plot/E19.png]

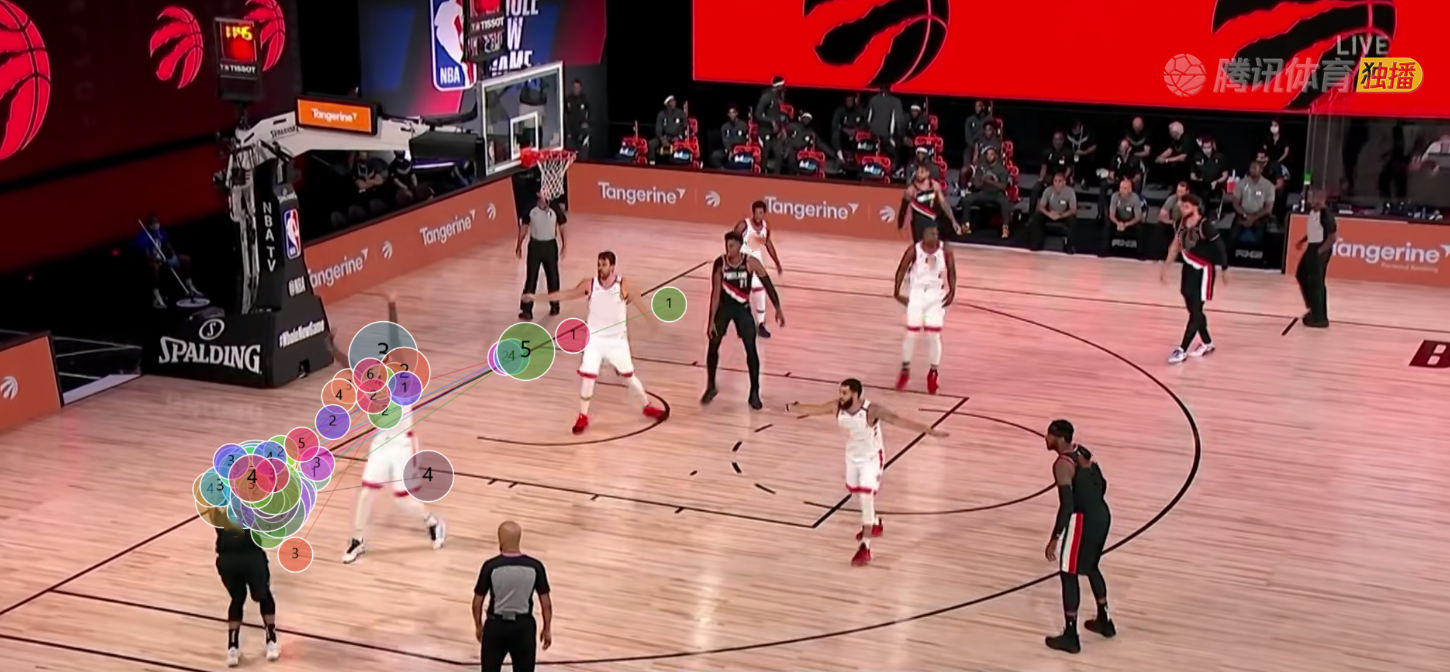

Supplement: Supplementary file 2 — Supplementary Information 2. [file 41598_2023_28754_MOESM2_ESM.zip › Gaze Plot/E2.png]

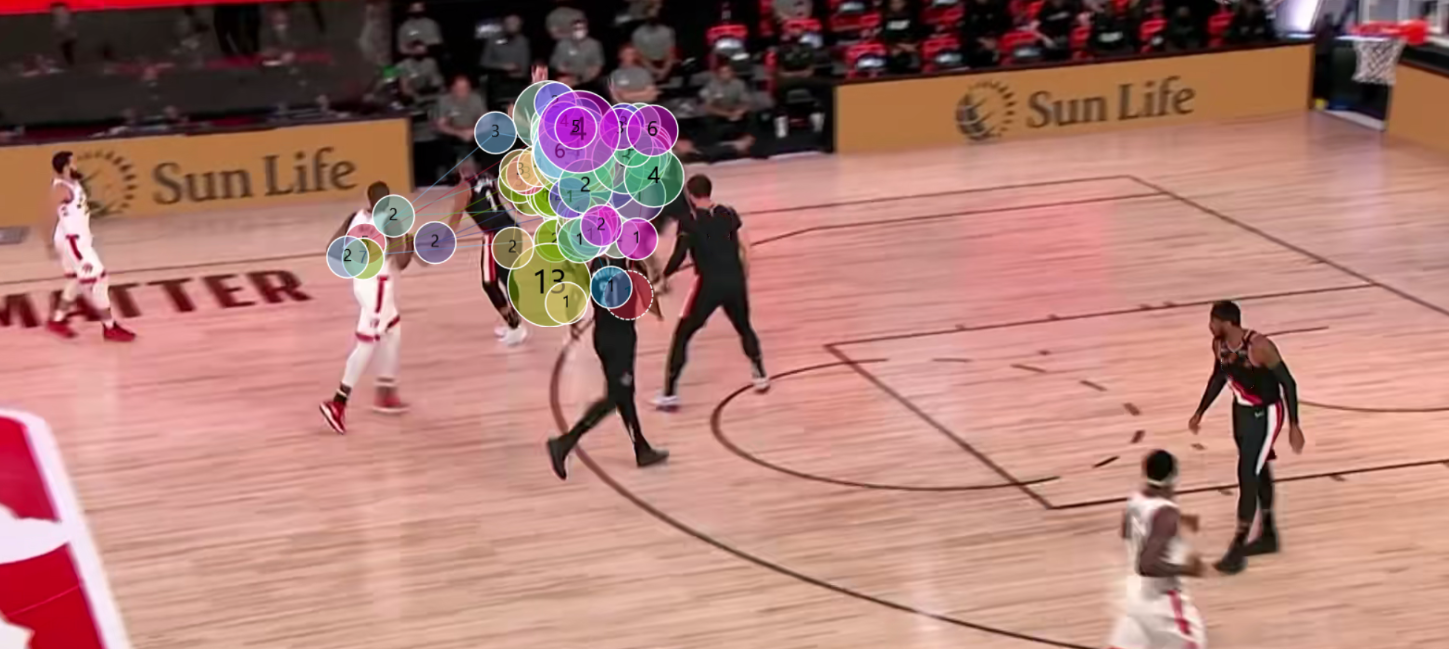

Supplement: Supplementary file 2 — Supplementary Information 2. [file 41598_2023_28754_MOESM2_ESM.zip › Gaze Plot/E20.png]

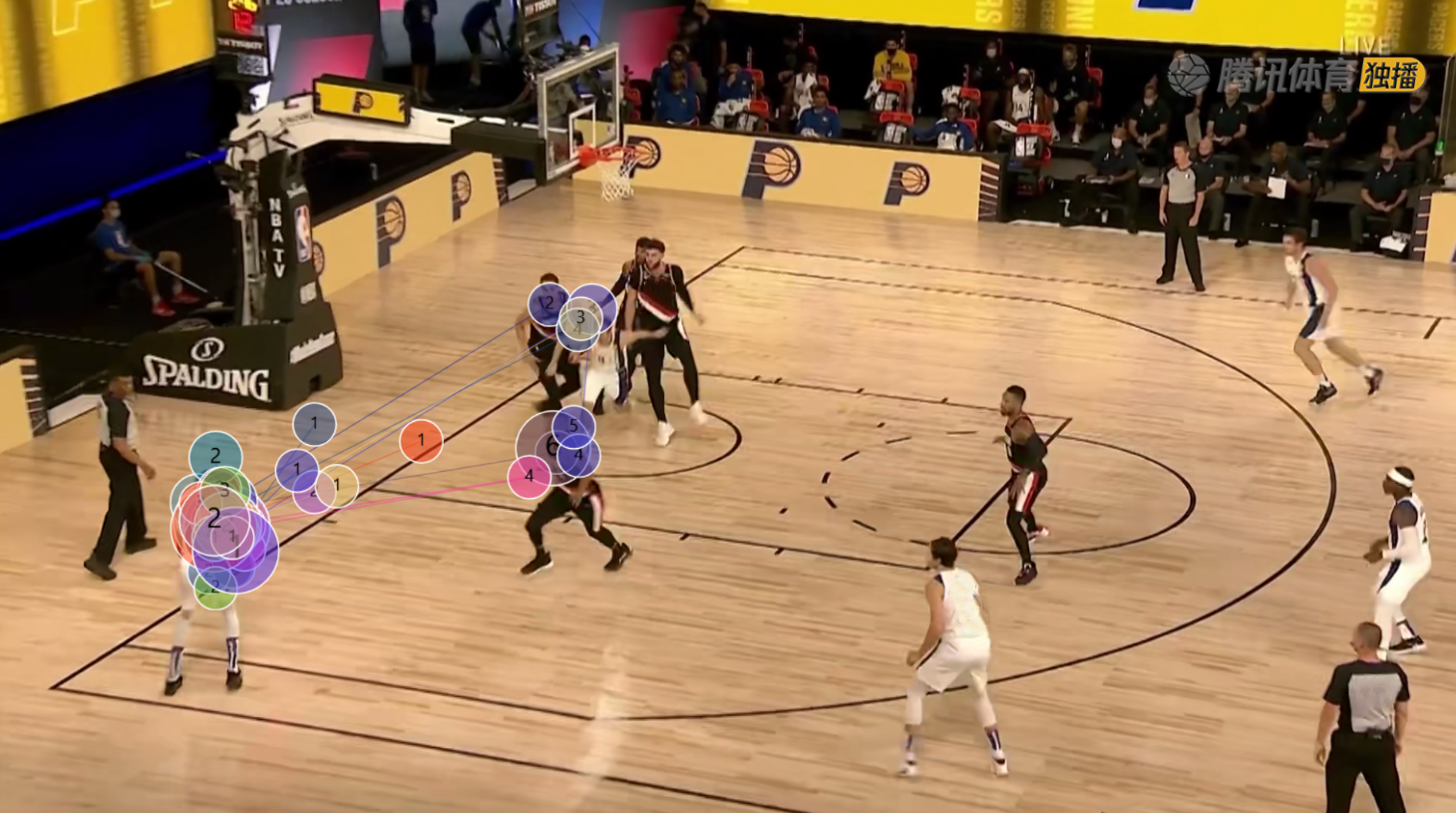

Supplement: Supplementary file 2 — Supplementary Information 2. [file 41598_2023_28754_MOESM2_ESM.zip › Gaze Plot/E21.png]

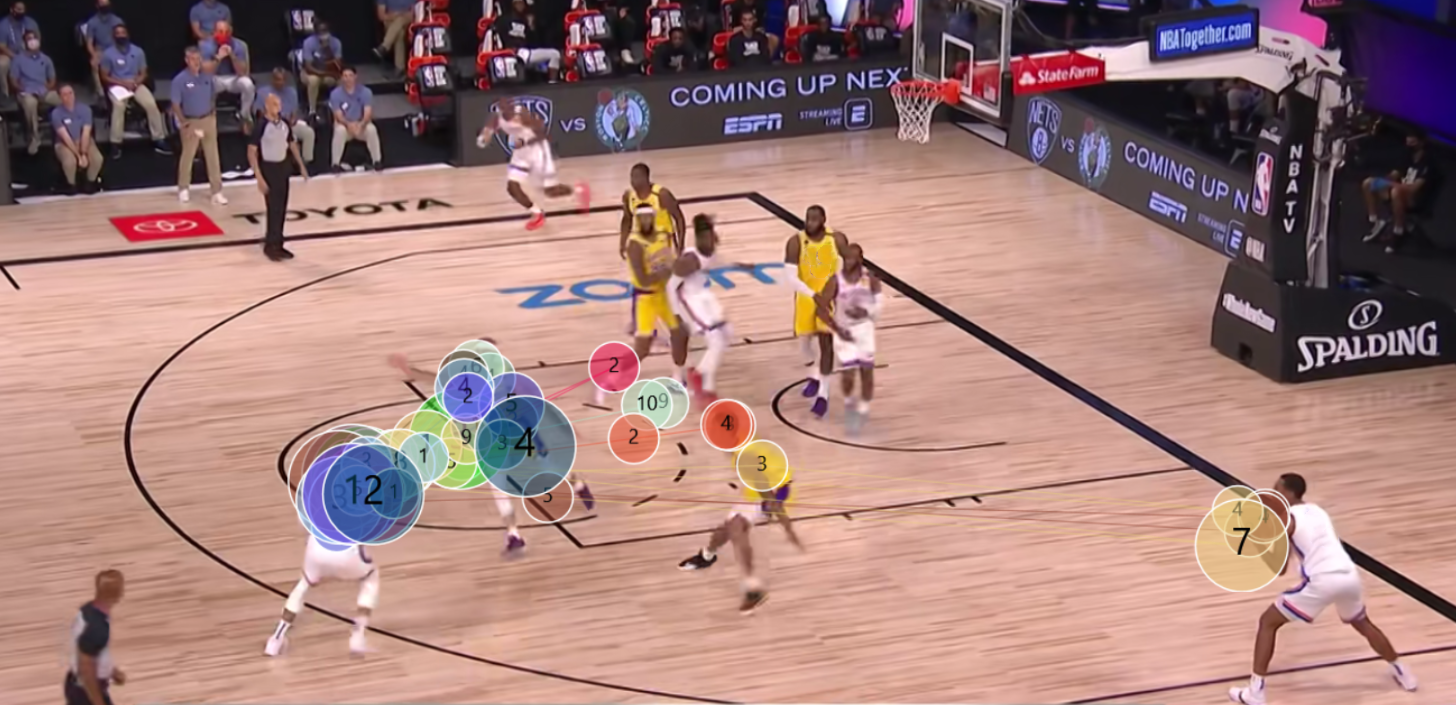

Supplement: Supplementary file 2 — Supplementary Information 2. [file 41598_2023_28754_MOESM2_ESM.zip › Gaze Plot/E3.png]

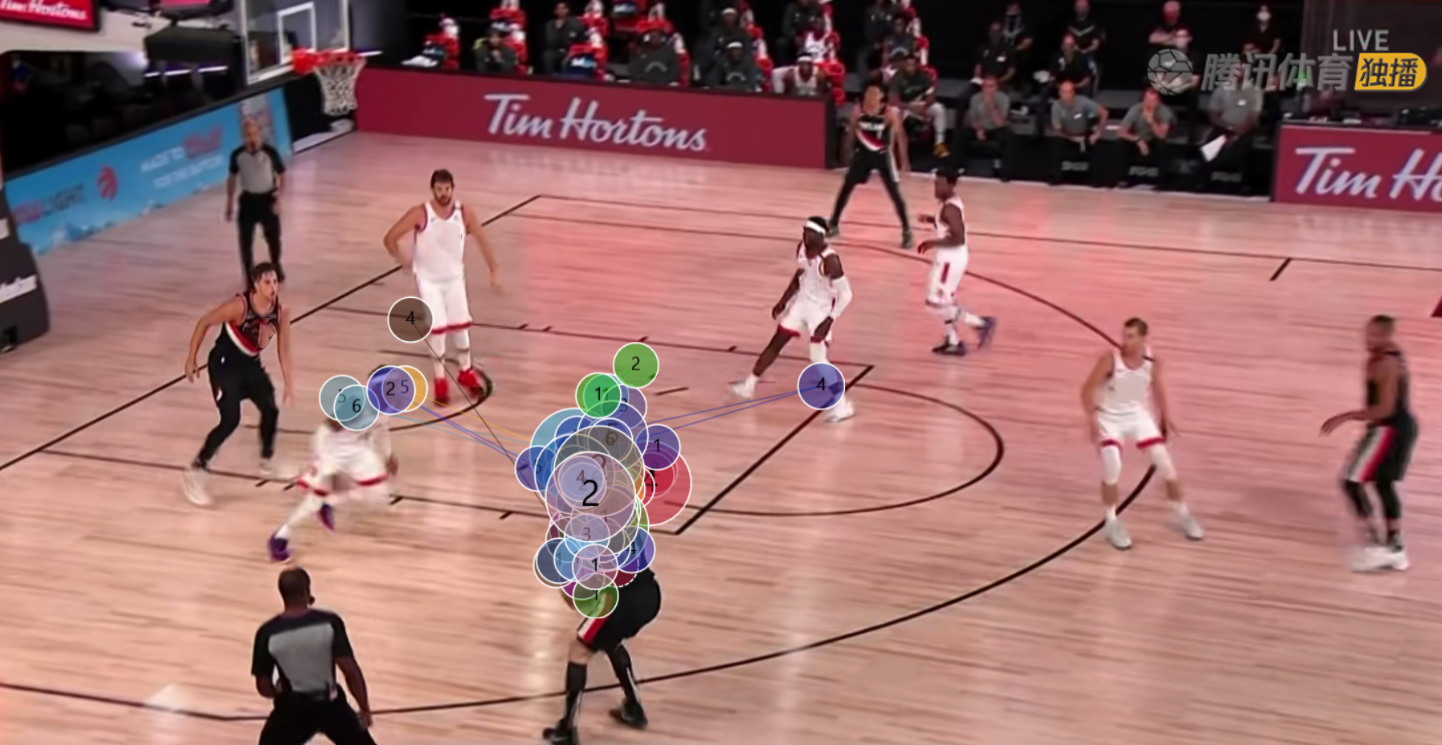

Supplement: Supplementary file 2 — Supplementary Information 2. [file 41598_2023_28754_MOESM2_ESM.zip › Gaze Plot/E4.png]

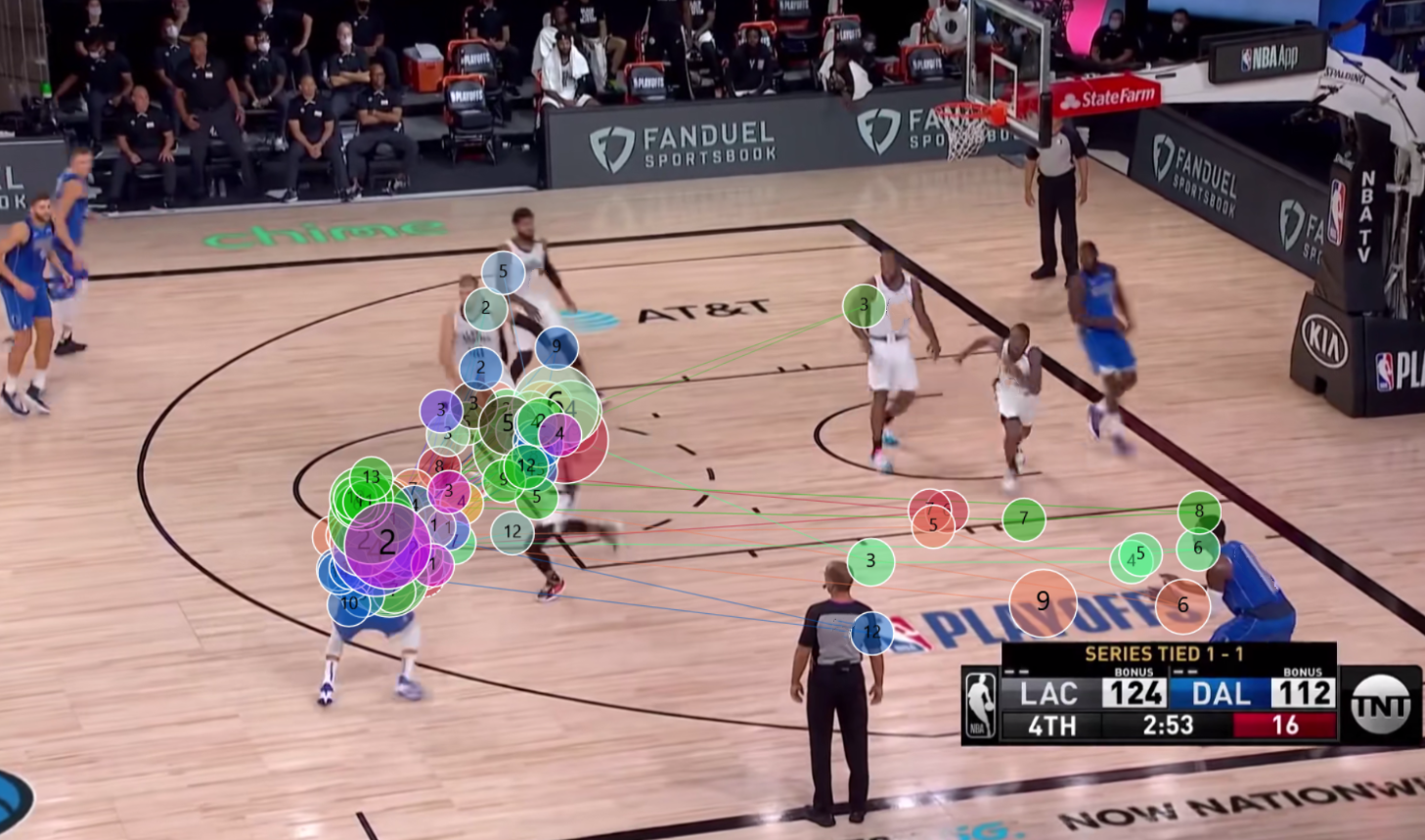

Supplement: Supplementary file 2 — Supplementary Information 2. [file 41598_2023_28754_MOESM2_ESM.zip › Gaze Plot/E5.png]

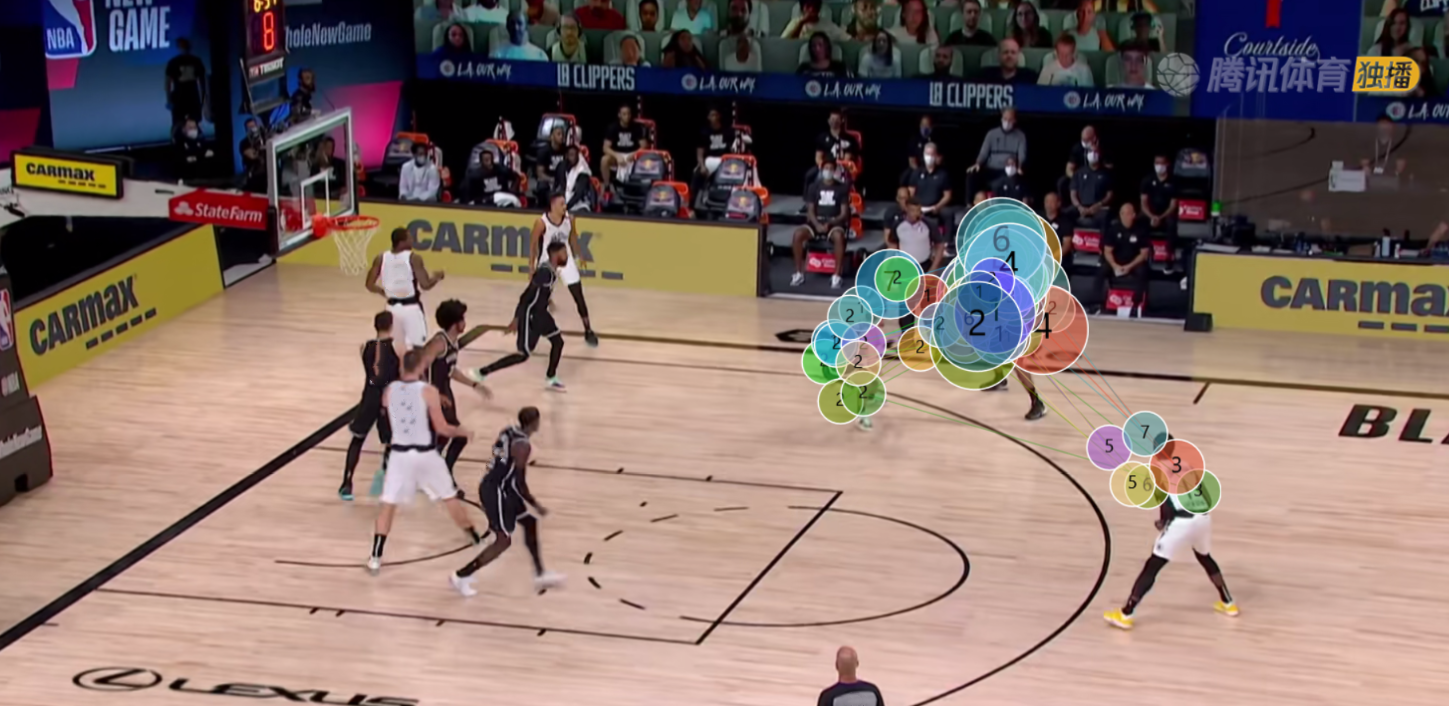

Supplement: Supplementary file 2 — Supplementary Information 2. [file 41598_2023_28754_MOESM2_ESM.zip › Gaze Plot/E6.png]

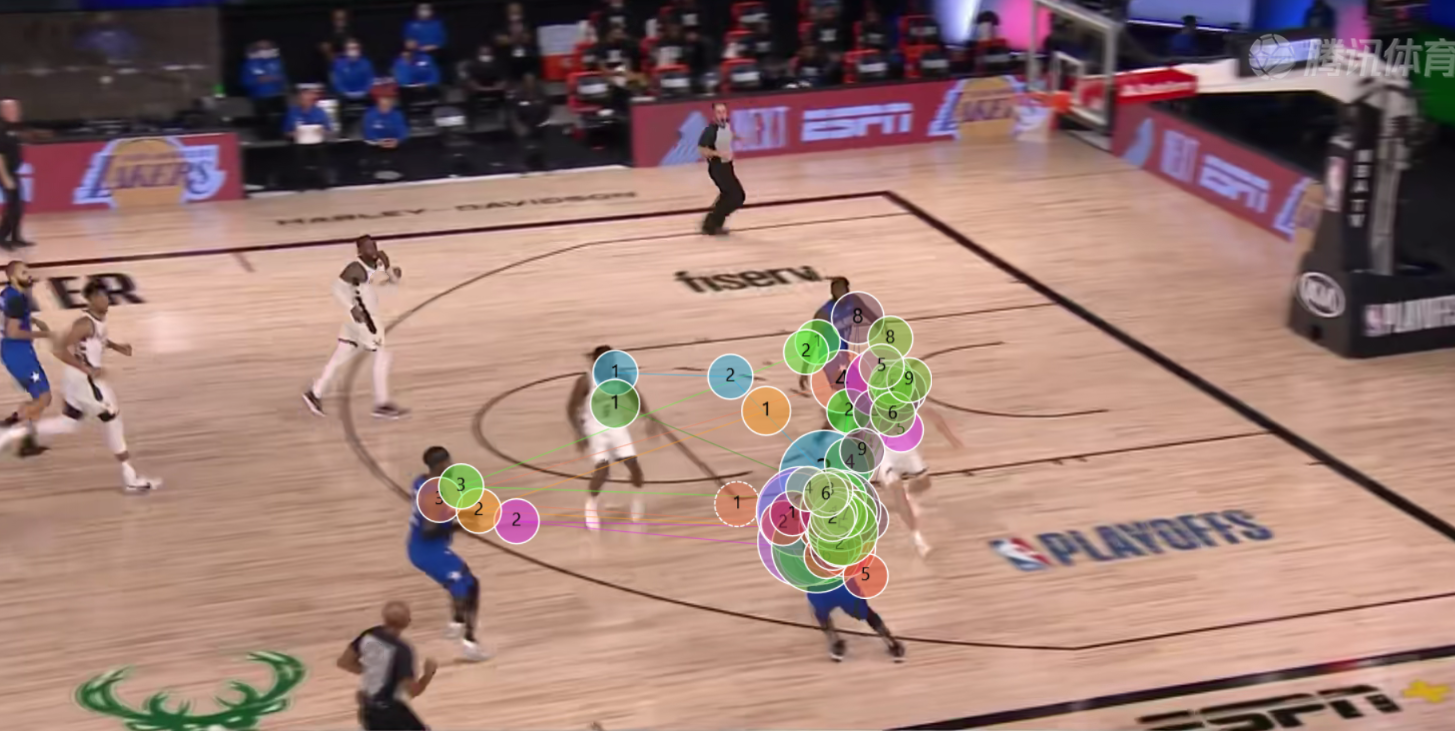

Supplement: Supplementary file 2 — Supplementary Information 2. [file 41598_2023_28754_MOESM2_ESM.zip › Gaze Plot/E7.png]

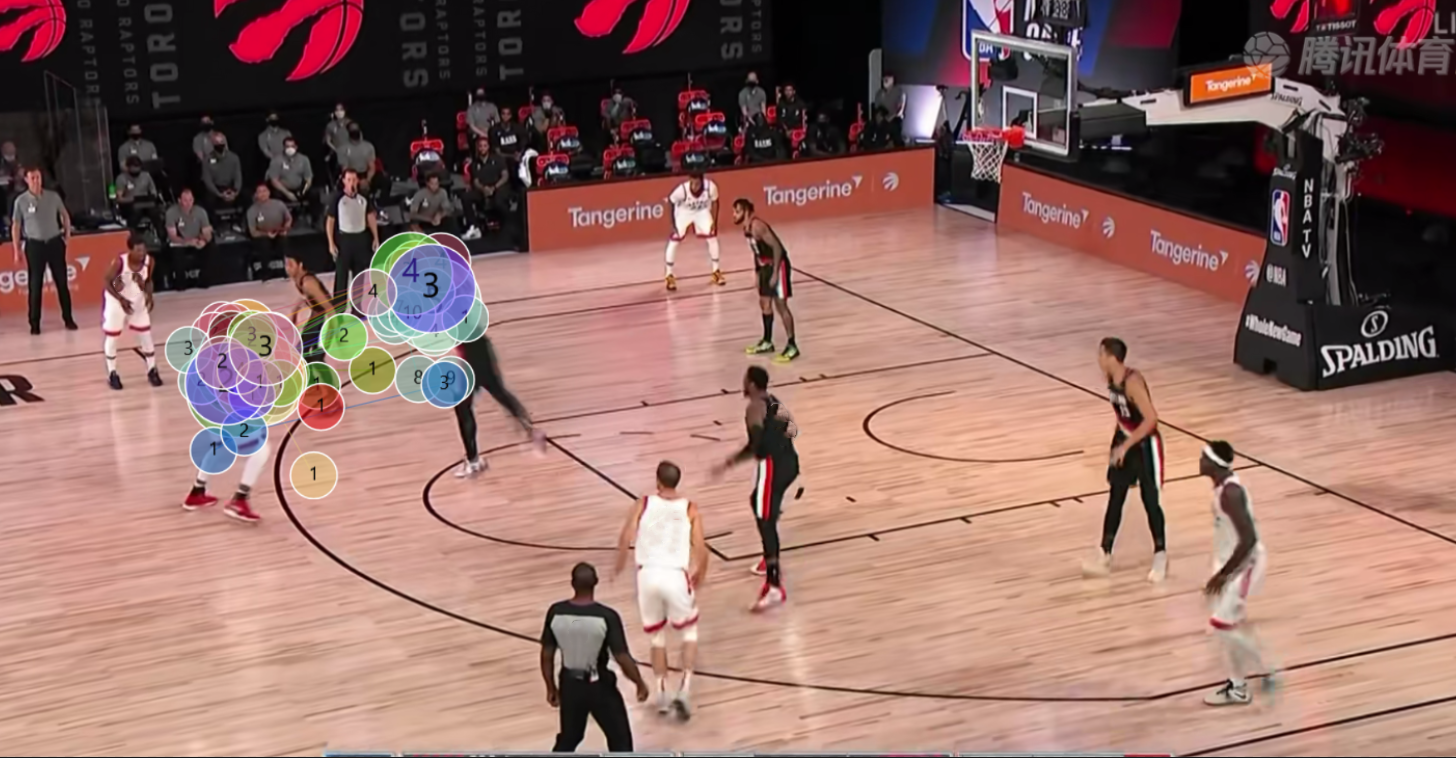

Supplement: Supplementary file 2 — Supplementary Information 2. [file 41598_2023_28754_MOESM2_ESM.zip › Gaze Plot/E8.png]

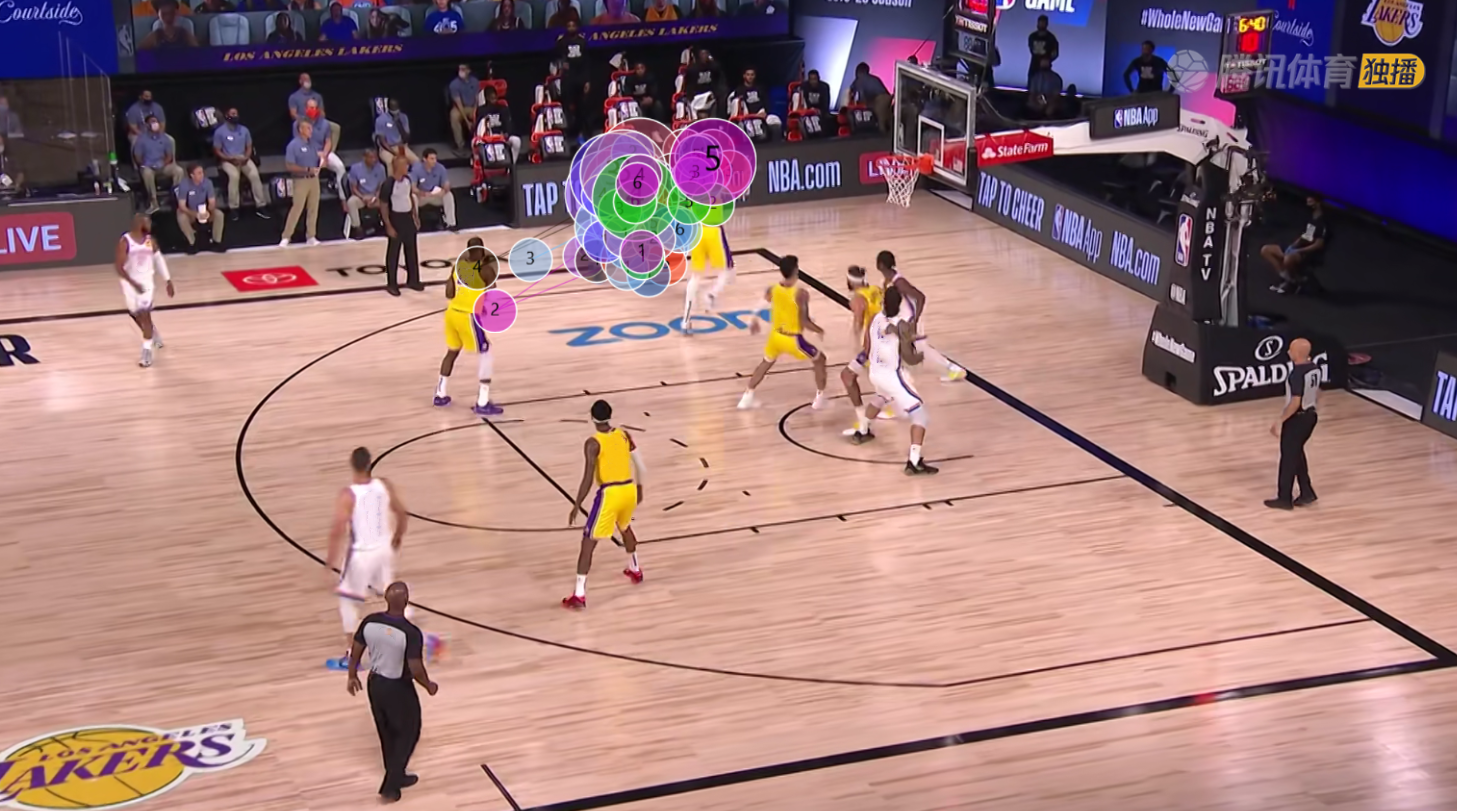

Supplement: Supplementary file 2 — Supplementary Information 2. [file 41598_2023_28754_MOESM2_ESM.zip › Gaze Plot/E9.png]

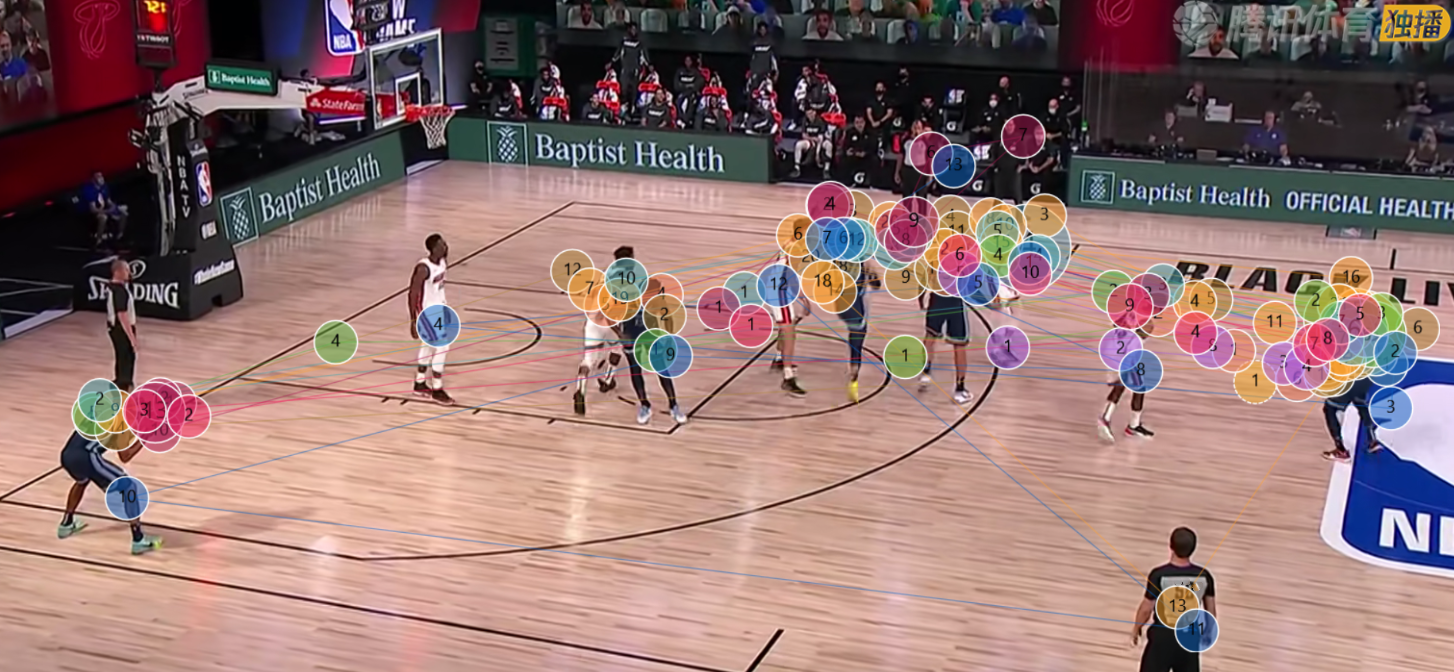

Supplement: Supplementary file 2 — Supplementary Information 2. [file 41598_2023_28754_MOESM2_ESM.zip › Gaze Plot/Expert1.png]

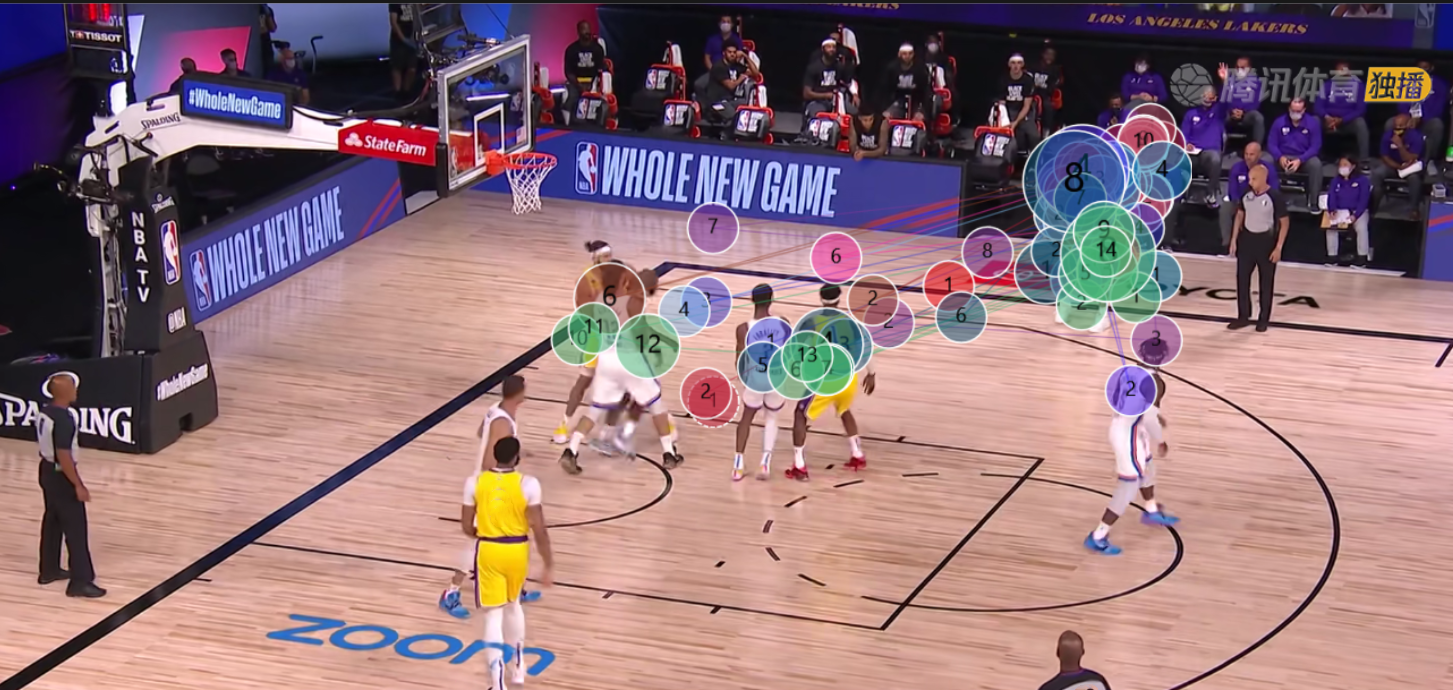

Supplement: Supplementary file 2 — Supplementary Information 2. [file 41598_2023_28754_MOESM2_ESM.zip › Gaze Plot/N10.png]

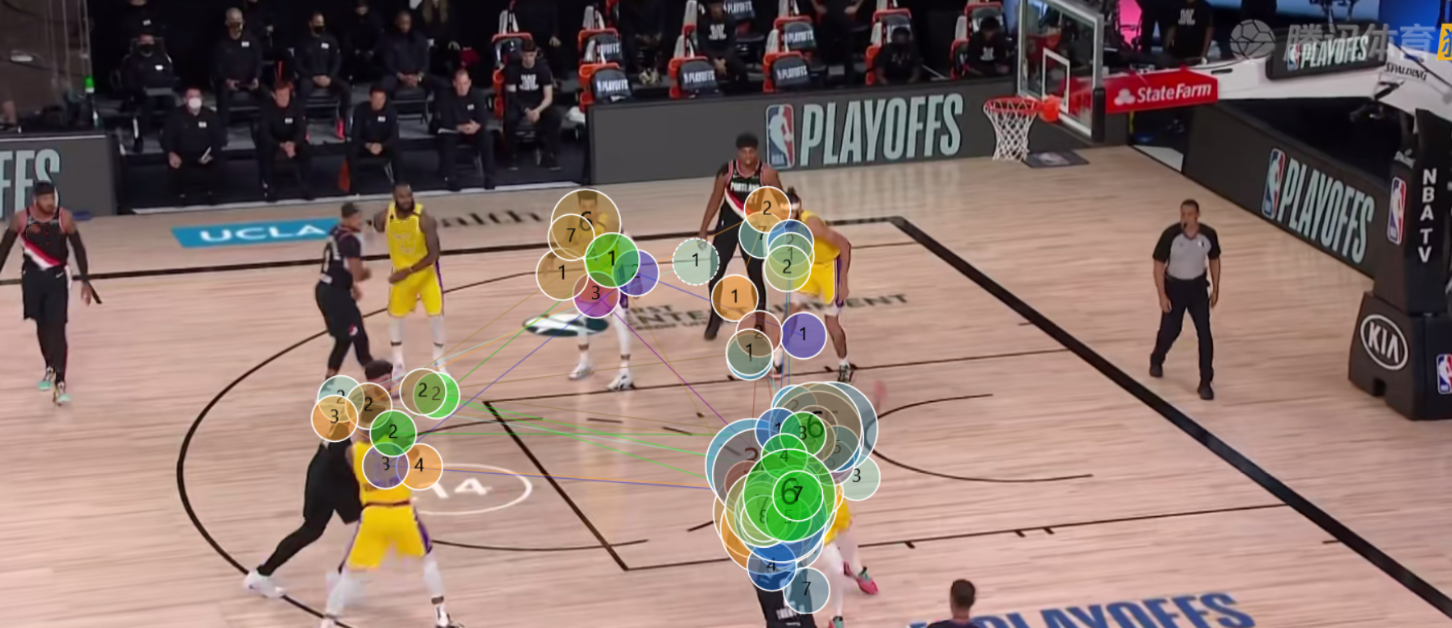

Supplement: Supplementary file 2 — Supplementary Information 2. [file 41598_2023_28754_MOESM2_ESM.zip › Gaze Plot/N11.png]

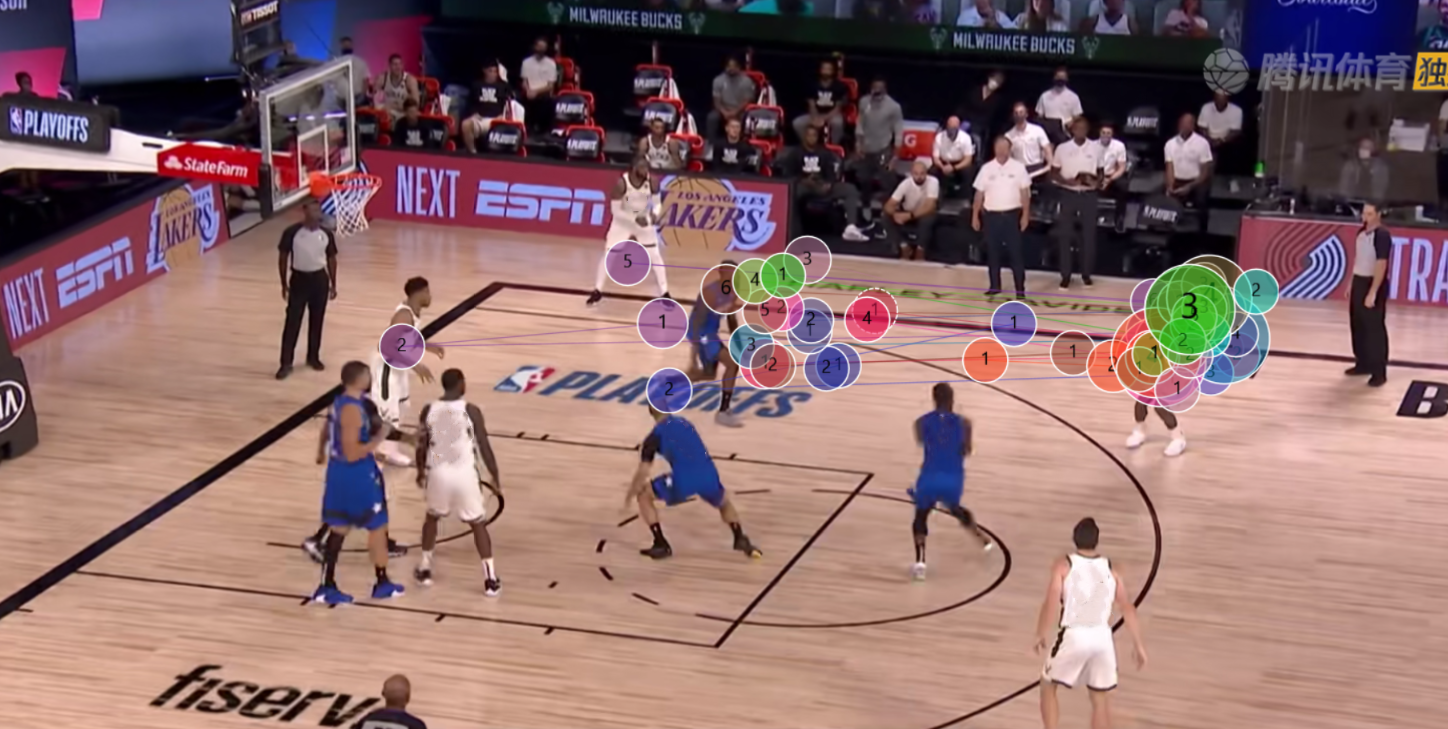

Supplement: Supplementary file 2 — Supplementary Information 2. [file 41598_2023_28754_MOESM2_ESM.zip › Gaze Plot/N12.png]

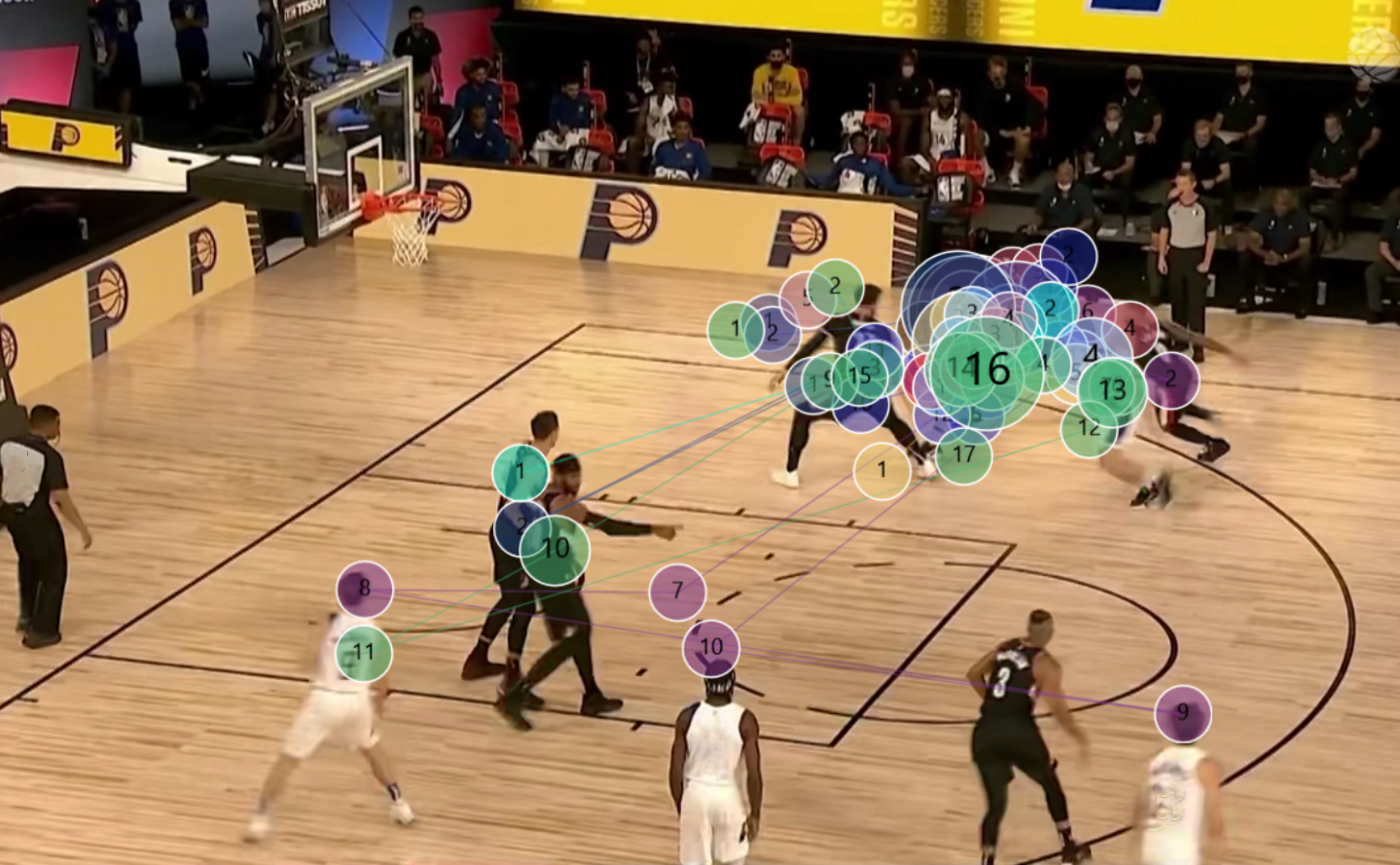

Supplement: Supplementary file 2 — Supplementary Information 2. [file 41598_2023_28754_MOESM2_ESM.zip › Gaze Plot/N13.png]

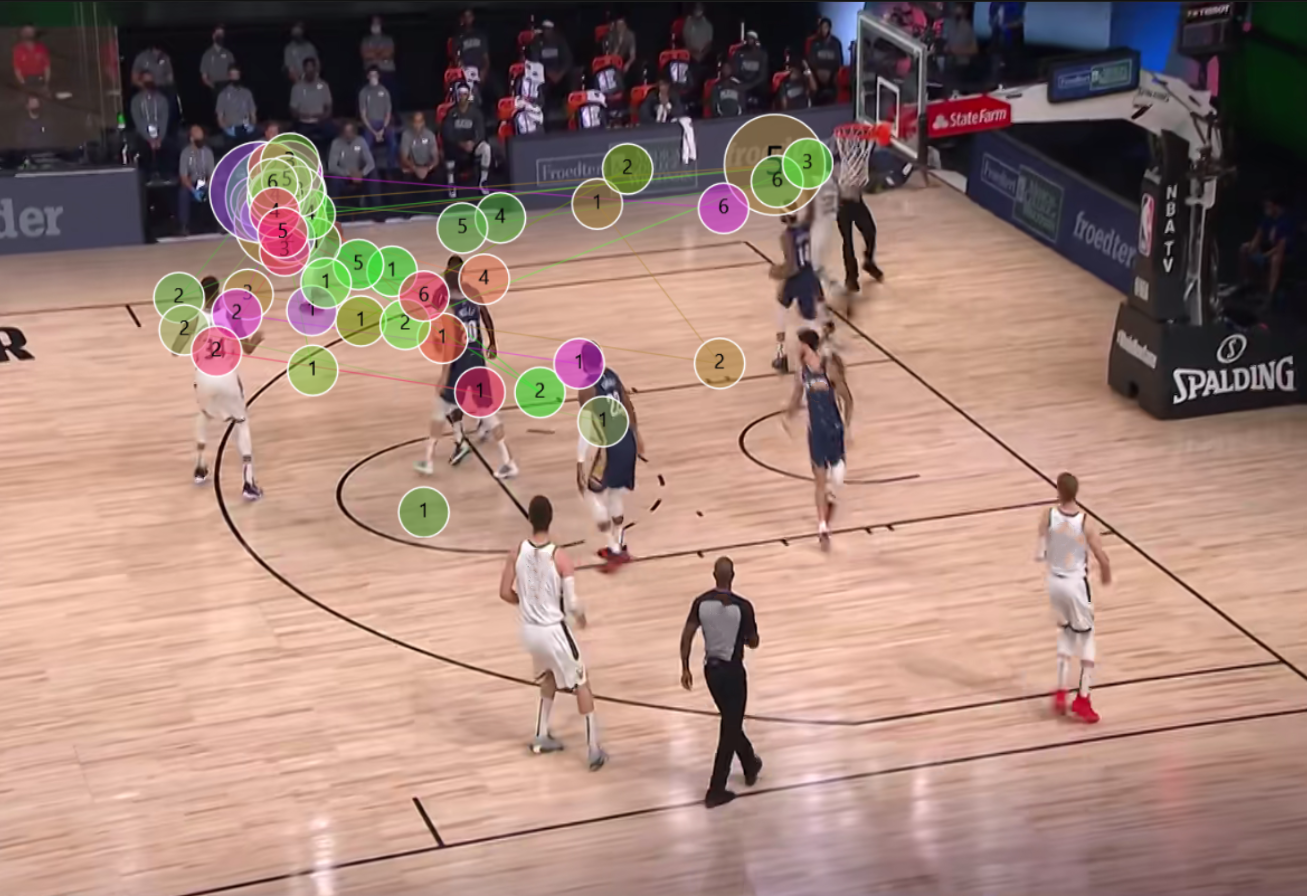

Supplement: Supplementary file 2 — Supplementary Information 2. [file 41598_2023_28754_MOESM2_ESM.zip › Gaze Plot/N14.png]

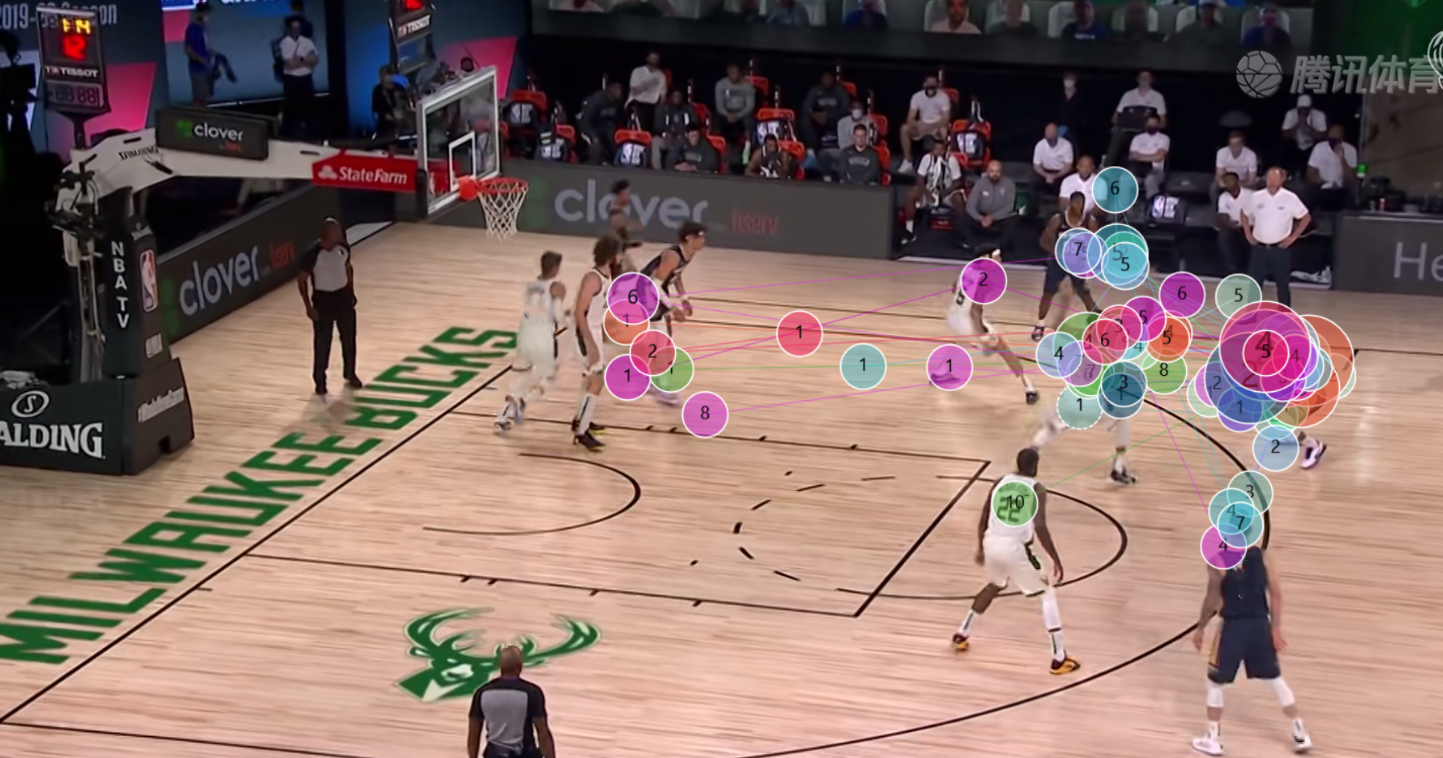

Supplement: Supplementary file 2 — Supplementary Information 2. [file 41598_2023_28754_MOESM2_ESM.zip › Gaze Plot/N15.png]

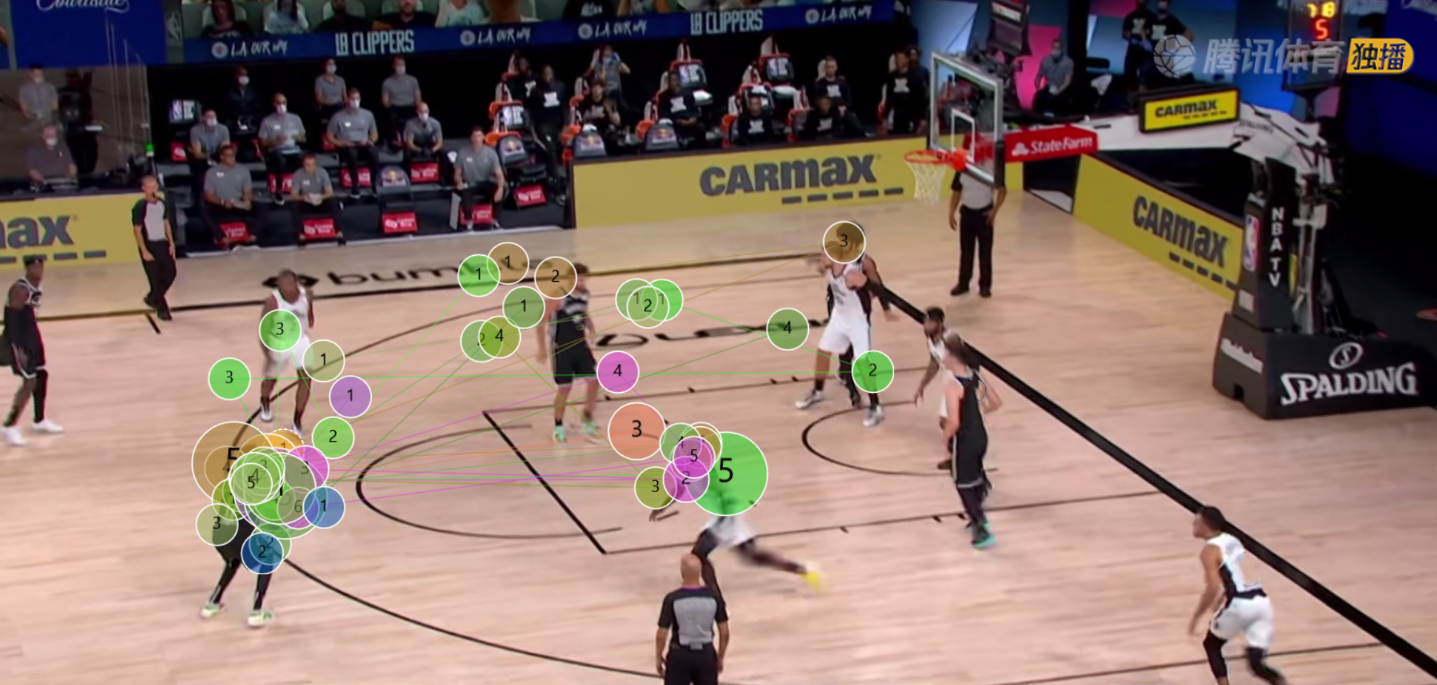

Supplement: Supplementary file 2 — Supplementary Information 2. [file 41598_2023_28754_MOESM2_ESM.zip › Gaze Plot/N16.png]

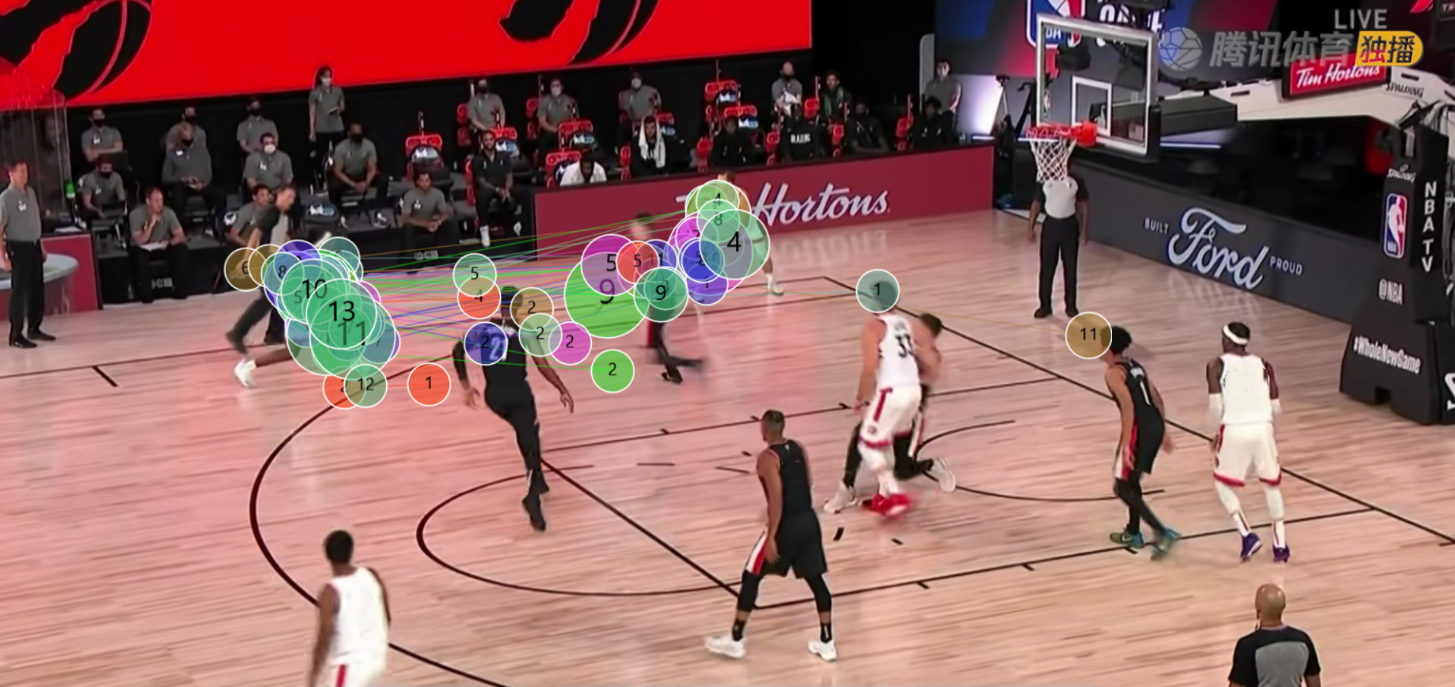

Supplement: Supplementary file 2 — Supplementary Information 2. [file 41598_2023_28754_MOESM2_ESM.zip › Gaze Plot/N17.png]

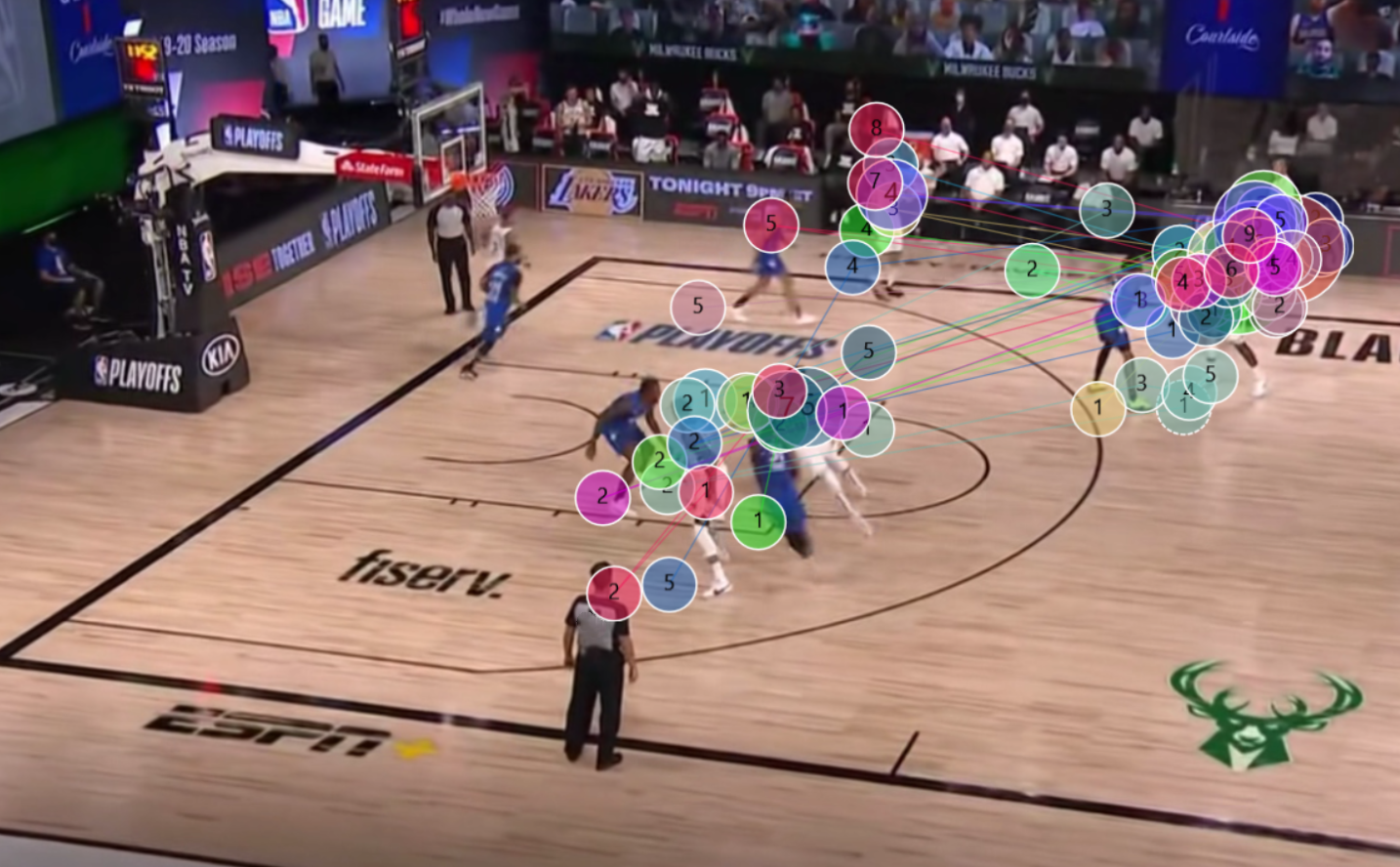

Supplement: Supplementary file 2 — Supplementary Information 2. [file 41598_2023_28754_MOESM2_ESM.zip › Gaze Plot/N18.png]

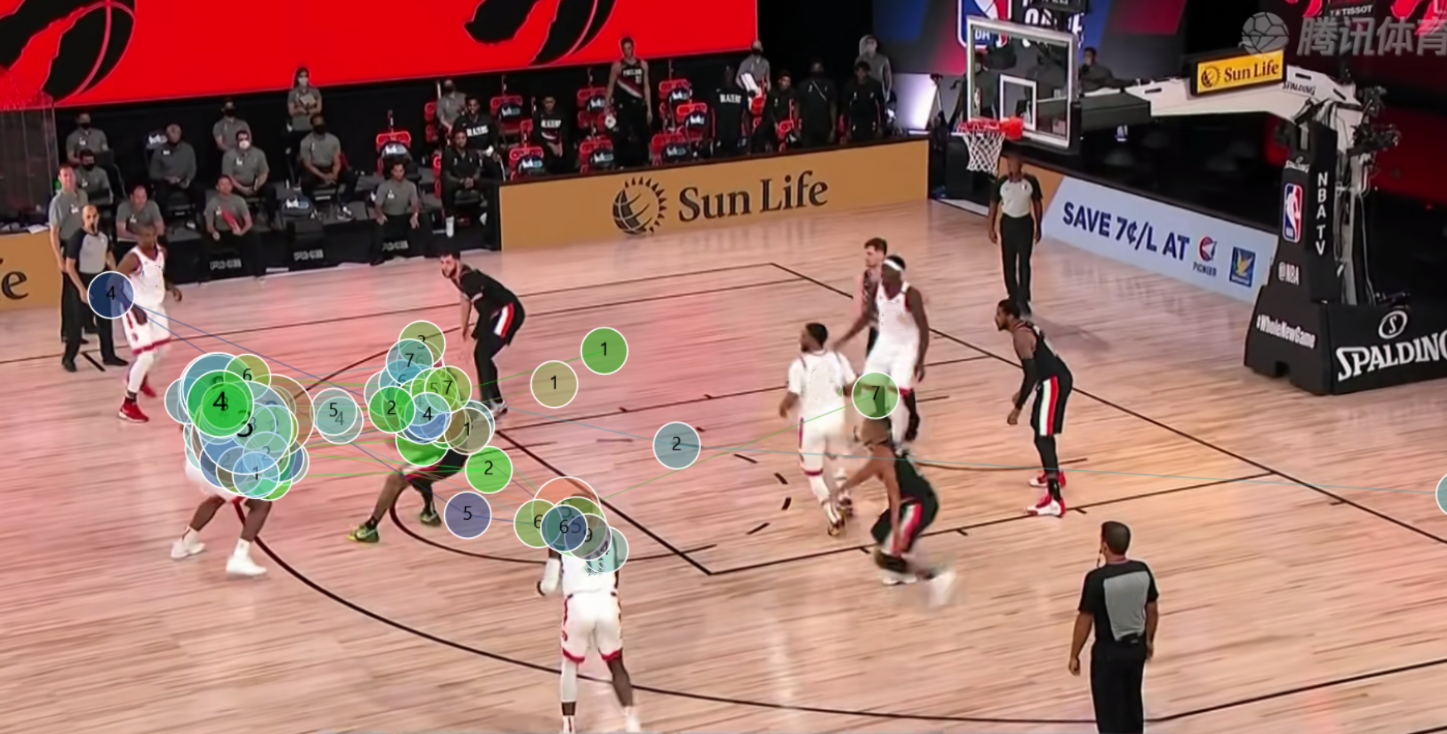

Supplement: Supplementary file 2 — Supplementary Information 2. [file 41598_2023_28754_MOESM2_ESM.zip › Gaze Plot/N19.png]

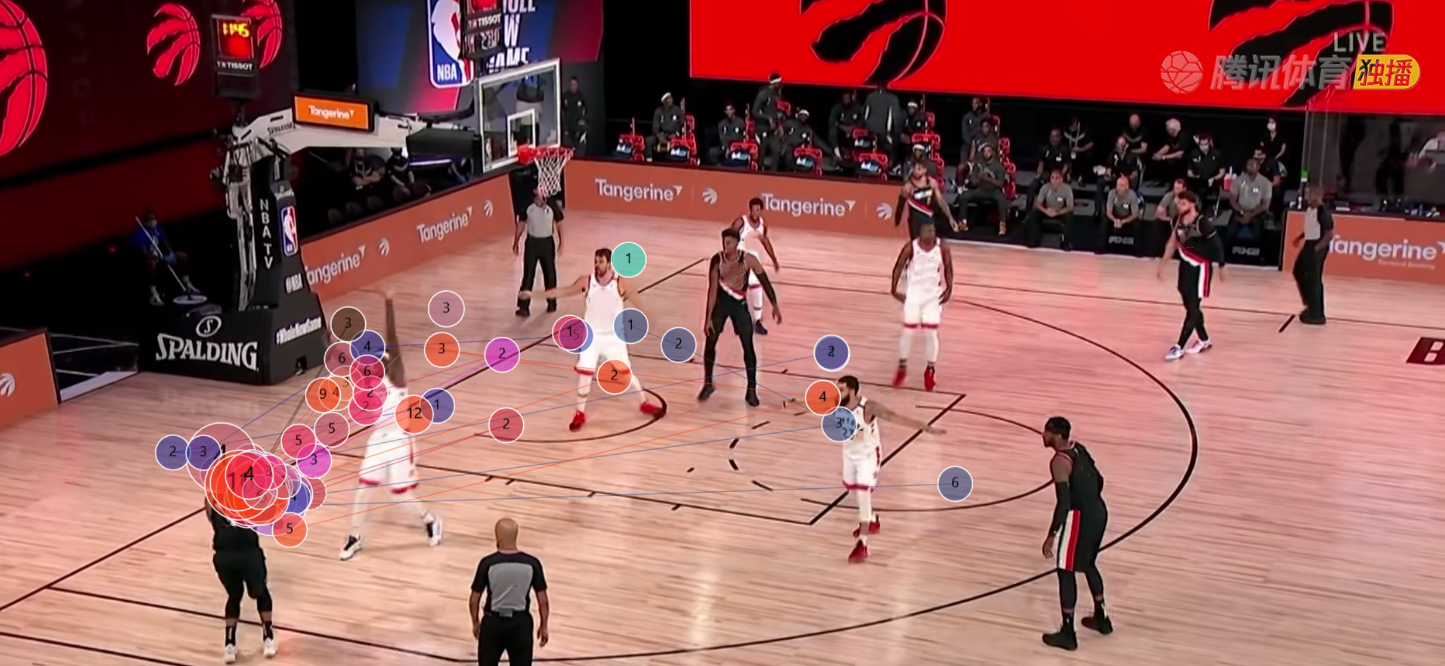

Supplement: Supplementary file 2 — Supplementary Information 2. [file 41598_2023_28754_MOESM2_ESM.zip › Gaze Plot/N2.png]

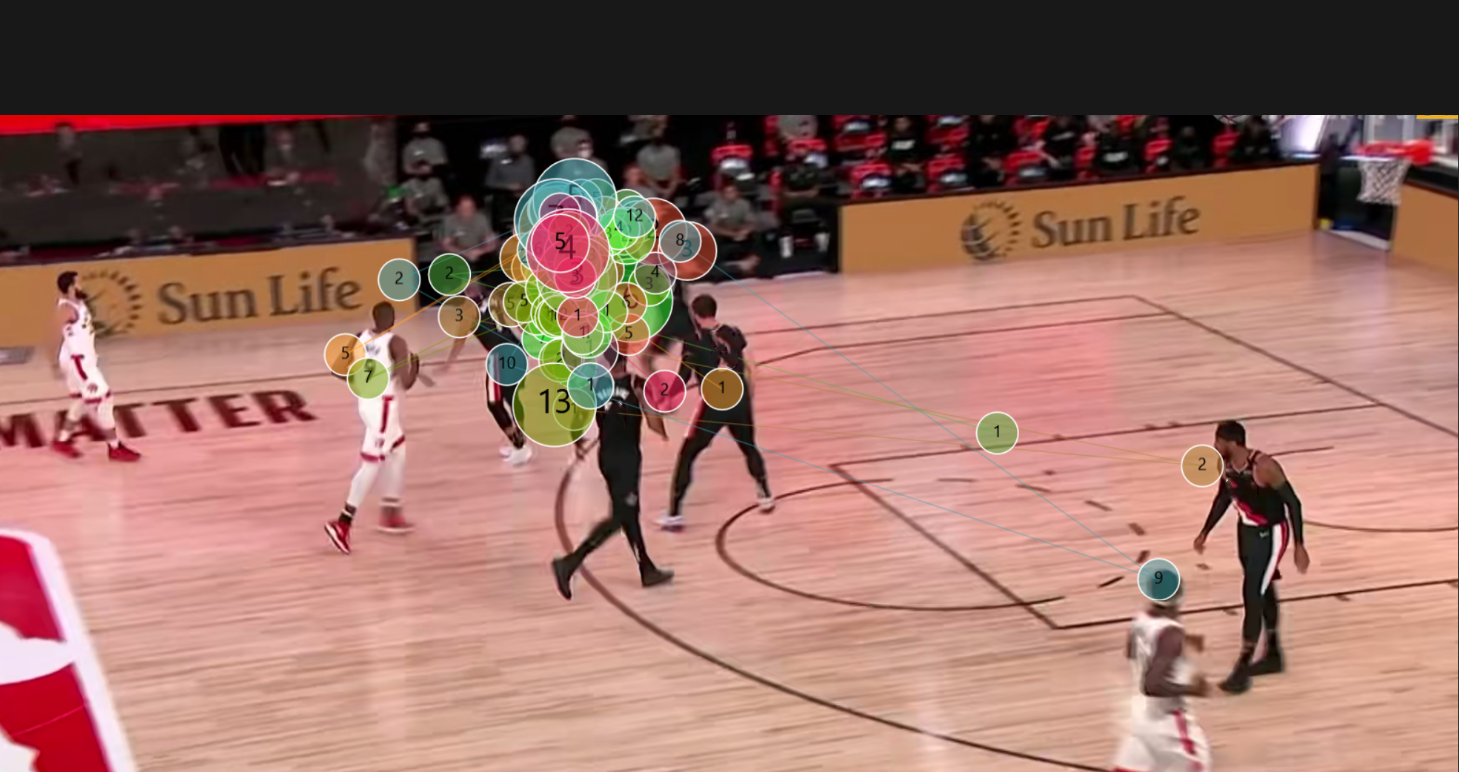

Supplement: Supplementary file 2 — Supplementary Information 2. [file 41598_2023_28754_MOESM2_ESM.zip › Gaze Plot/N20.png]

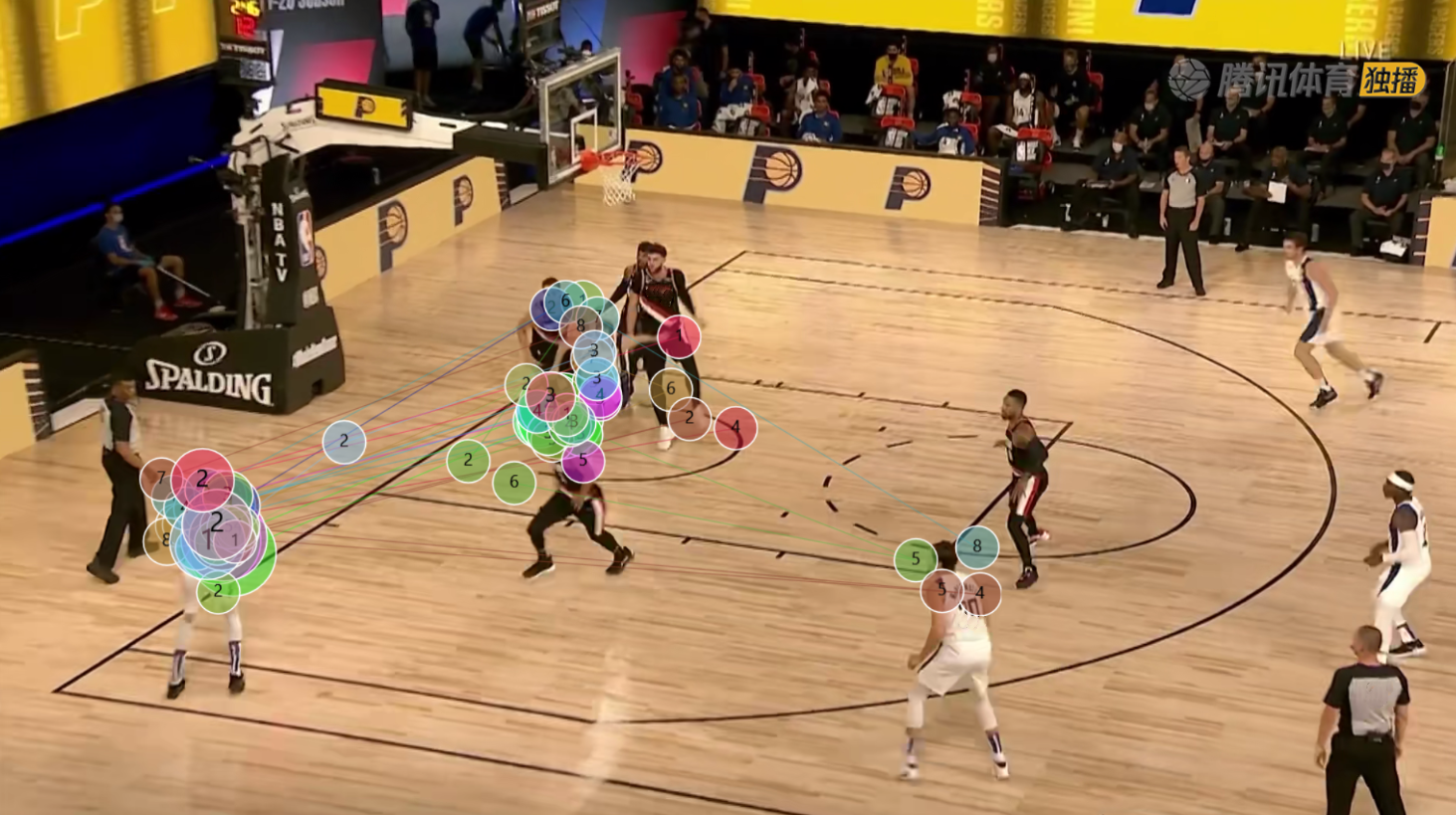

Supplement: Supplementary file 2 — Supplementary Information 2. [file 41598_2023_28754_MOESM2_ESM.zip › Gaze Plot/N21.png]

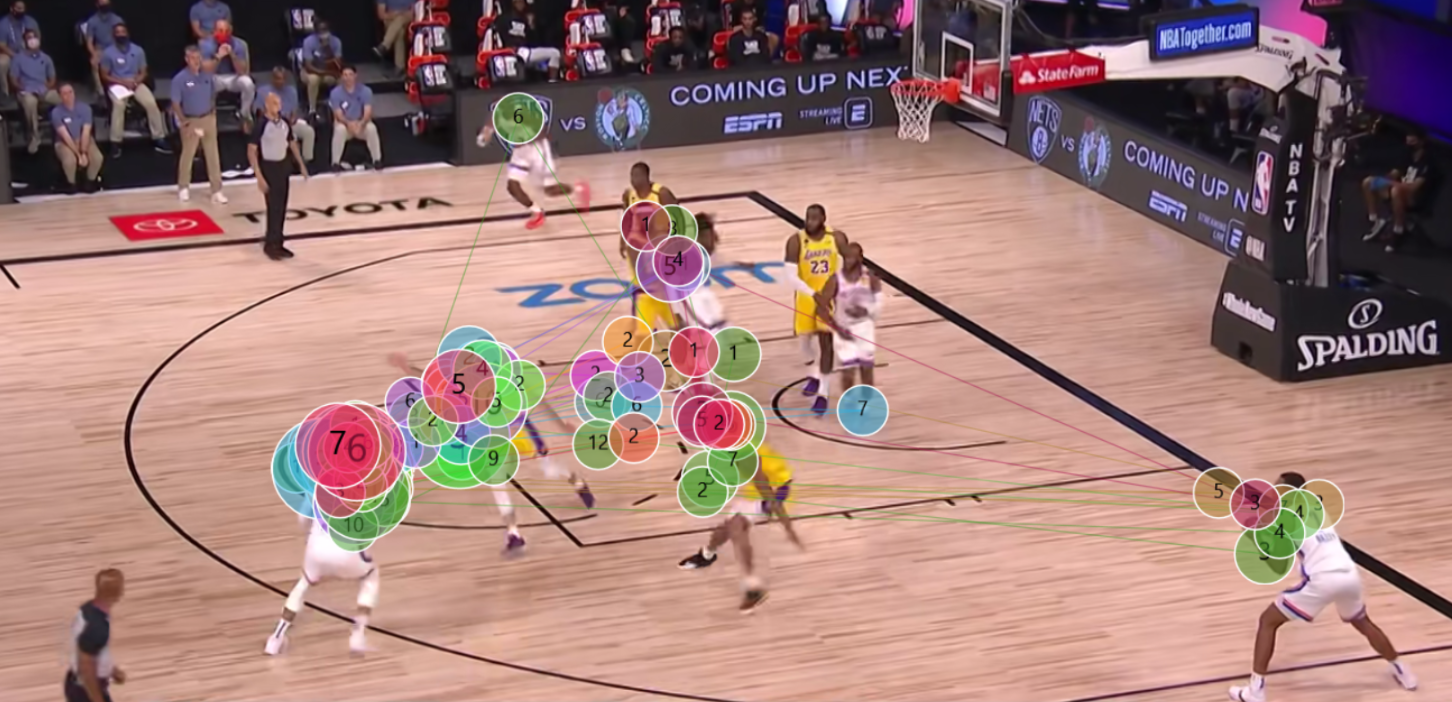

Supplement: Supplementary file 2 — Supplementary Information 2. [file 41598_2023_28754_MOESM2_ESM.zip › Gaze Plot/N3.png]

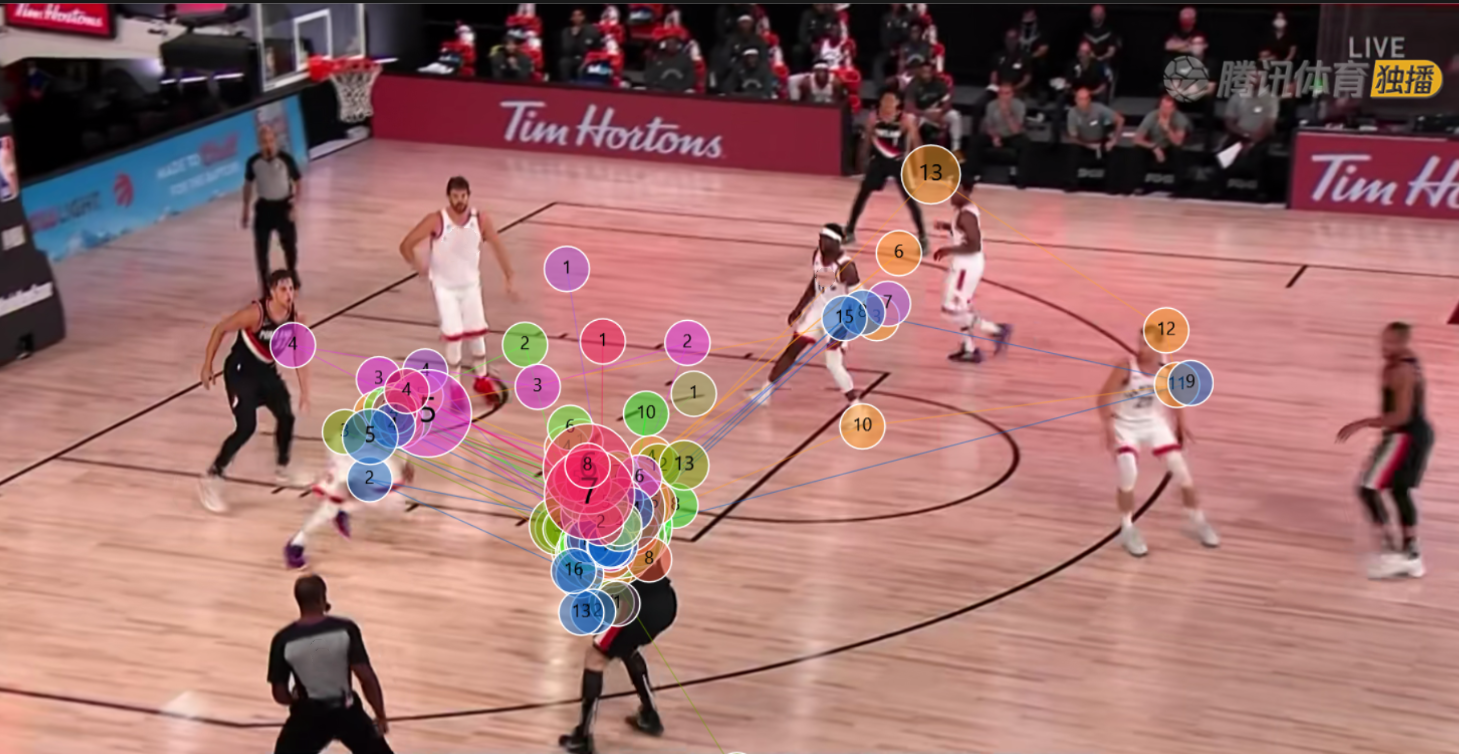

Supplement: Supplementary file 2 — Supplementary Information 2. [file 41598_2023_28754_MOESM2_ESM.zip › Gaze Plot/N4.png]

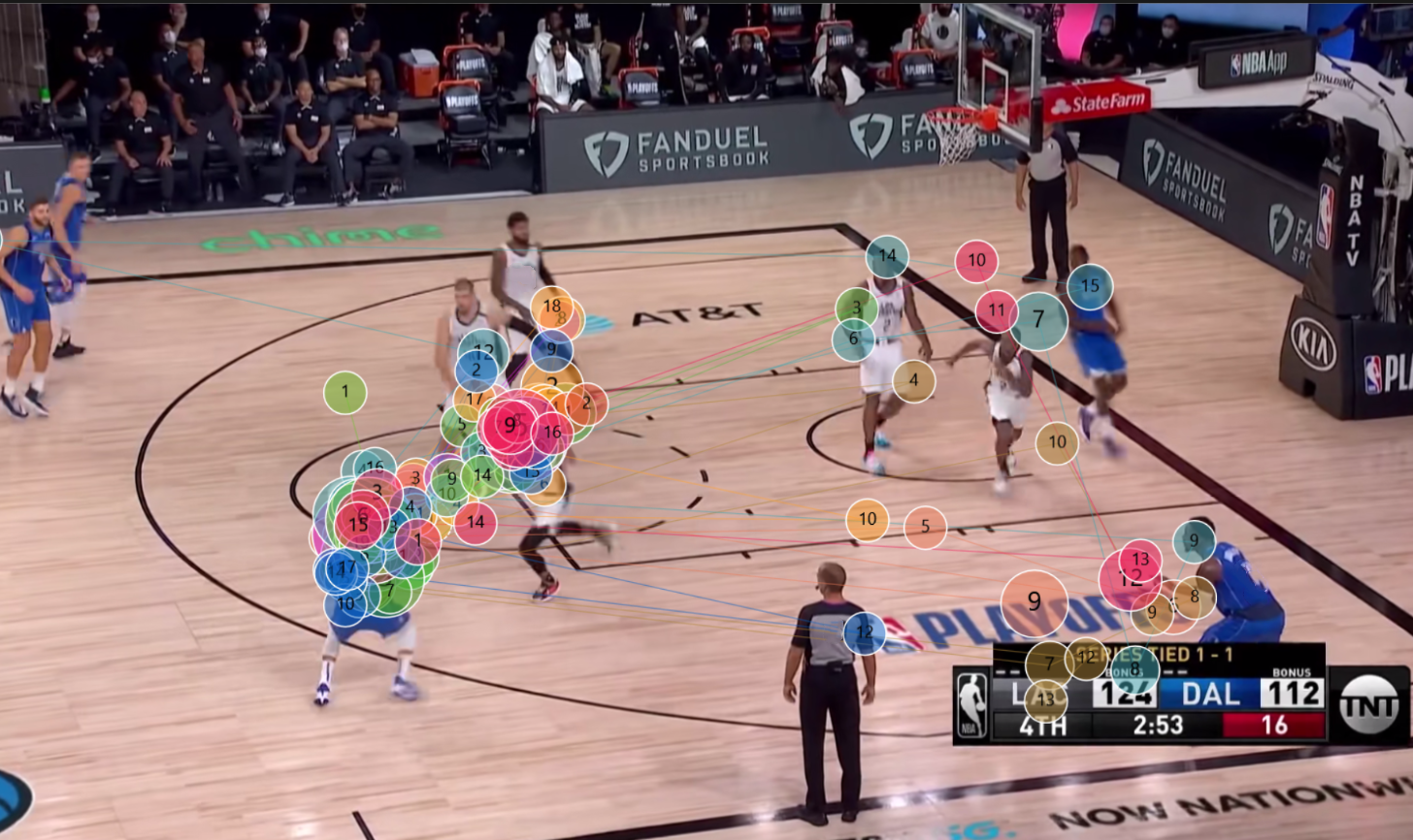

Supplement: Supplementary file 2 — Supplementary Information 2. [file 41598_2023_28754_MOESM2_ESM.zip › Gaze Plot/N5.png]

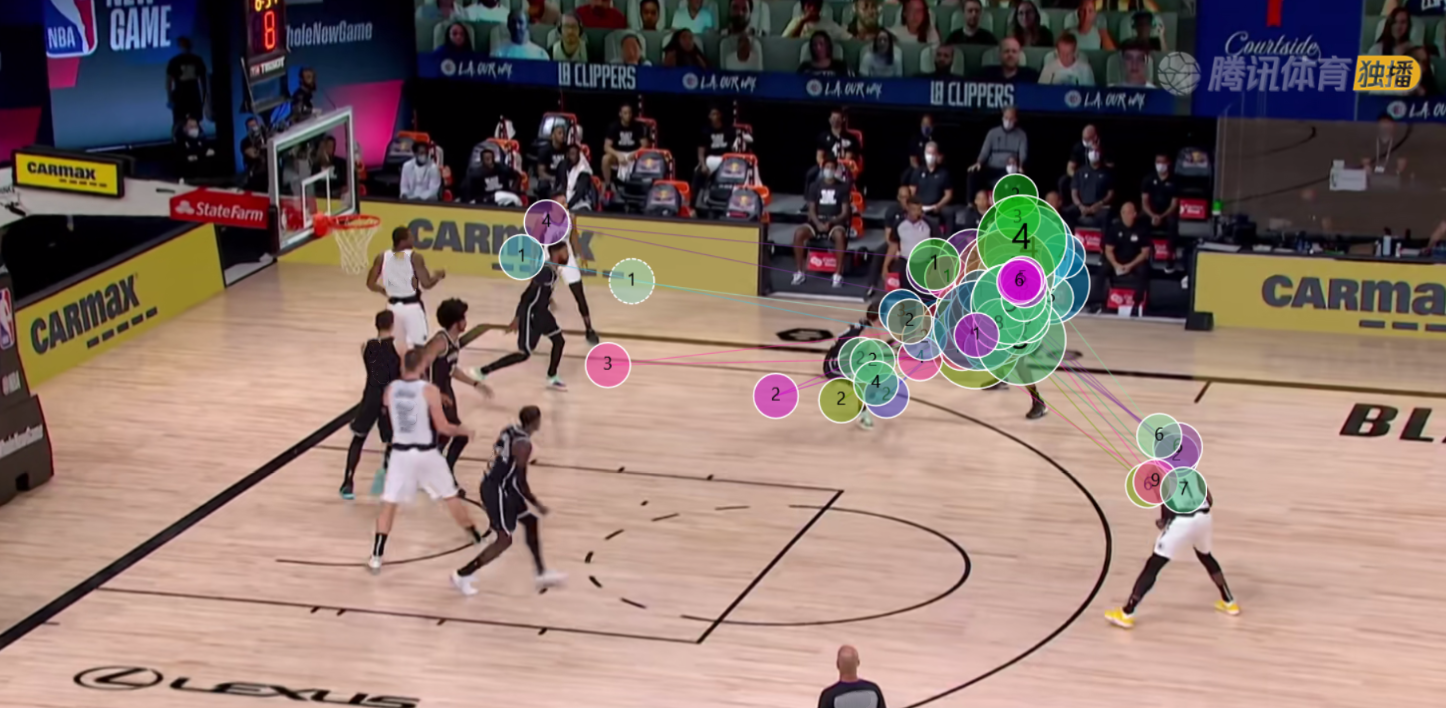

Supplement: Supplementary file 2 — Supplementary Information 2. [file 41598_2023_28754_MOESM2_ESM.zip › Gaze Plot/N6.png]

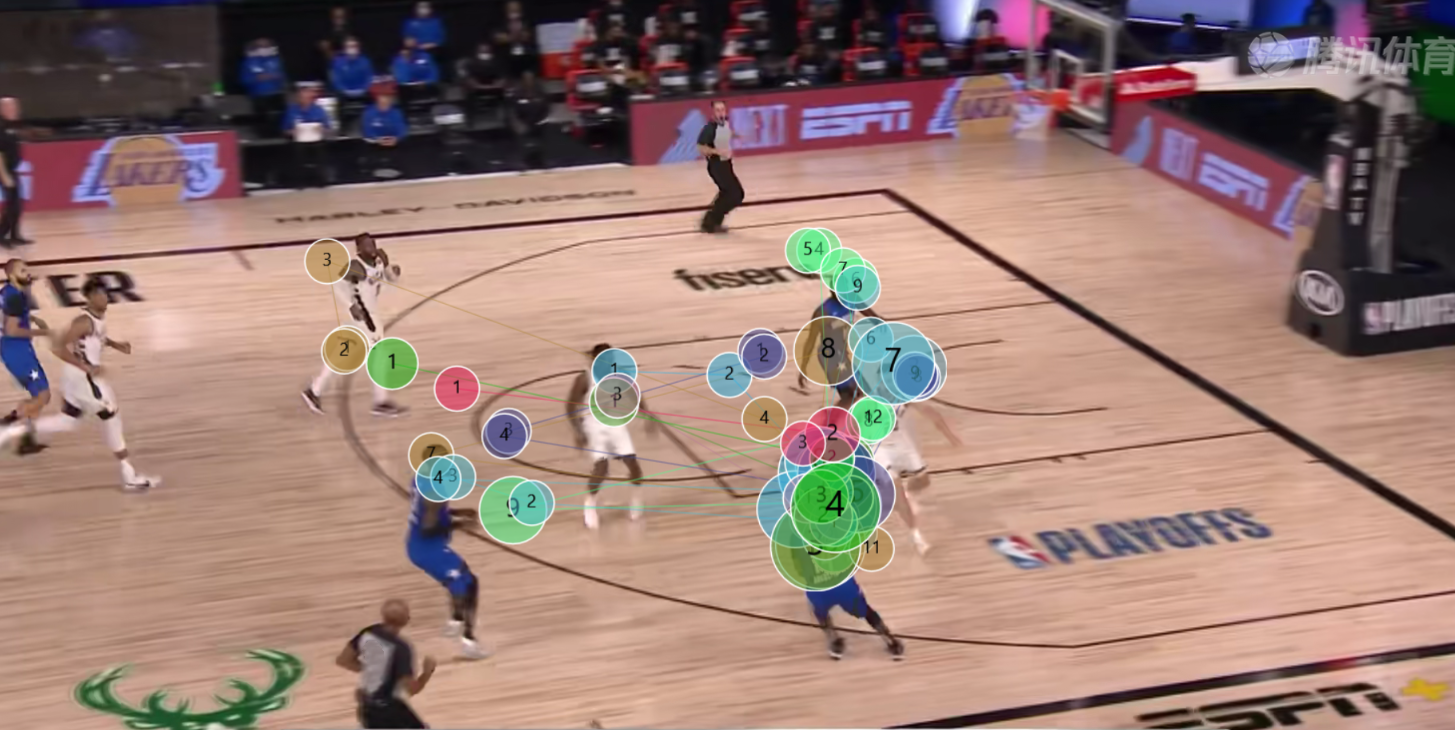

Supplement: Supplementary file 2 — Supplementary Information 2. [file 41598_2023_28754_MOESM2_ESM.zip › Gaze Plot/N7.png]

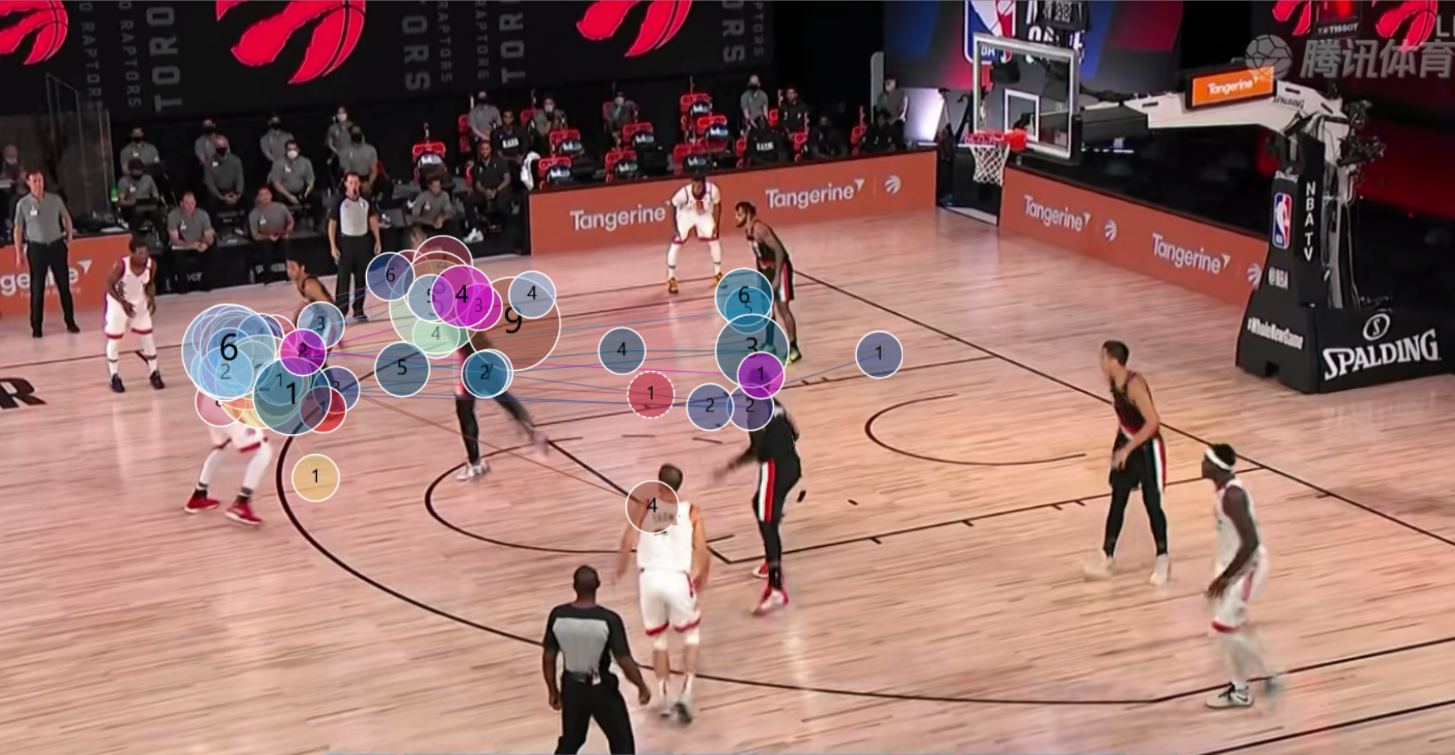

Supplement: Supplementary file 2 — Supplementary Information 2. [file 41598_2023_28754_MOESM2_ESM.zip › Gaze Plot/N8.png]

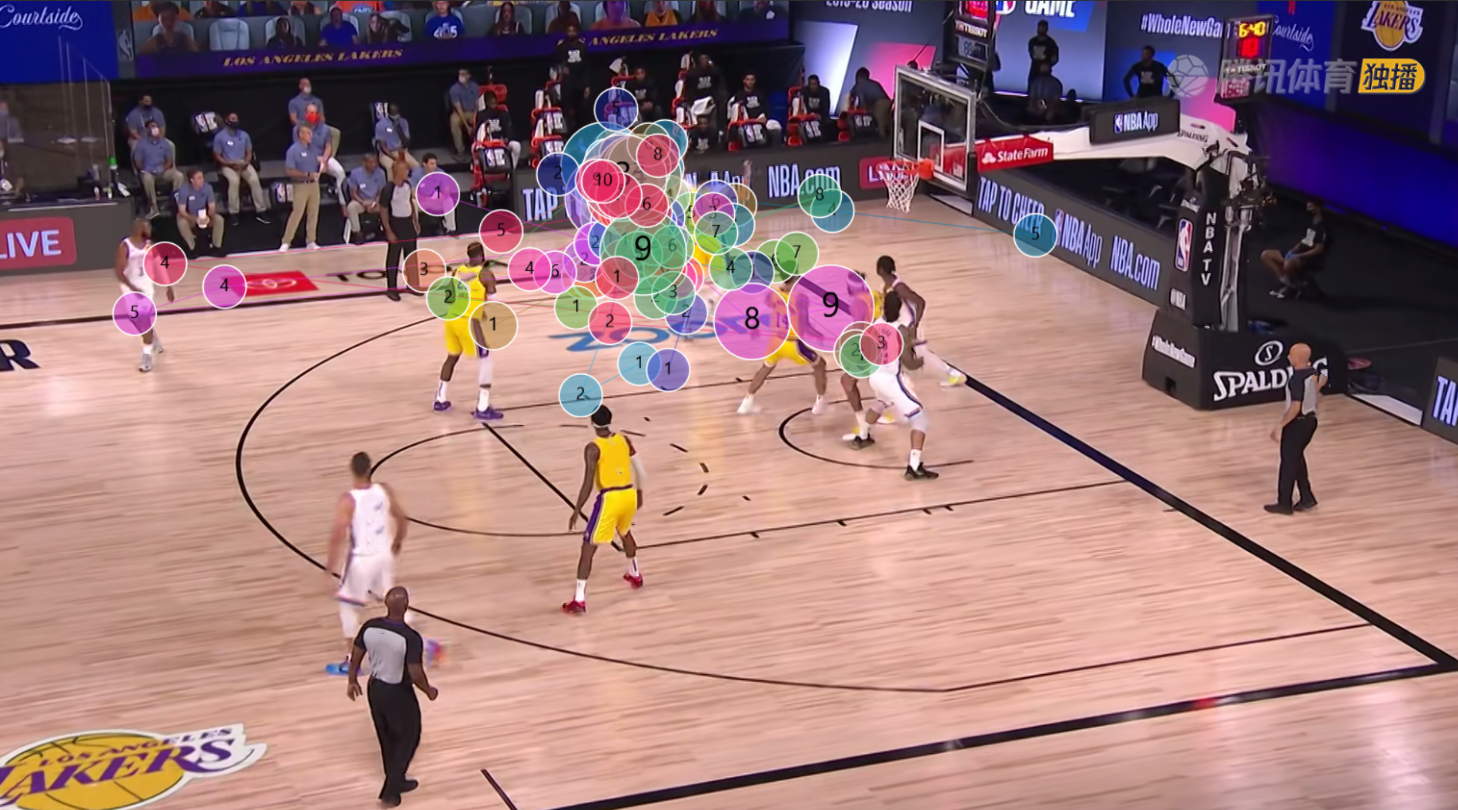

Supplement: Supplementary file 2 — Supplementary Information 2. [file 41598_2023_28754_MOESM2_ESM.zip › Gaze Plot/N9.png]

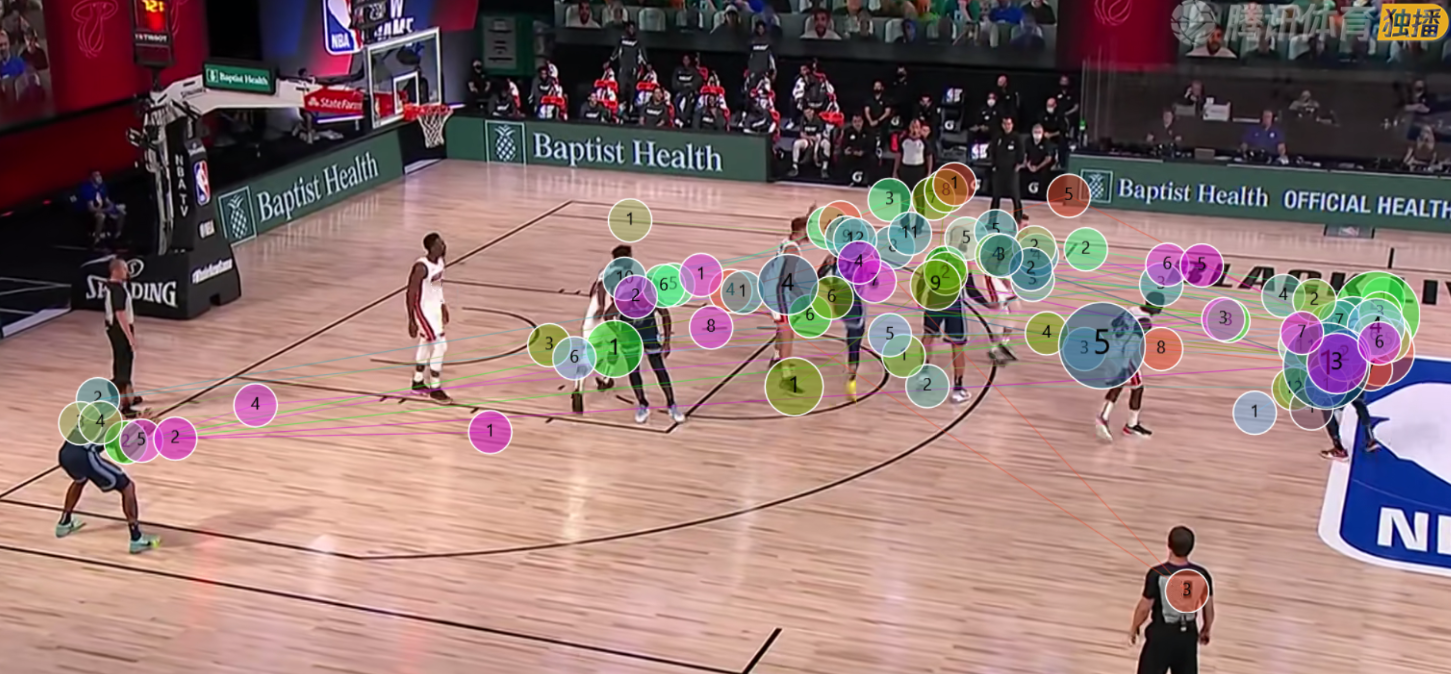

Supplement: Supplementary file 2 — Supplementary Information 2. [file 41598_2023_28754_MOESM2_ESM.zip › Gaze Plot/Novice1.png]

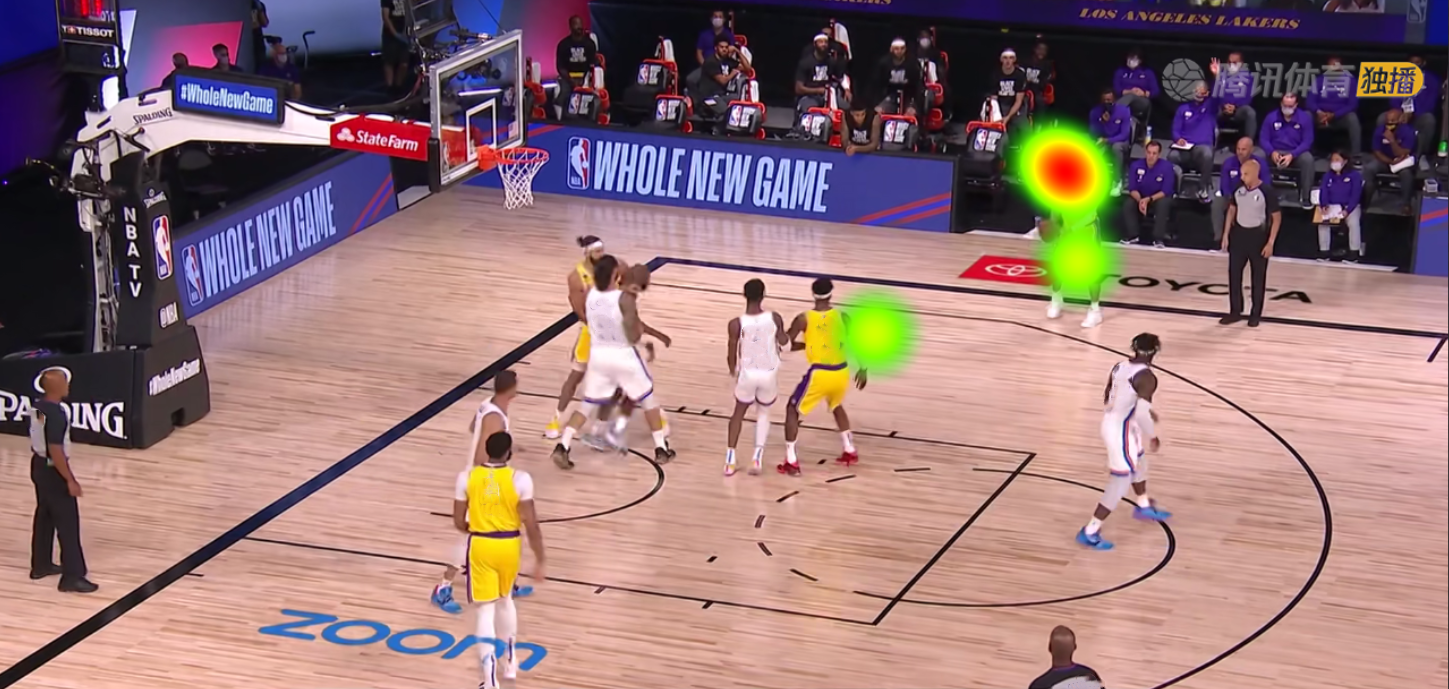

Supplement: Supplementary file 3 — Supplementary Information 3. [file 41598_2023_28754_MOESM3_ESM.zip › Heat map/E10.png]

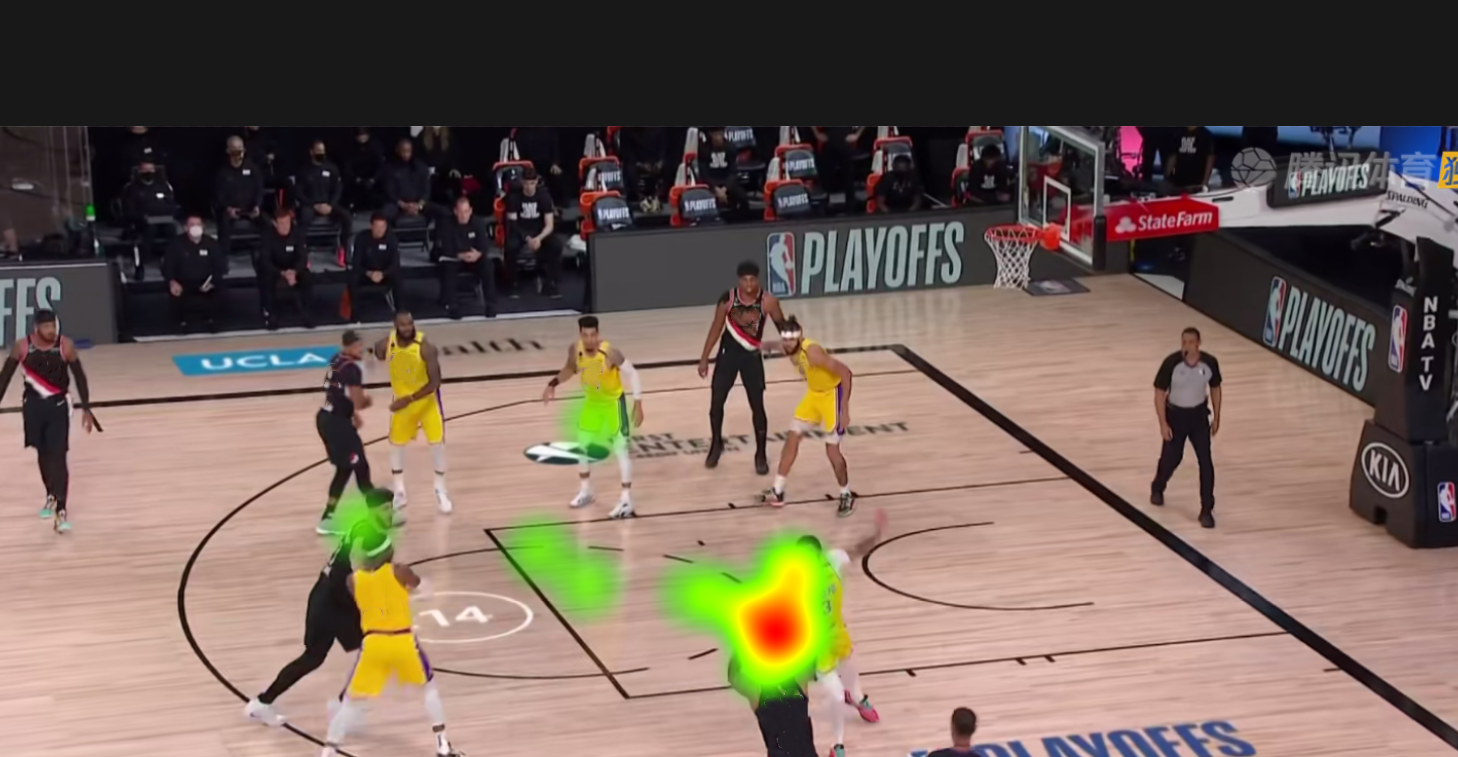

Supplement: Supplementary file 3 — Supplementary Information 3. [file 41598_2023_28754_MOESM3_ESM.zip › Heat map/E11.png]

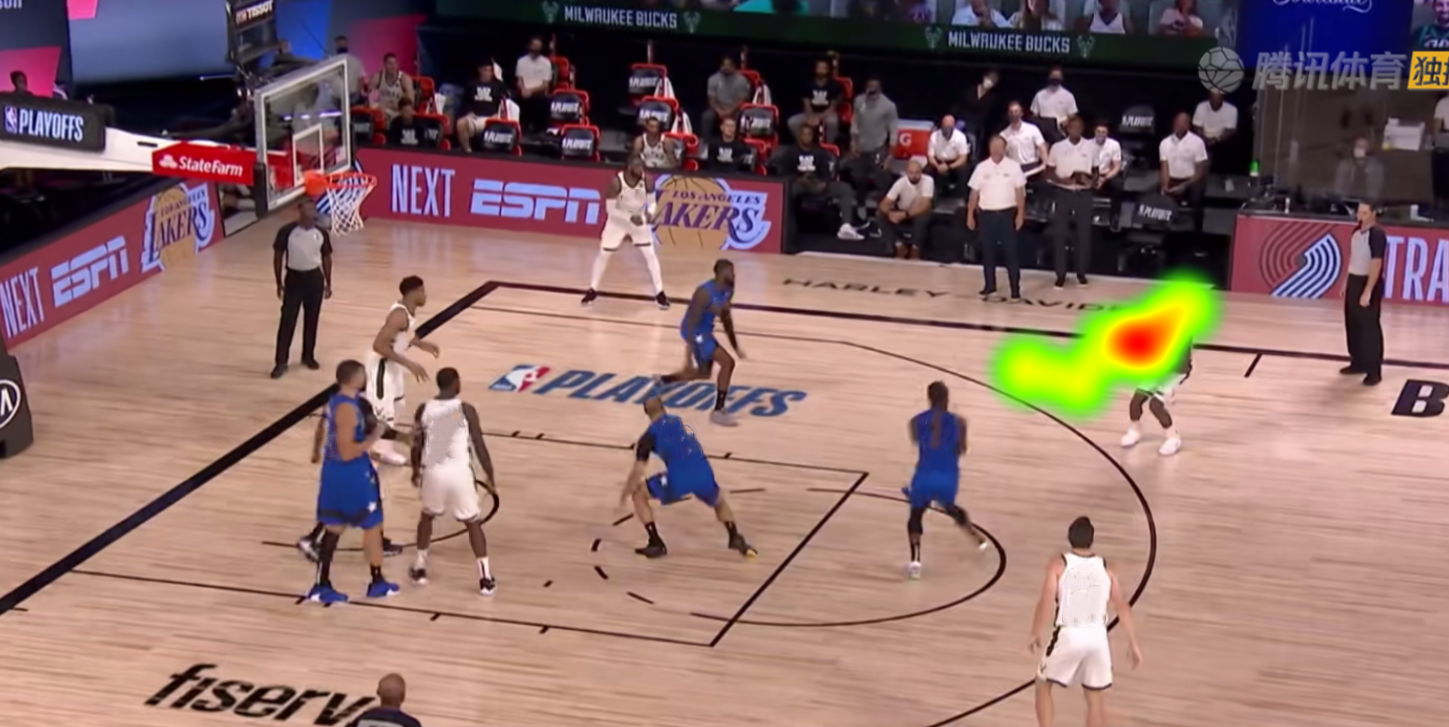

Supplement: Supplementary file 3 — Supplementary Information 3. [file 41598_2023_28754_MOESM3_ESM.zip › Heat map/E12.png]

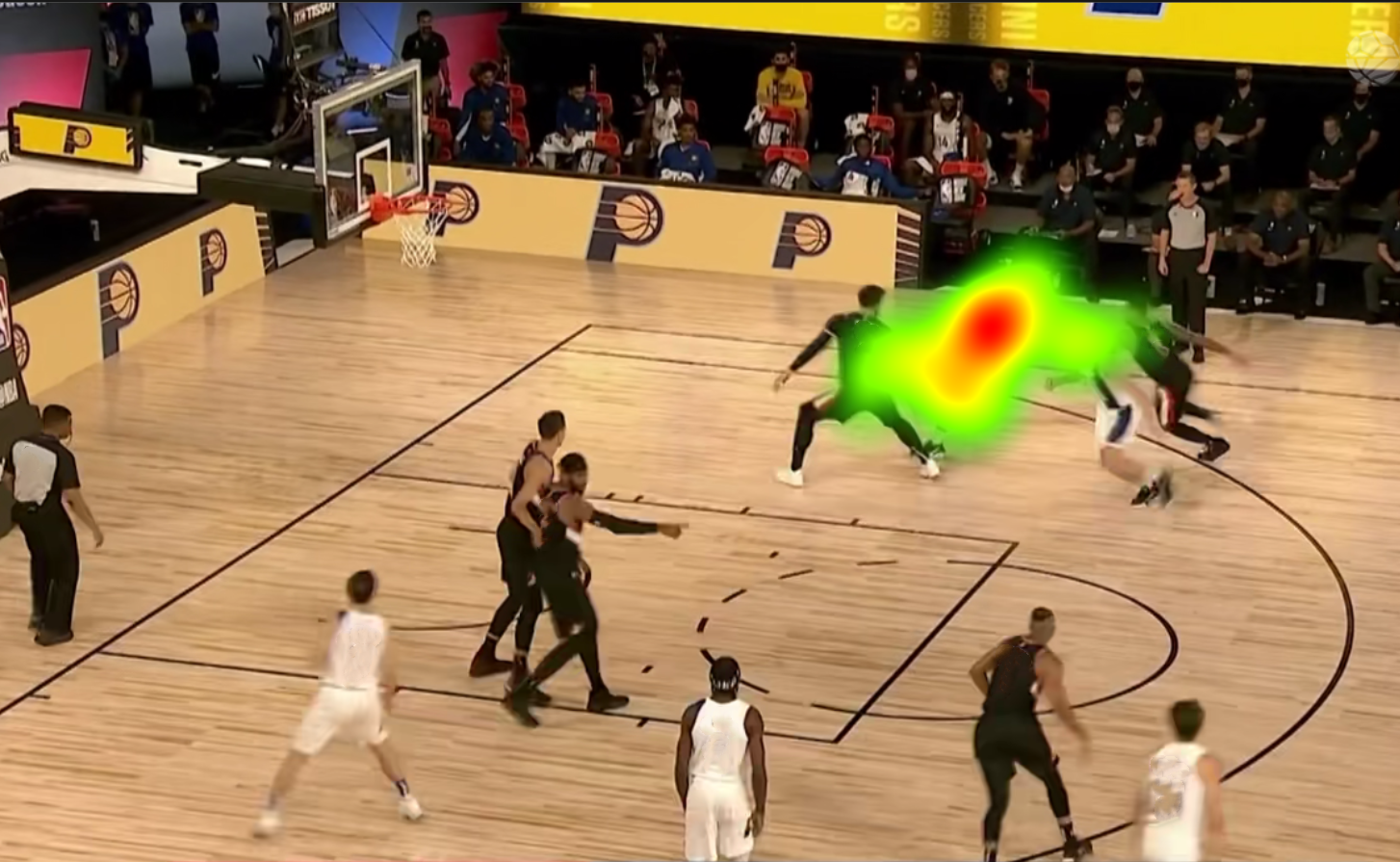

Supplement: Supplementary file 3 — Supplementary Information 3. [file 41598_2023_28754_MOESM3_ESM.zip › Heat map/E13.png]

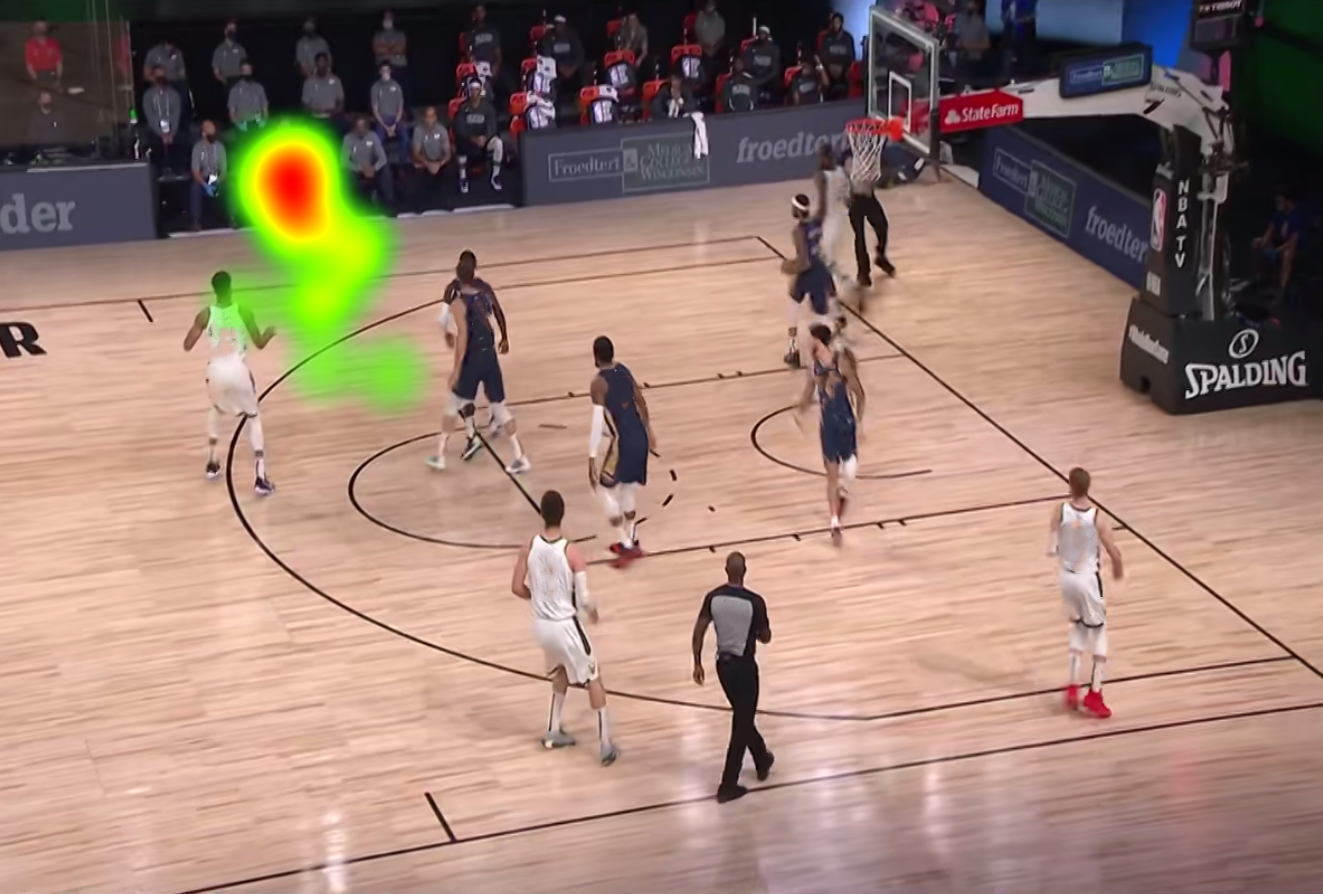

Supplement: Supplementary file 3 — Supplementary Information 3. [file 41598_2023_28754_MOESM3_ESM.zip › Heat map/E14.png]

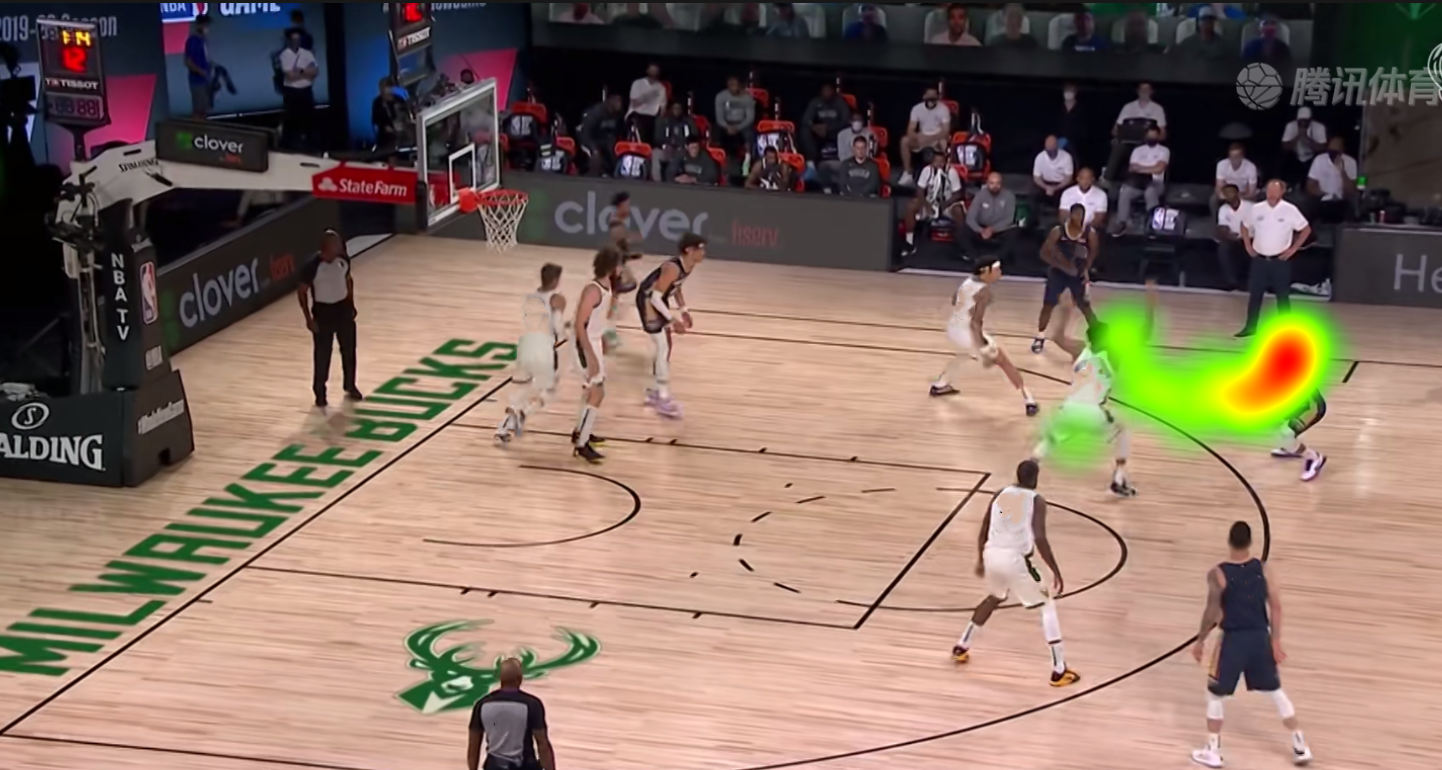

Supplement: Supplementary file 3 — Supplementary Information 3. [file 41598_2023_28754_MOESM3_ESM.zip › Heat map/E15.png]

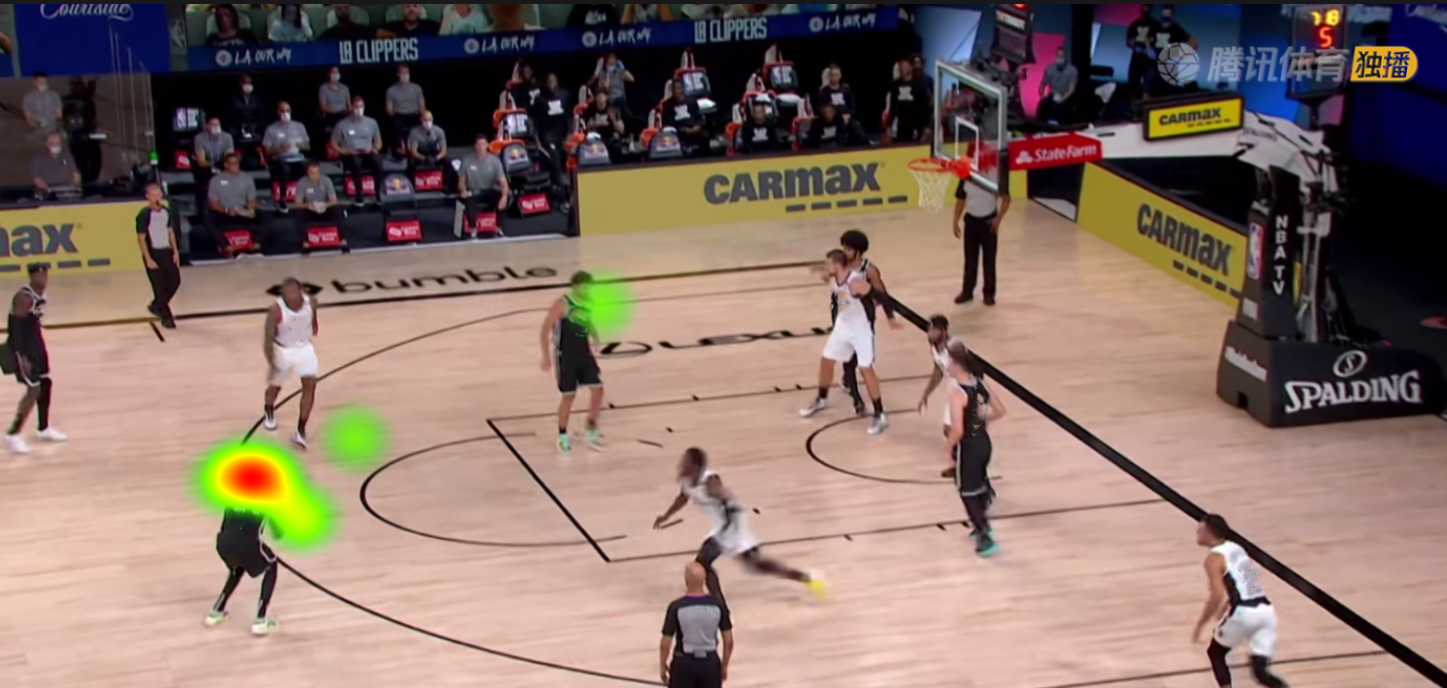

Supplement: Supplementary file 3 — Supplementary Information 3. [file 41598_2023_28754_MOESM3_ESM.zip › Heat map/E16.png]

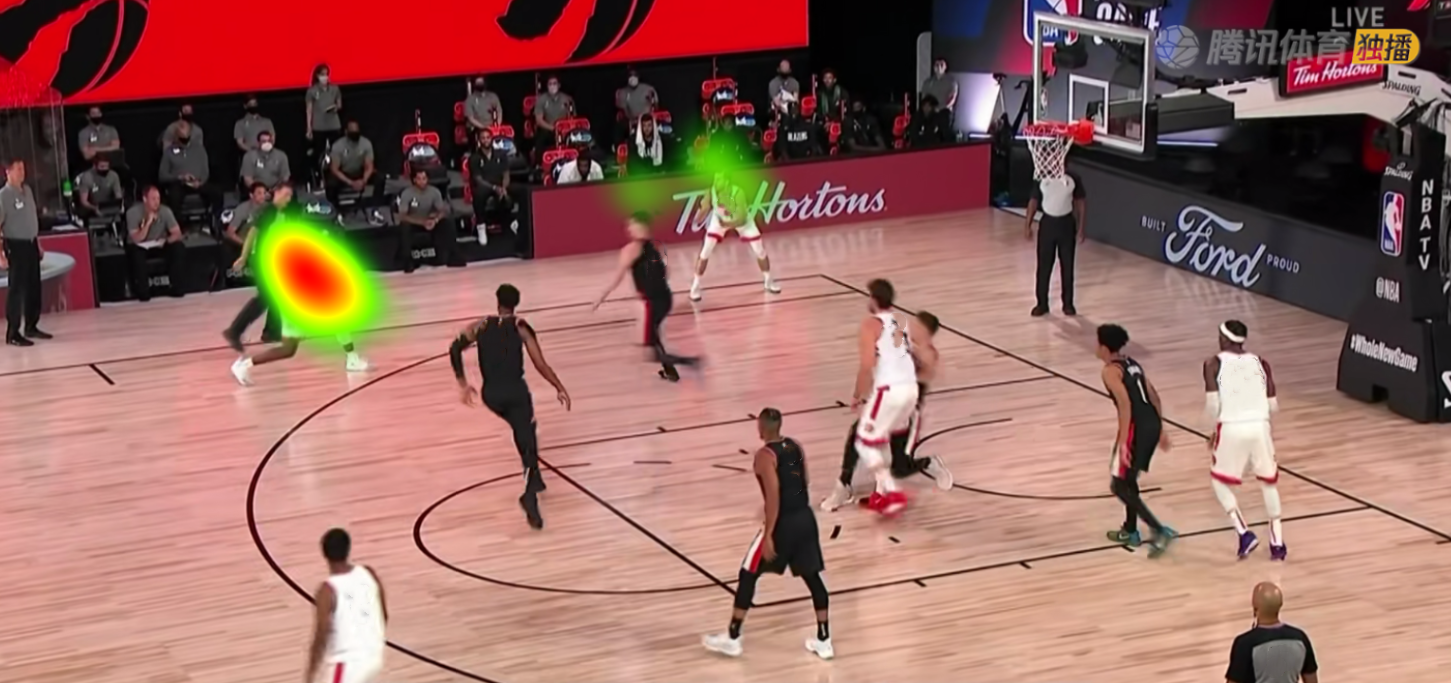

Supplement: Supplementary file 3 — Supplementary Information 3. [file 41598_2023_28754_MOESM3_ESM.zip › Heat map/E17.png]

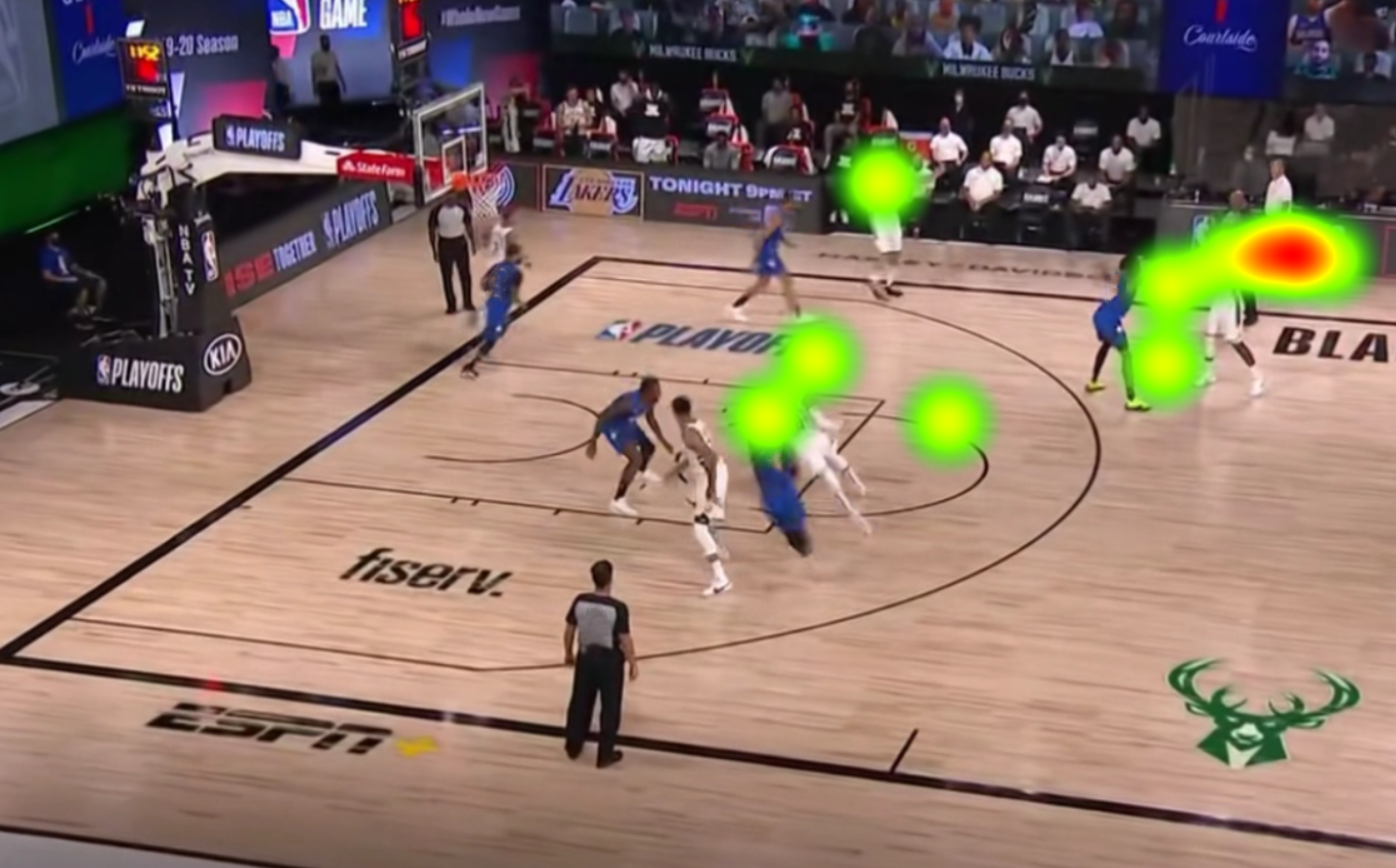

Supplement: Supplementary file 3 — Supplementary Information 3. [file 41598_2023_28754_MOESM3_ESM.zip › Heat map/E18.png]

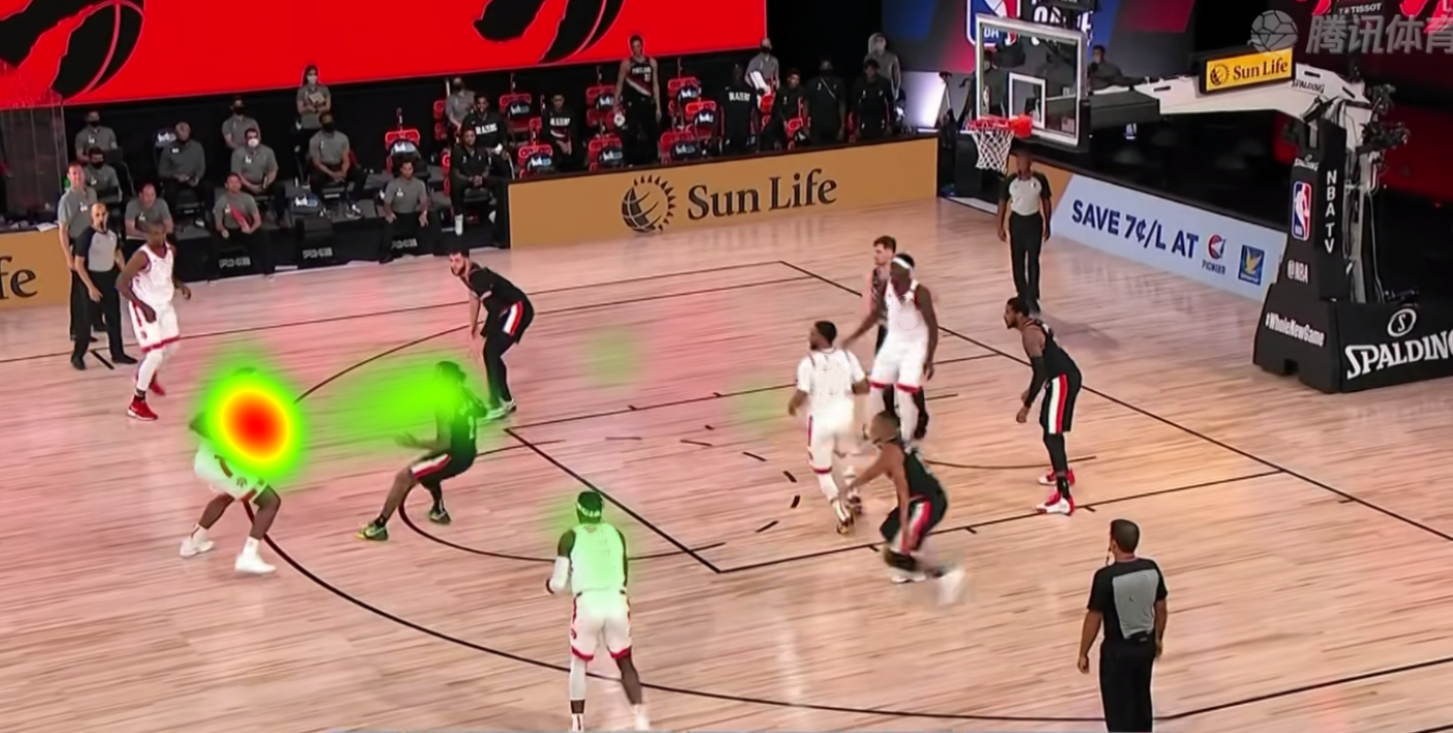

Supplement: Supplementary file 3 — Supplementary Information 3. [file 41598_2023_28754_MOESM3_ESM.zip › Heat map/E19.png]

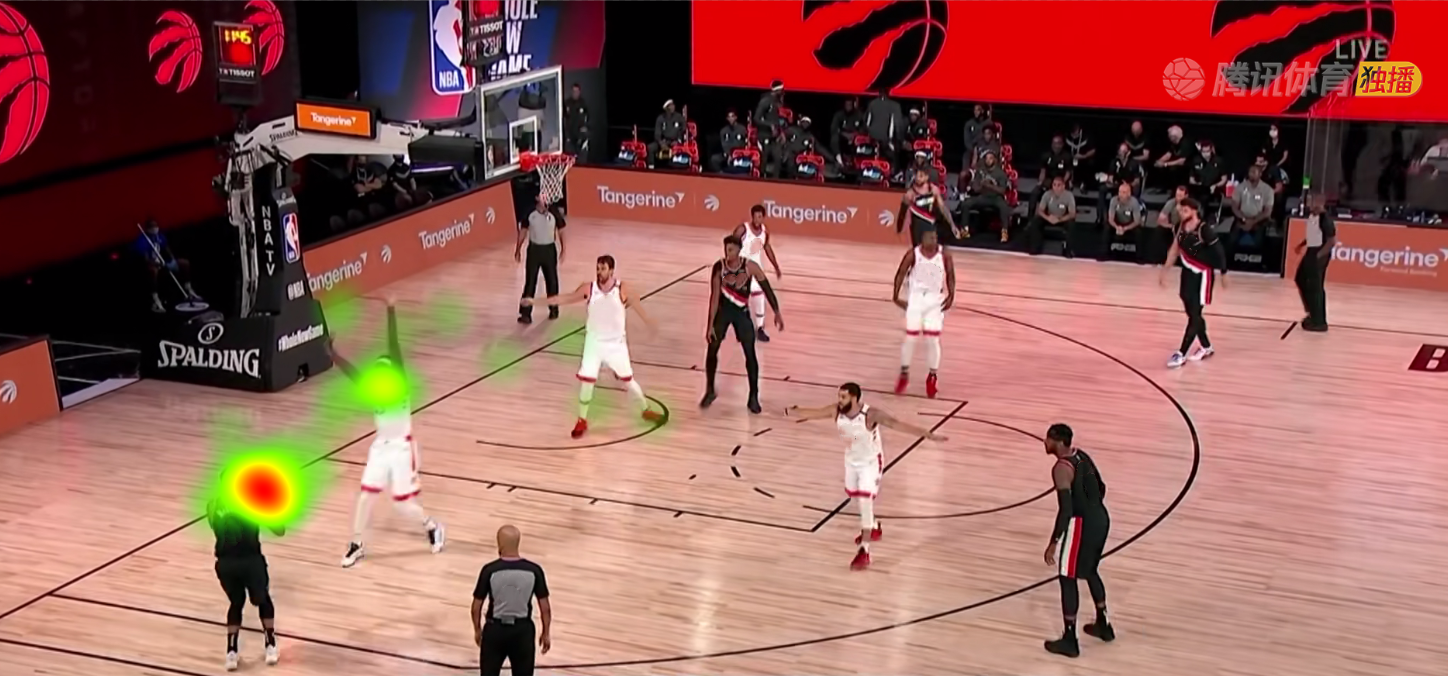

Supplement: Supplementary file 3 — Supplementary Information 3. [file 41598_2023_28754_MOESM3_ESM.zip › Heat map/E2.png]

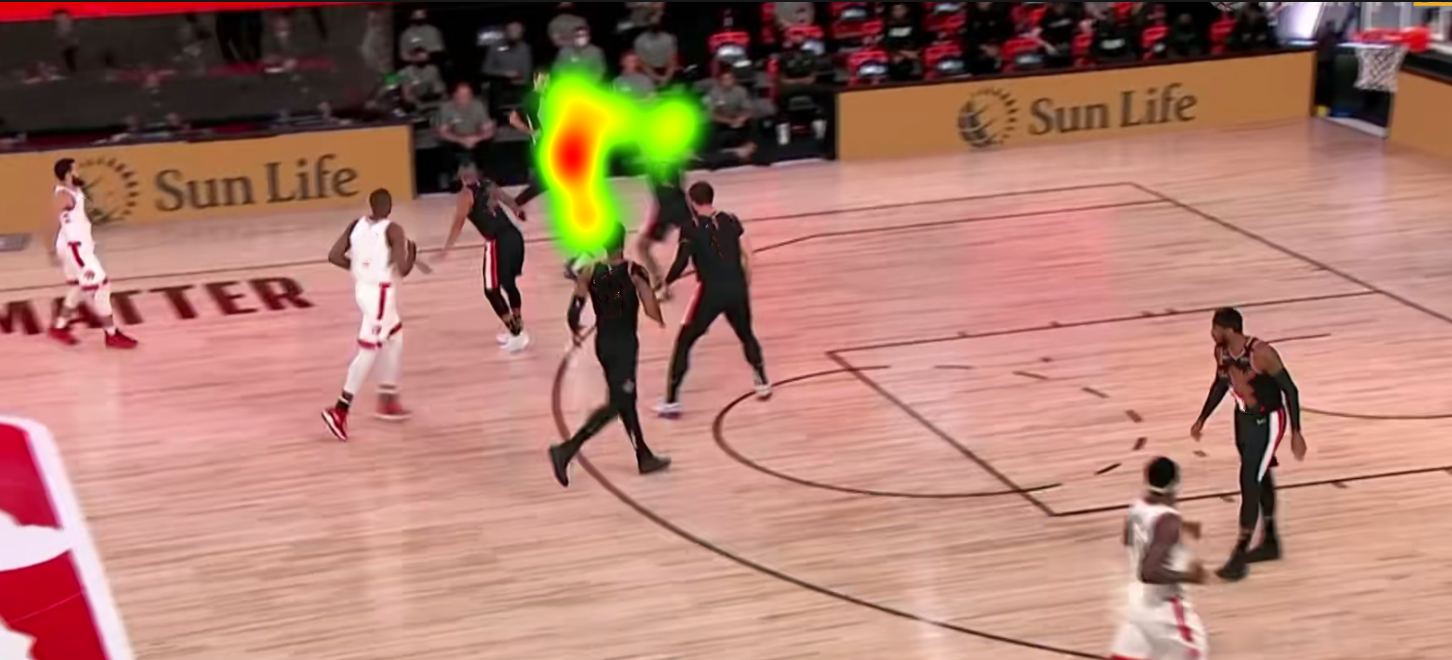

Supplement: Supplementary file 3 — Supplementary Information 3. [file 41598_2023_28754_MOESM3_ESM.zip › Heat map/E20.png]

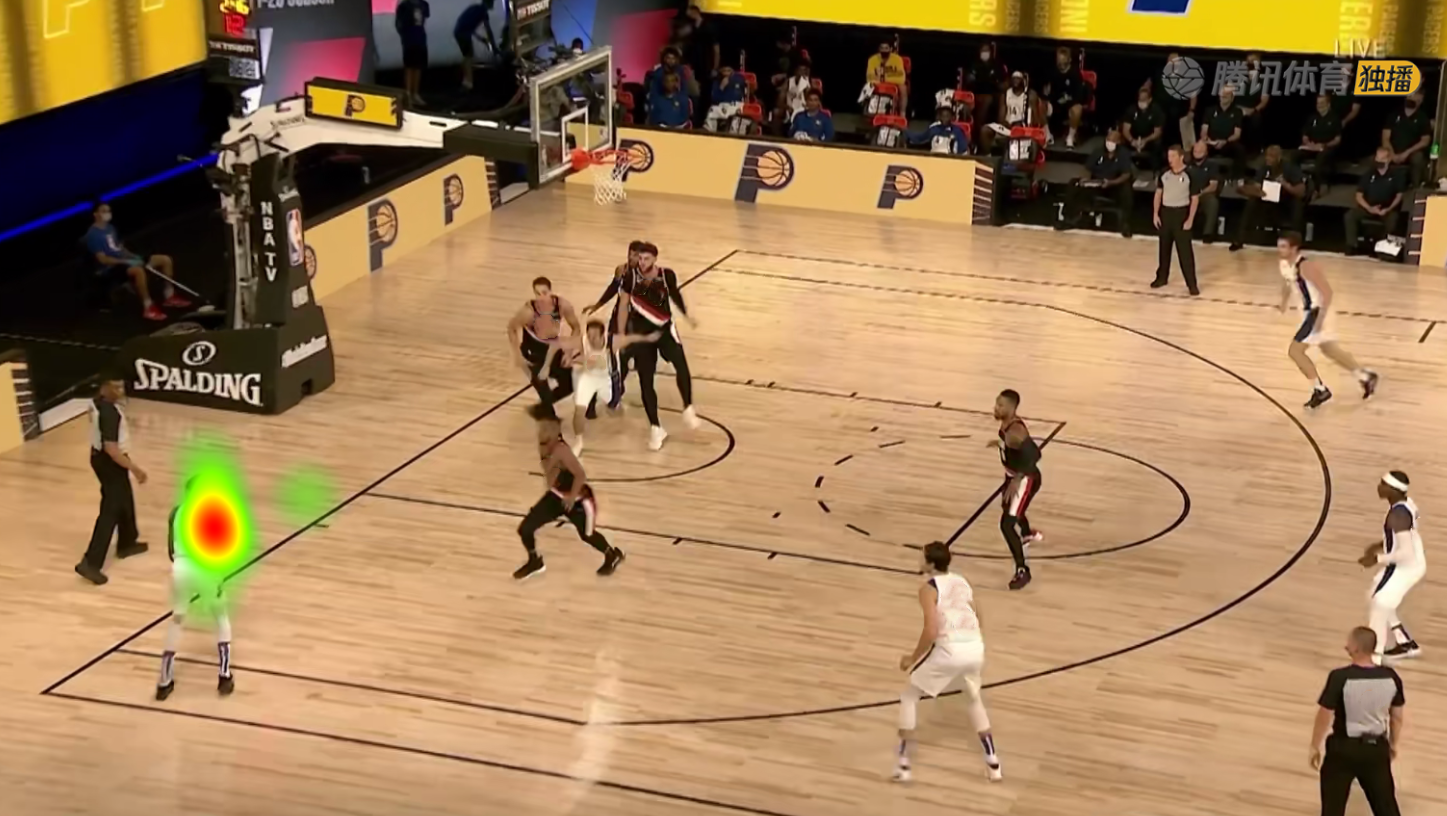

Supplement: Supplementary file 3 — Supplementary Information 3. [file 41598_2023_28754_MOESM3_ESM.zip › Heat map/E21.png]

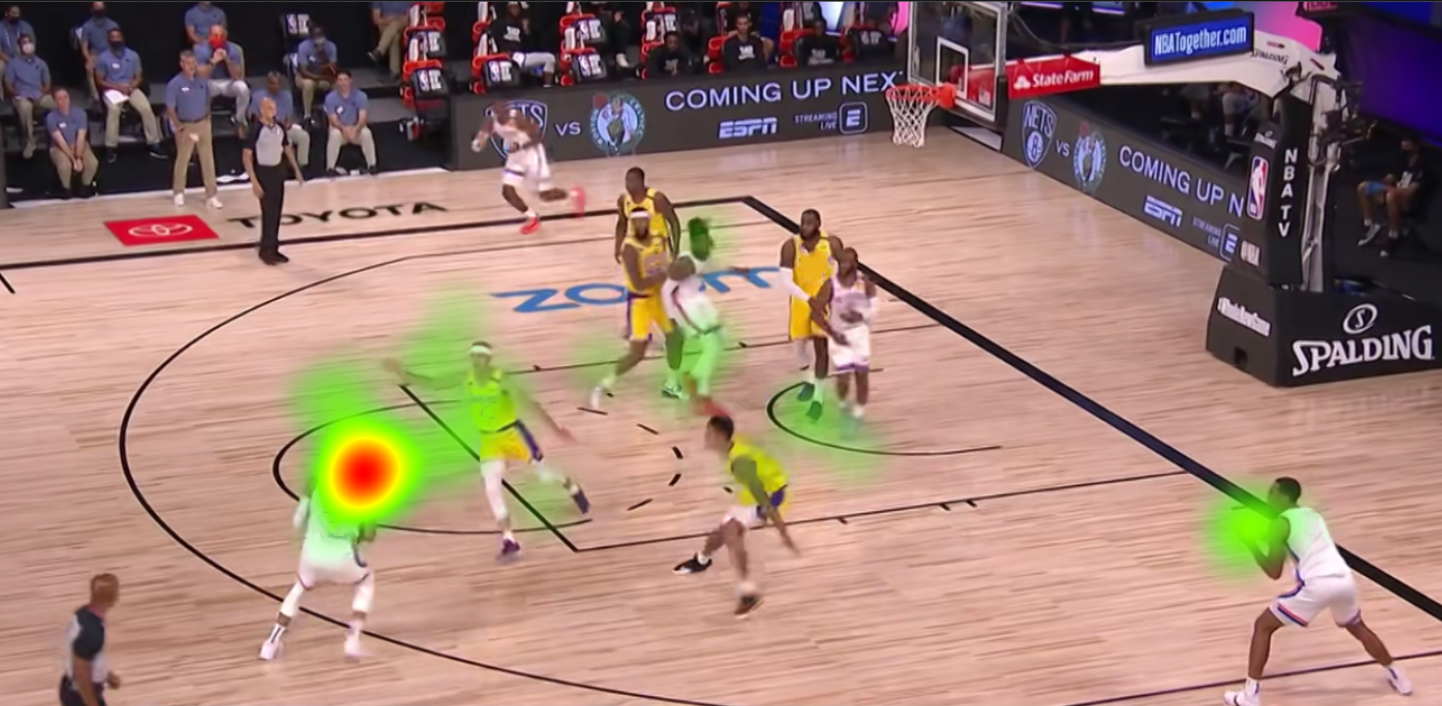

Supplement: Supplementary file 3 — Supplementary Information 3. [file 41598_2023_28754_MOESM3_ESM.zip › Heat map/E3.png]

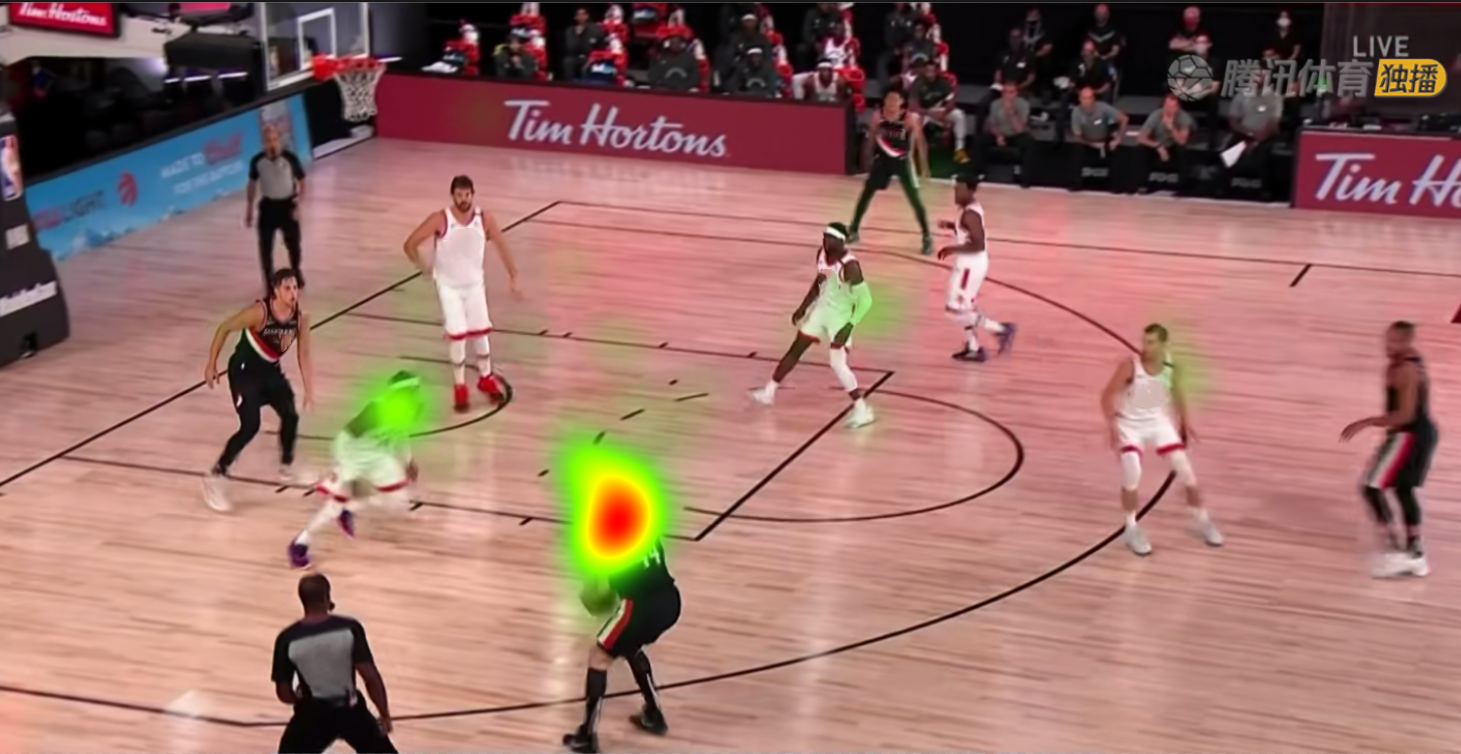

Supplement: Supplementary file 3 — Supplementary Information 3. [file 41598_2023_28754_MOESM3_ESM.zip › Heat map/E4.png]

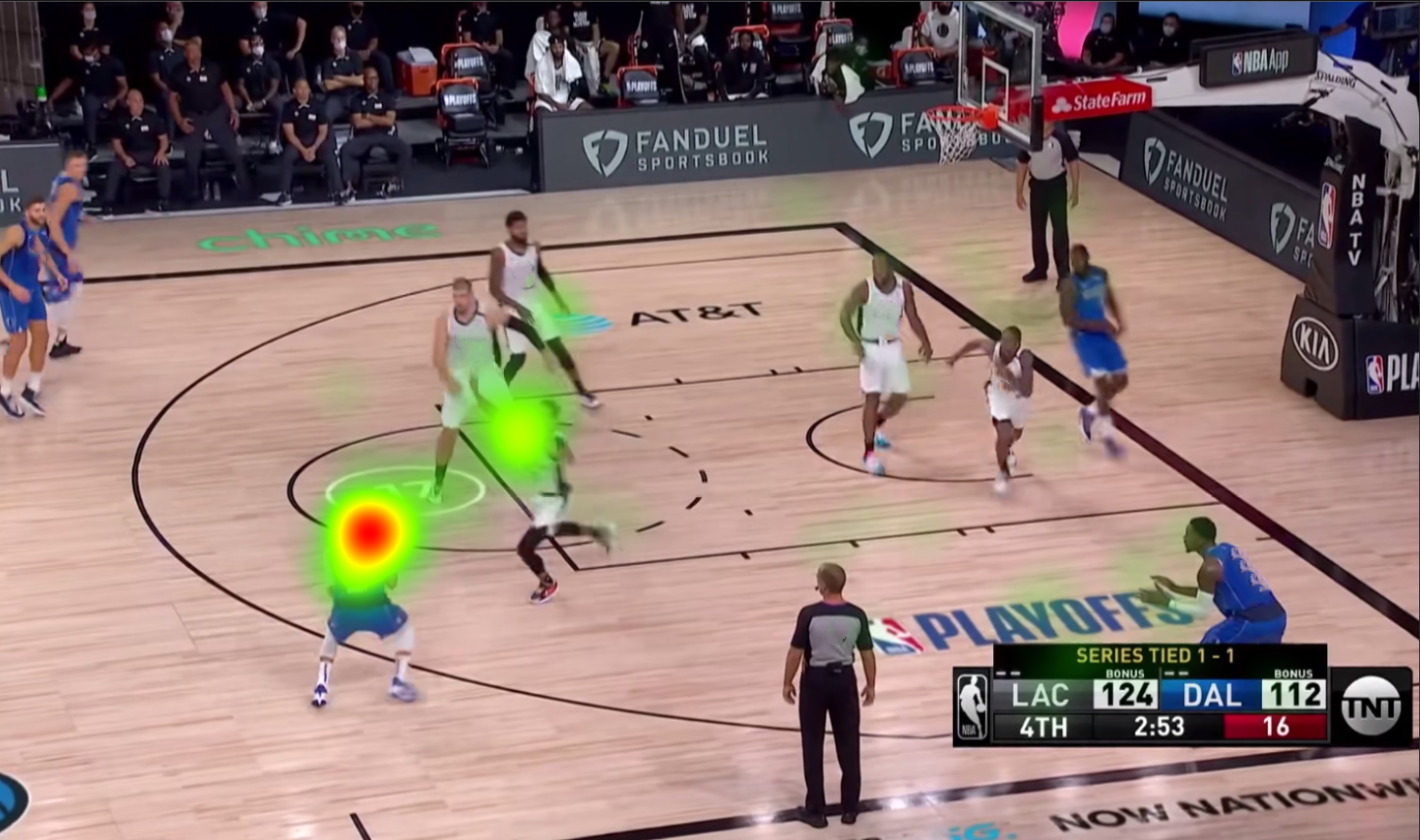

Supplement: Supplementary file 3 — Supplementary Information 3. [file 41598_2023_28754_MOESM3_ESM.zip › Heat map/E5.png]

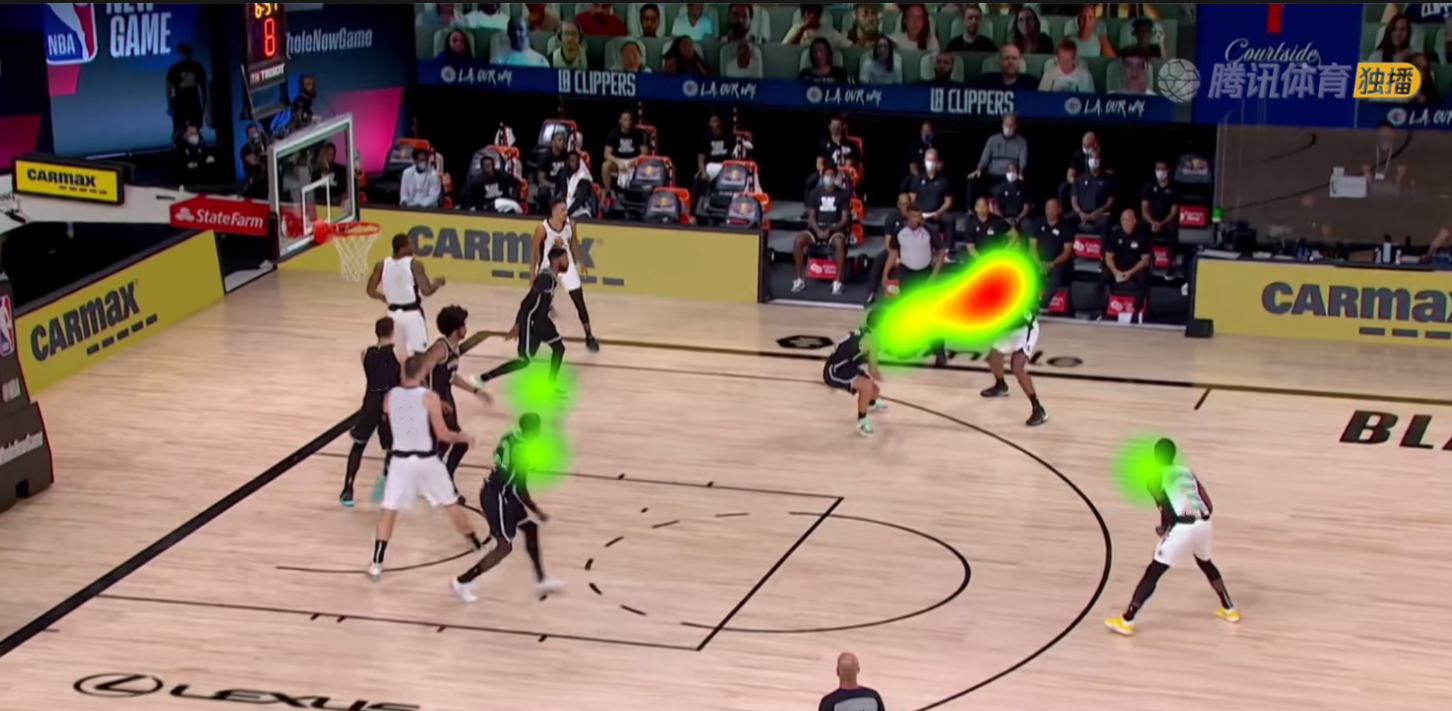

Supplement: Supplementary file 3 — Supplementary Information 3. [file 41598_2023_28754_MOESM3_ESM.zip › Heat map/E6.png]

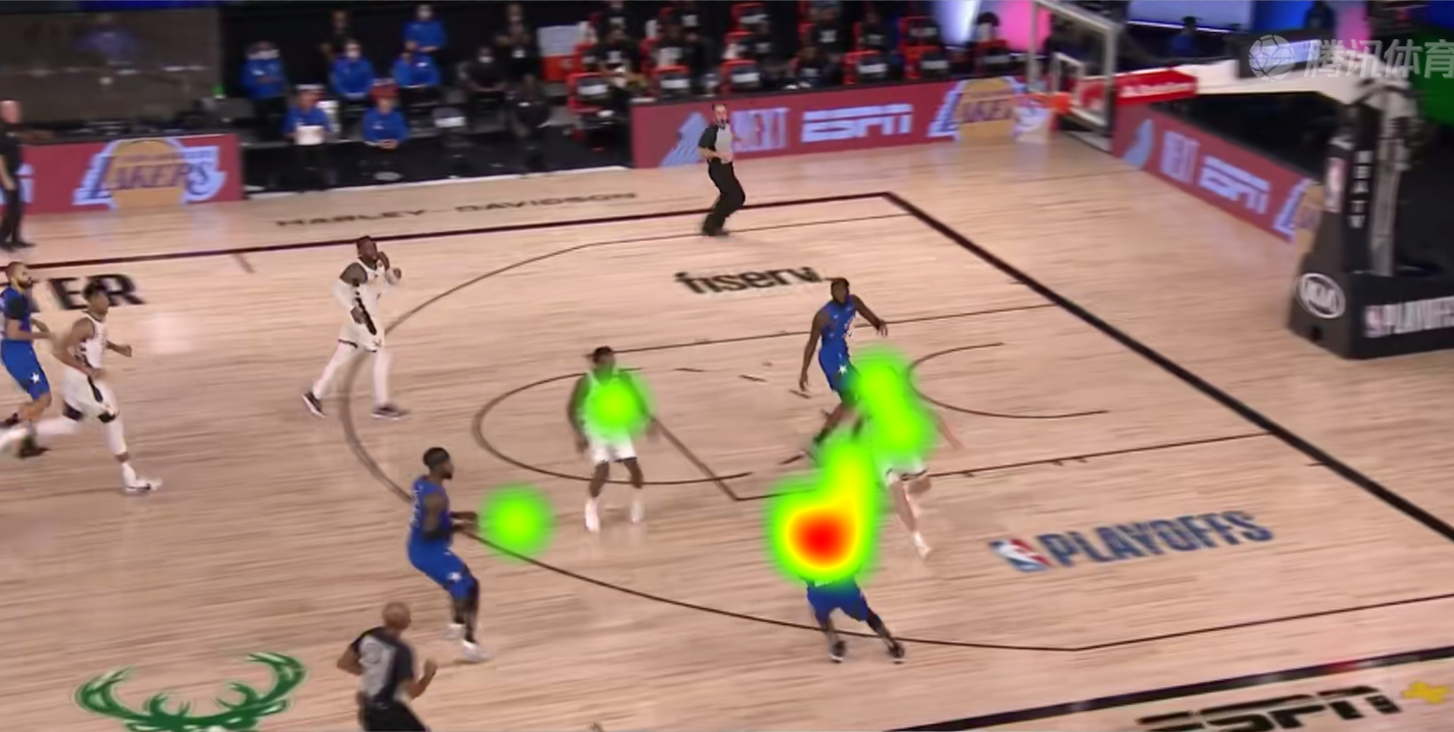

Supplement: Supplementary file 3 — Supplementary Information 3. [file 41598_2023_28754_MOESM3_ESM.zip › Heat map/E7.png]

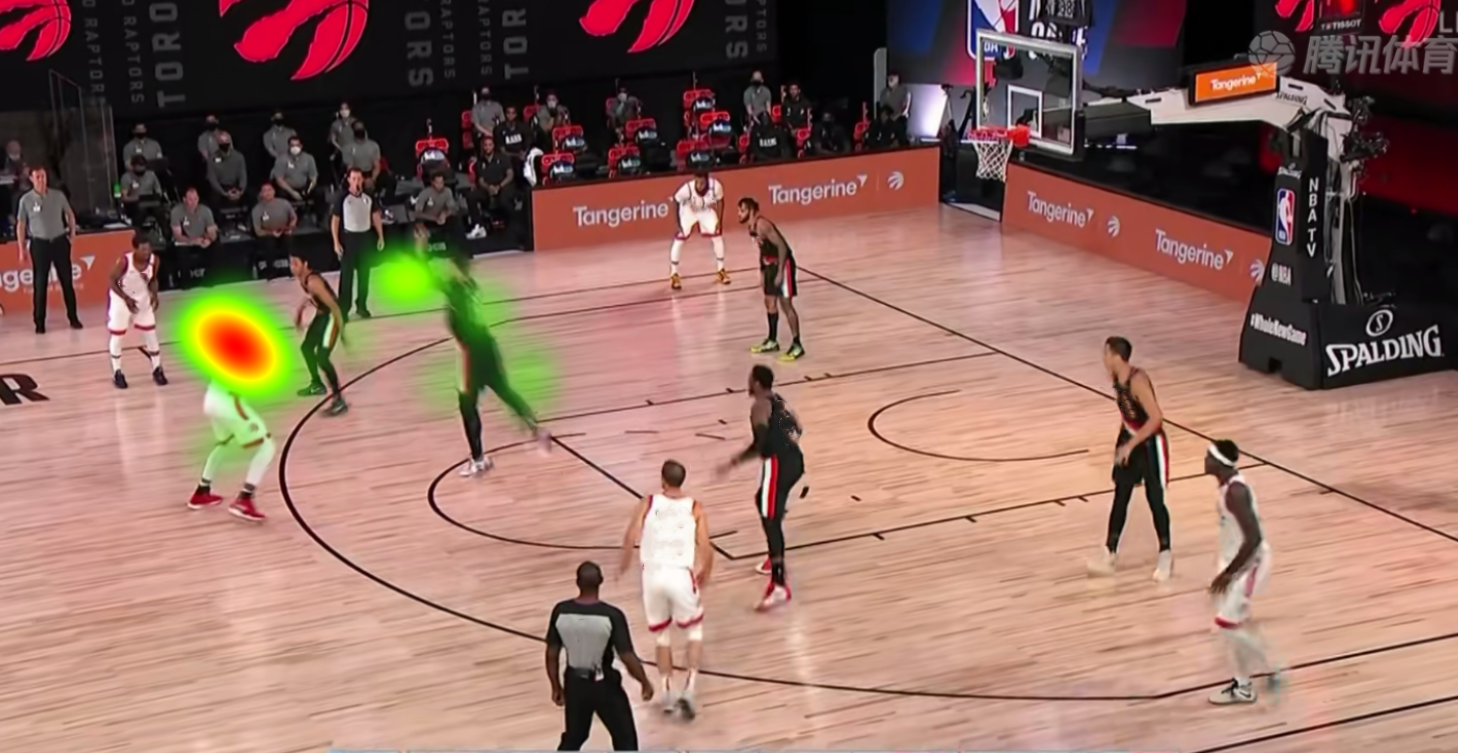

Supplement: Supplementary file 3 — Supplementary Information 3. [file 41598_2023_28754_MOESM3_ESM.zip › Heat map/E8.png]

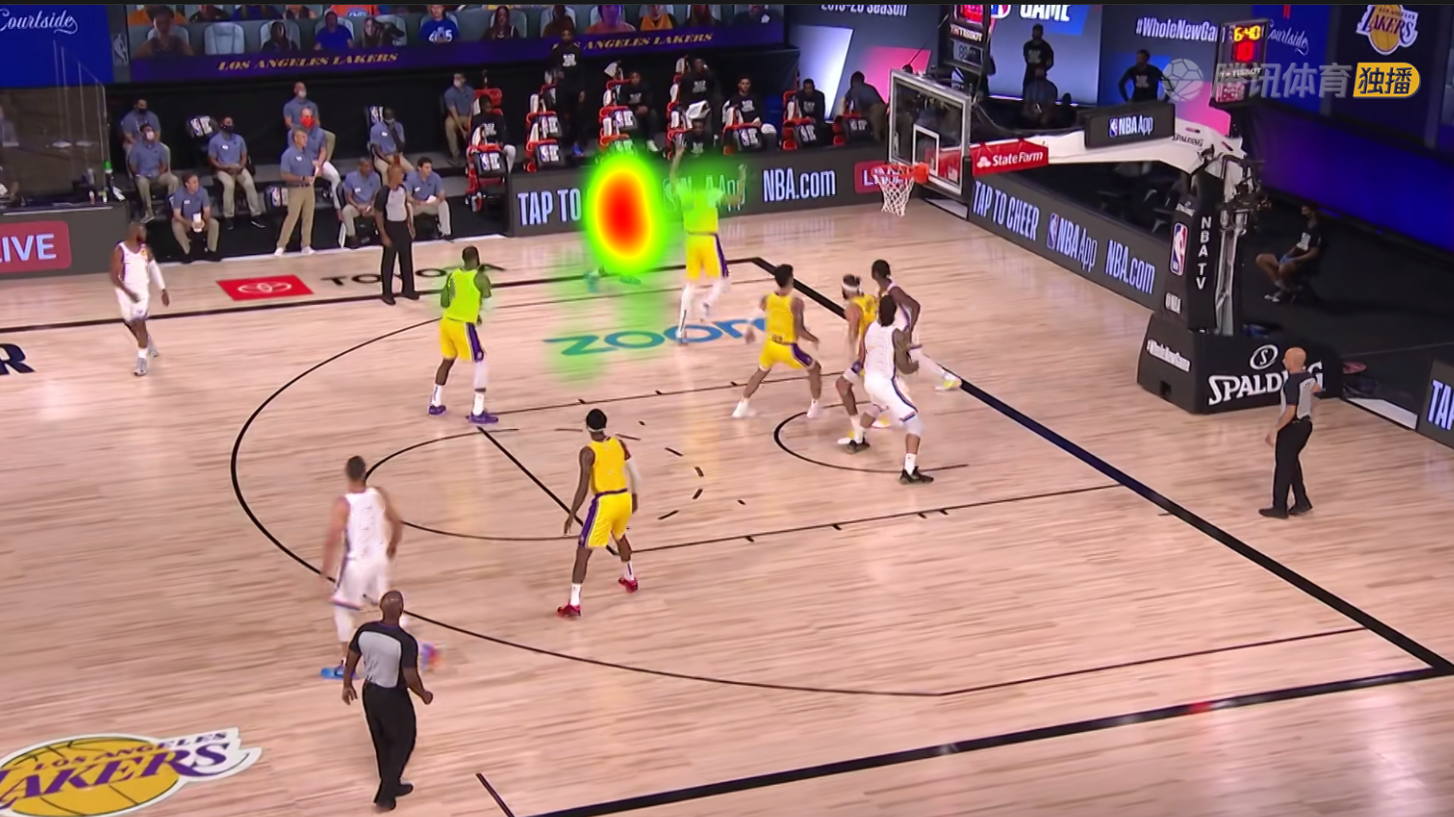

Supplement: Supplementary file 3 — Supplementary Information 3. [file 41598_2023_28754_MOESM3_ESM.zip › Heat map/E9.png]

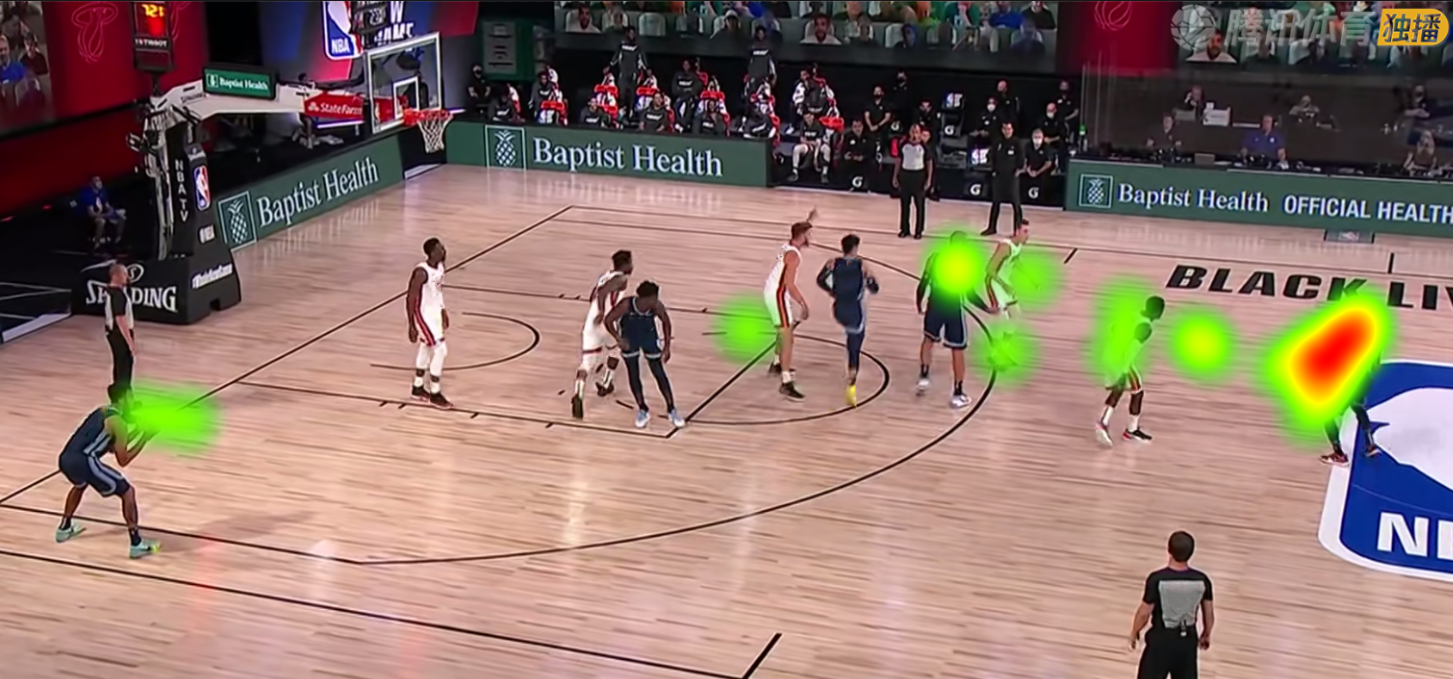

Supplement: Supplementary file 3 — Supplementary Information 3. [file 41598_2023_28754_MOESM3_ESM.zip › Heat map/Expert 1.png]

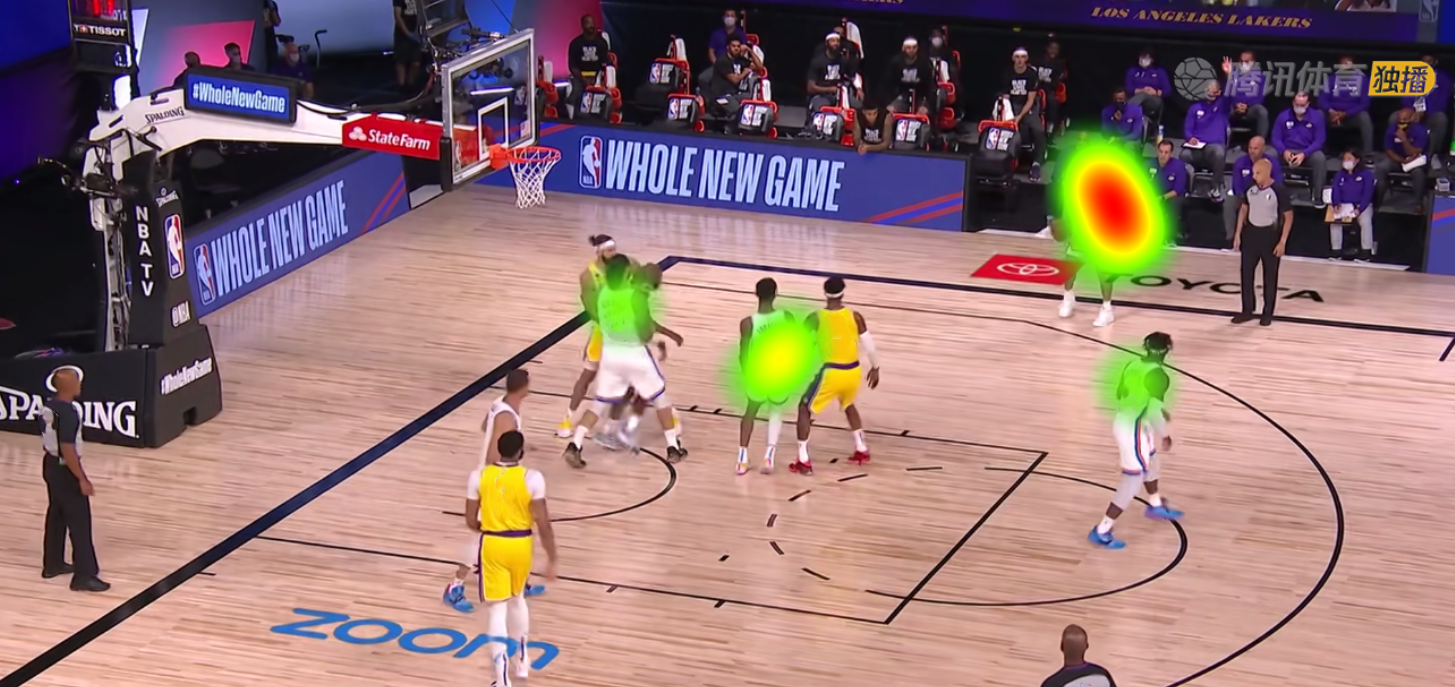

Supplement: Supplementary file 3 — Supplementary Information 3. [file 41598_2023_28754_MOESM3_ESM.zip › Heat map/N10.png]

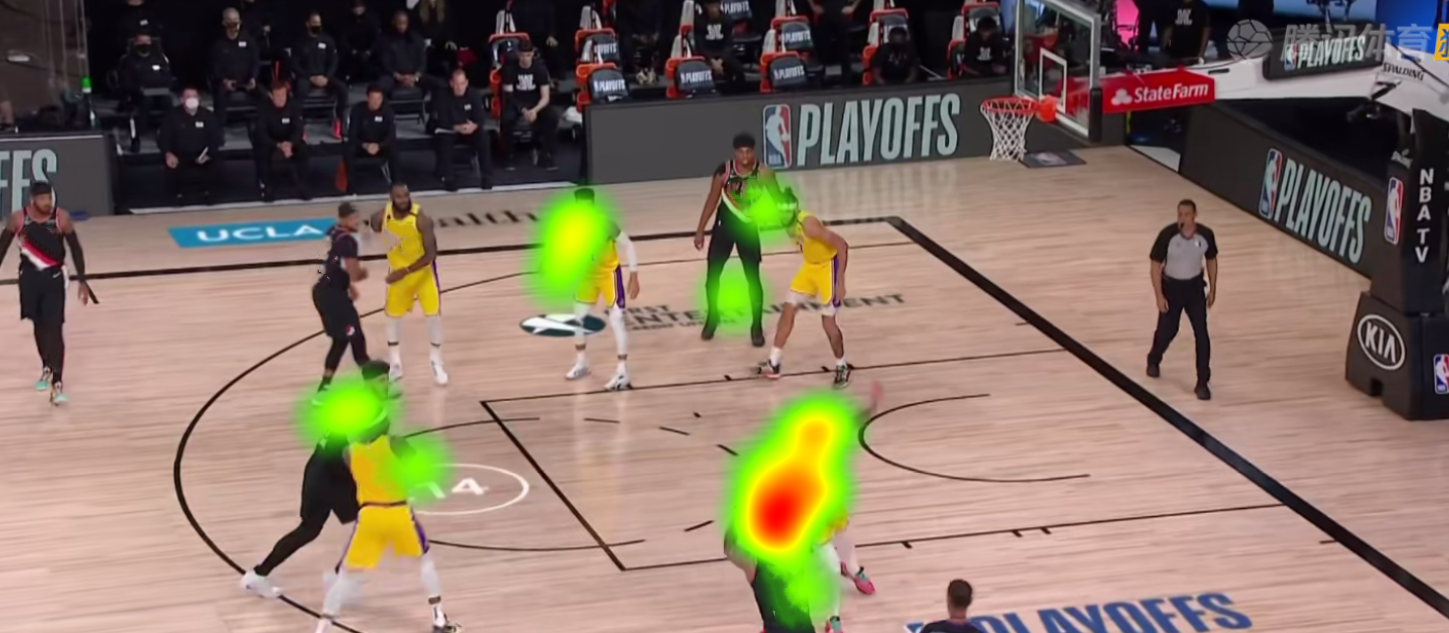

Supplement: Supplementary file 3 — Supplementary Information 3. [file 41598_2023_28754_MOESM3_ESM.zip › Heat map/N11.png]

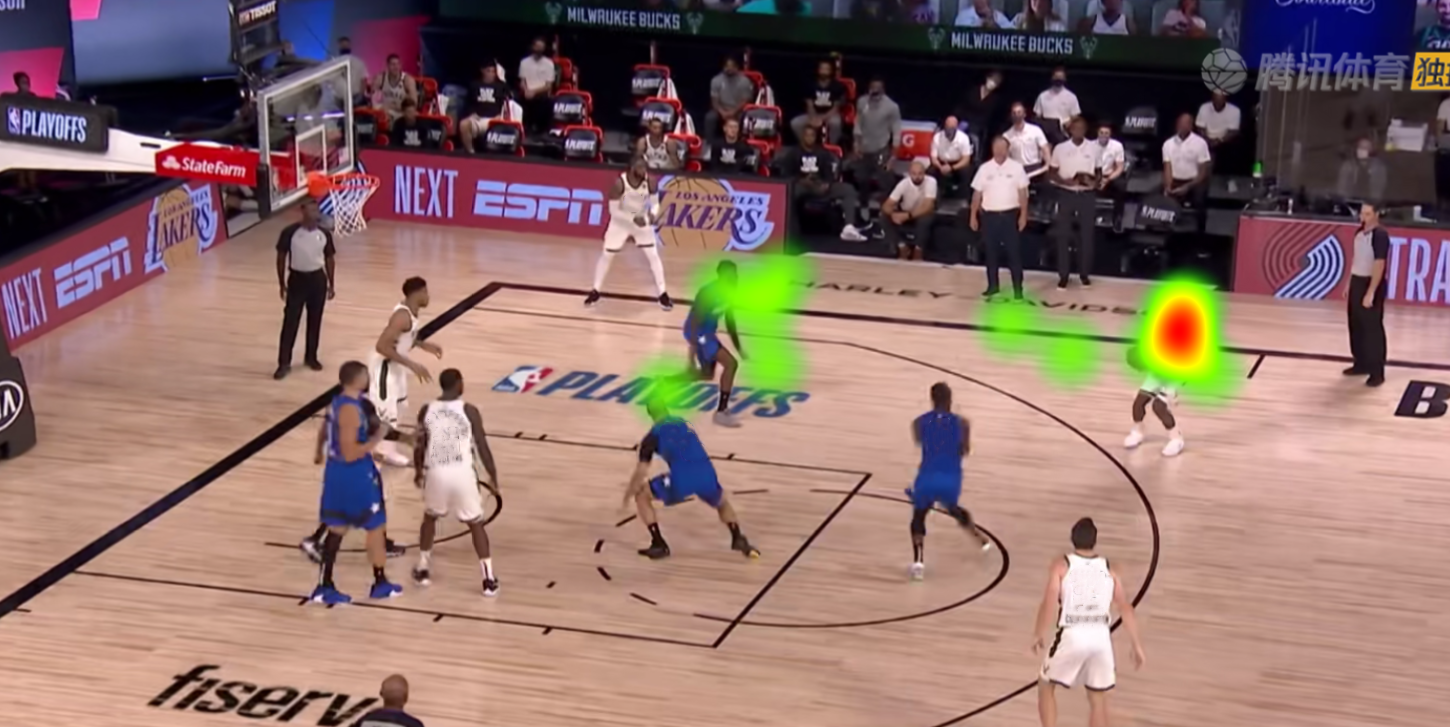

Supplement: Supplementary file 3 — Supplementary Information 3. [file 41598_2023_28754_MOESM3_ESM.zip › Heat map/N12.png]

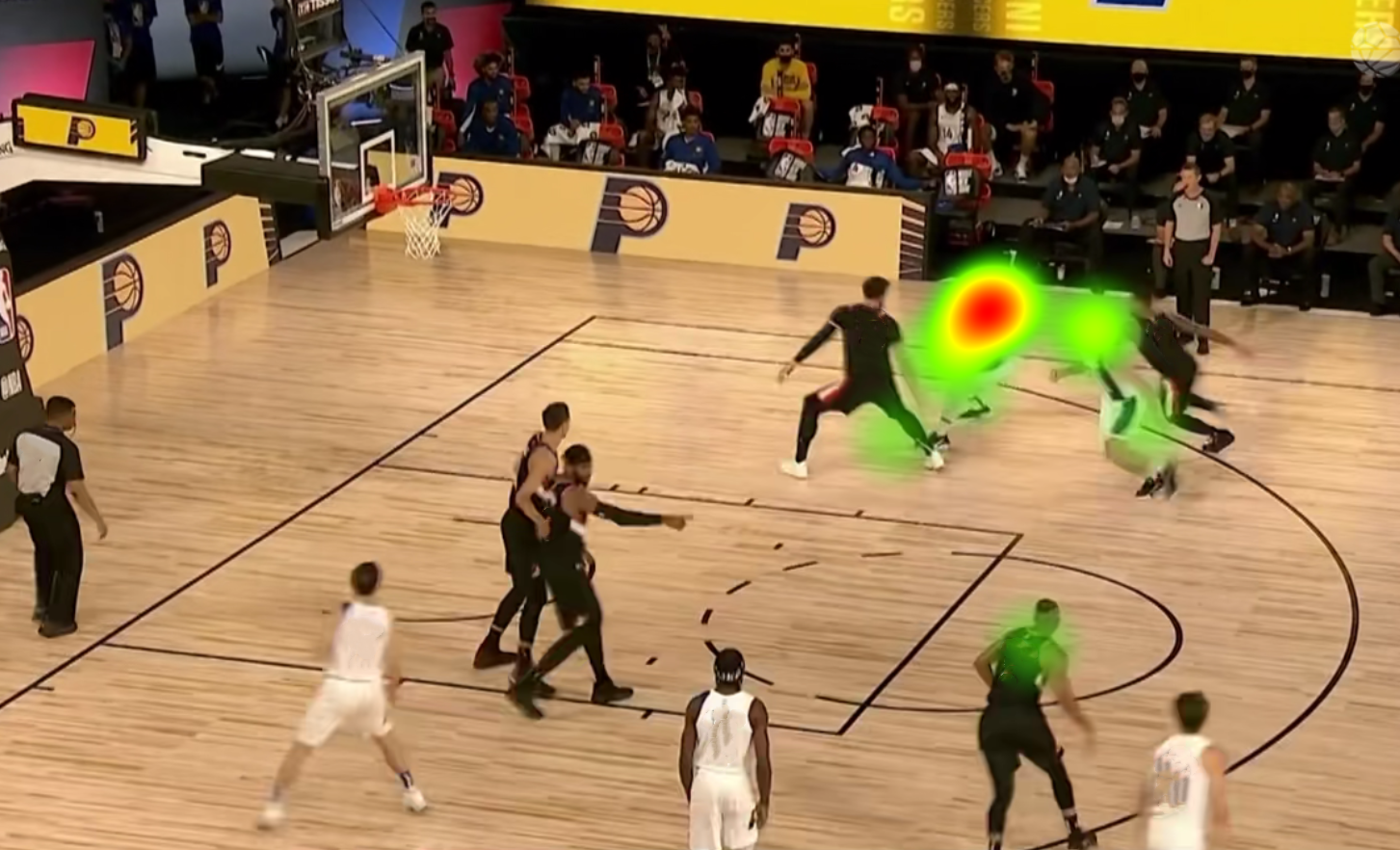

Supplement: Supplementary file 3 — Supplementary Information 3. [file 41598_2023_28754_MOESM3_ESM.zip › Heat map/N13.png]

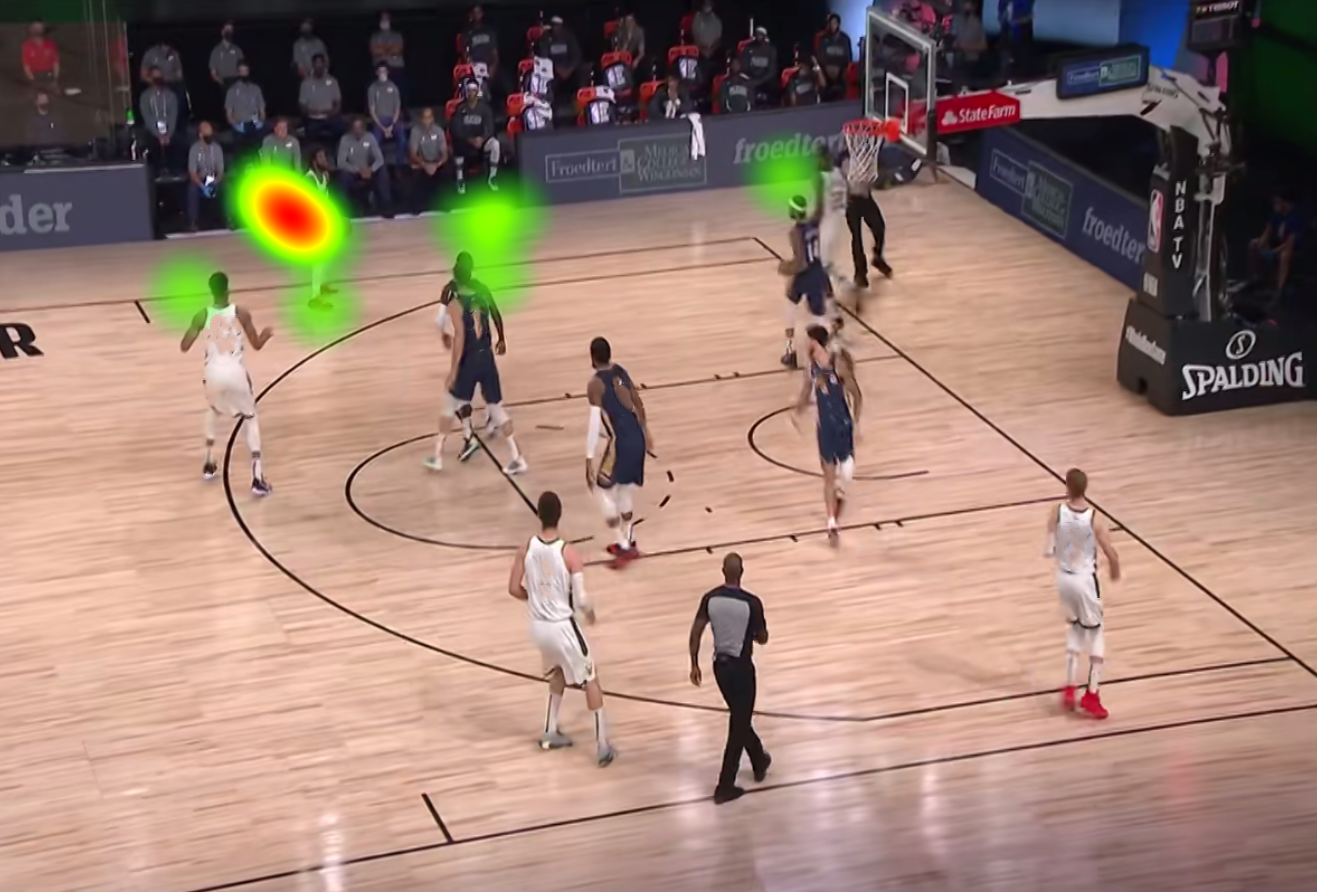

Supplement: Supplementary file 3 — Supplementary Information 3. [file 41598_2023_28754_MOESM3_ESM.zip › Heat map/N14.png]

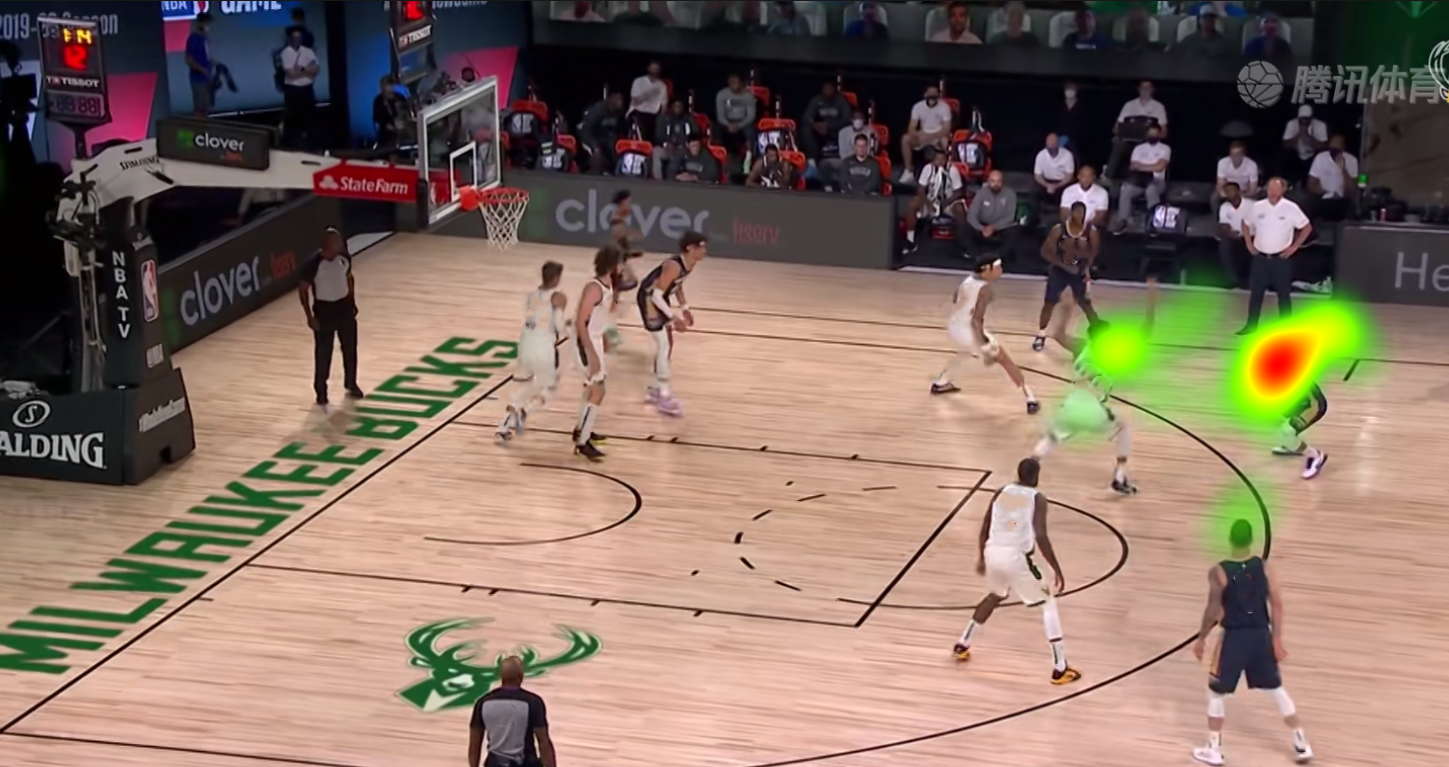

Supplement: Supplementary file 3 — Supplementary Information 3. [file 41598_2023_28754_MOESM3_ESM.zip › Heat map/N15.png]

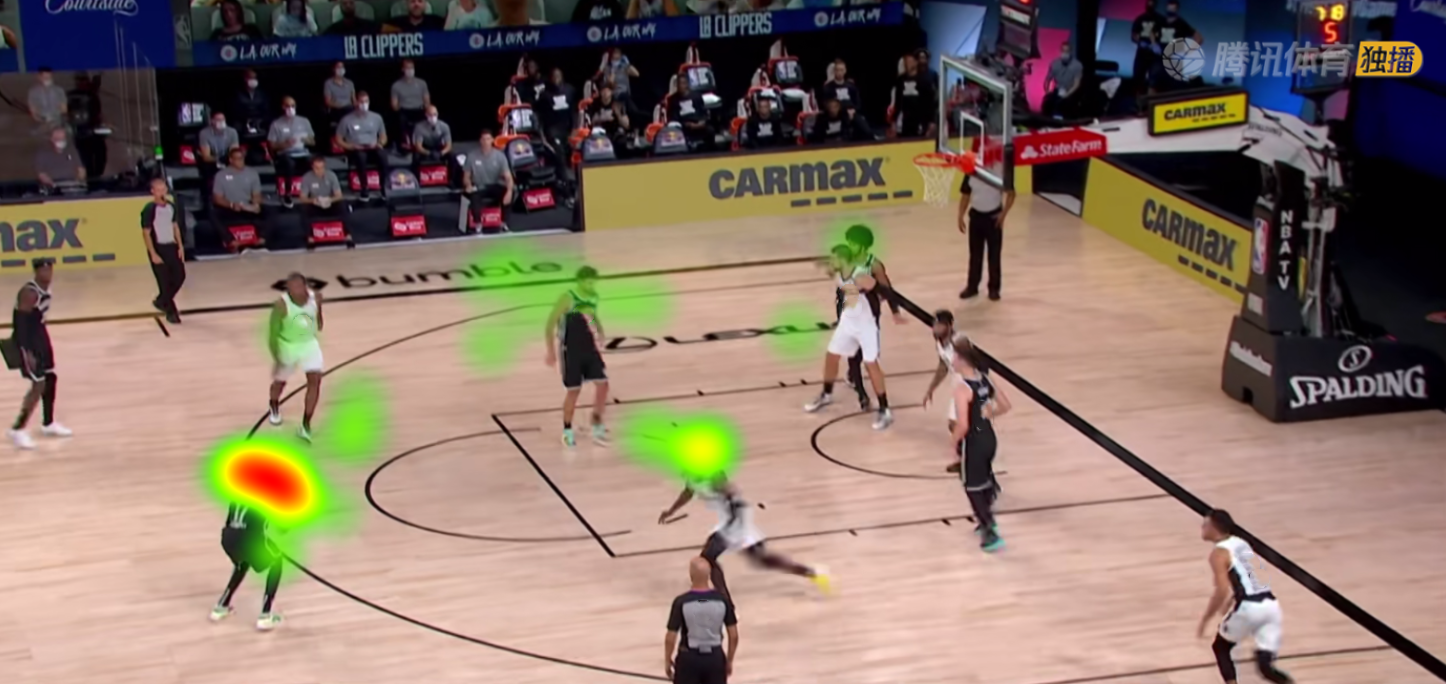

Supplement: Supplementary file 3 — Supplementary Information 3. [file 41598_2023_28754_MOESM3_ESM.zip › Heat map/N16.png]

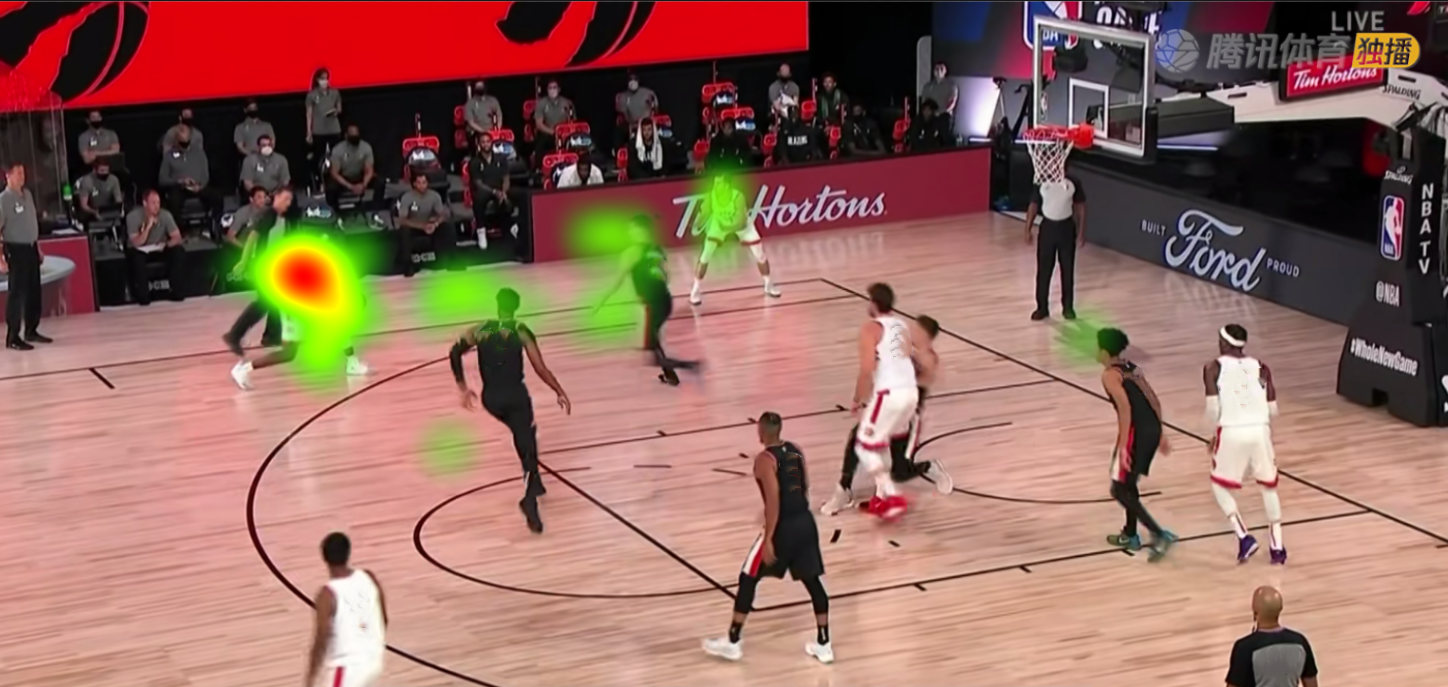

Supplement: Supplementary file 3 — Supplementary Information 3. [file 41598_2023_28754_MOESM3_ESM.zip › Heat map/N17.png]

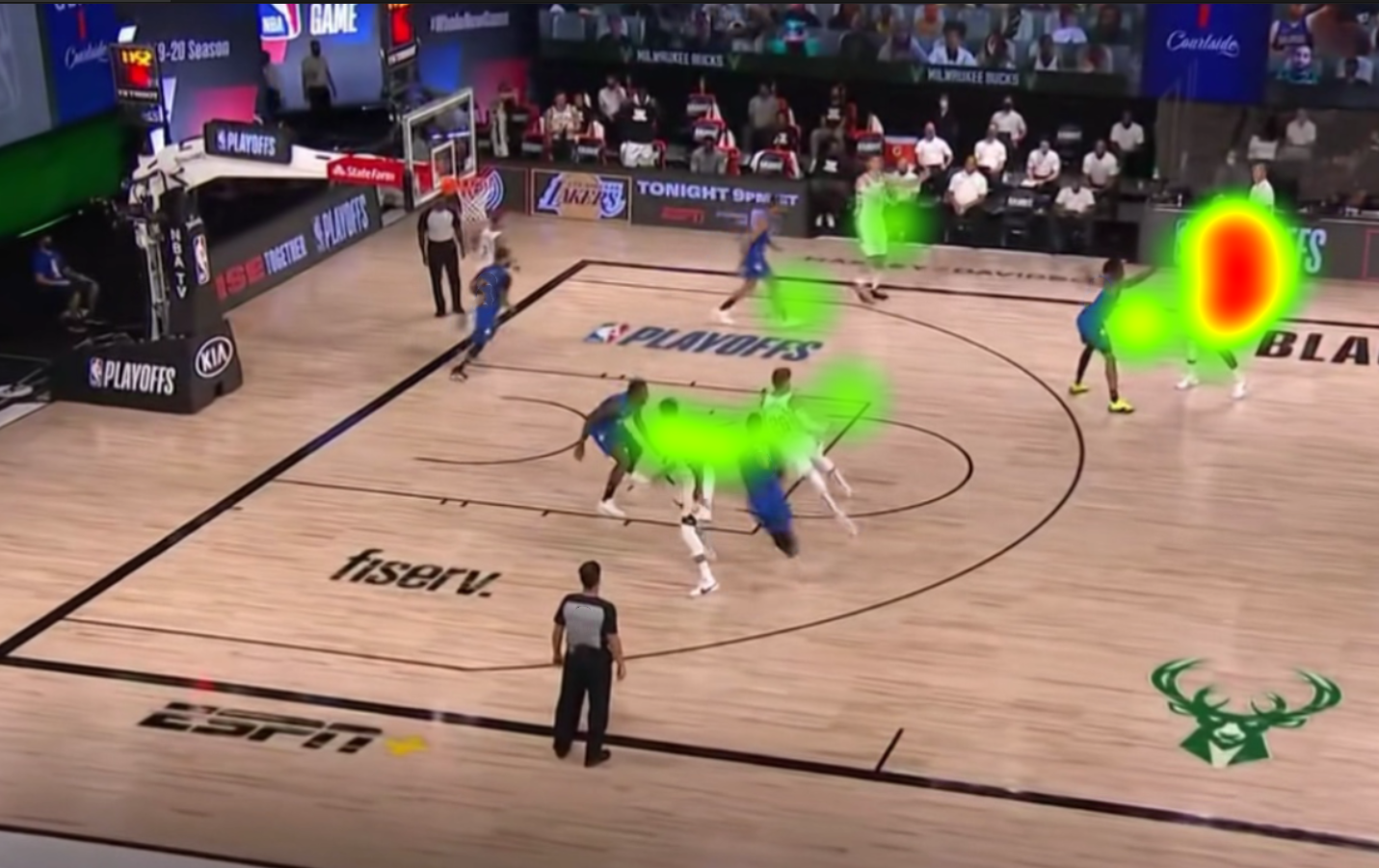

Supplement: Supplementary file 3 — Supplementary Information 3. [file 41598_2023_28754_MOESM3_ESM.zip › Heat map/N18.png]

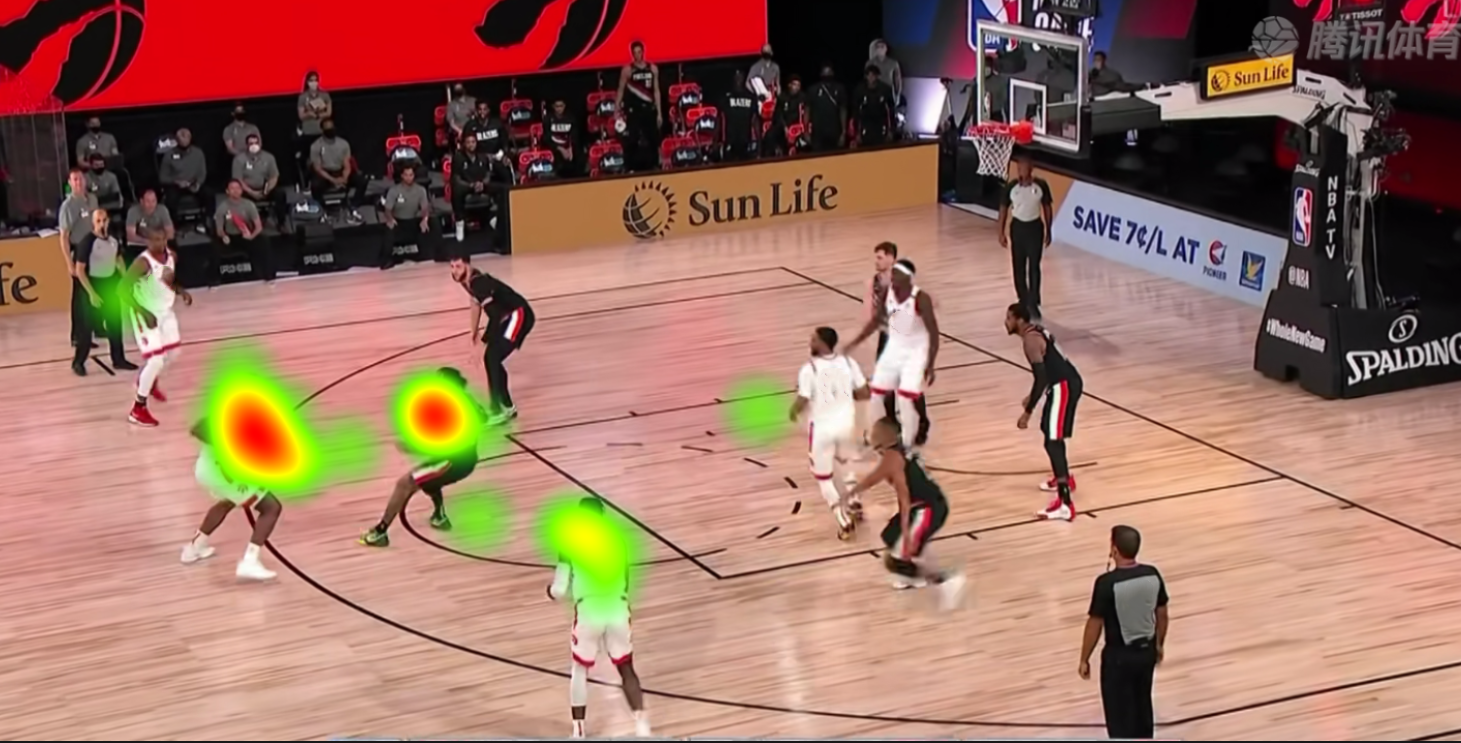

Supplement: Supplementary file 3 — Supplementary Information 3. [file 41598_2023_28754_MOESM3_ESM.zip › Heat map/N19.png]

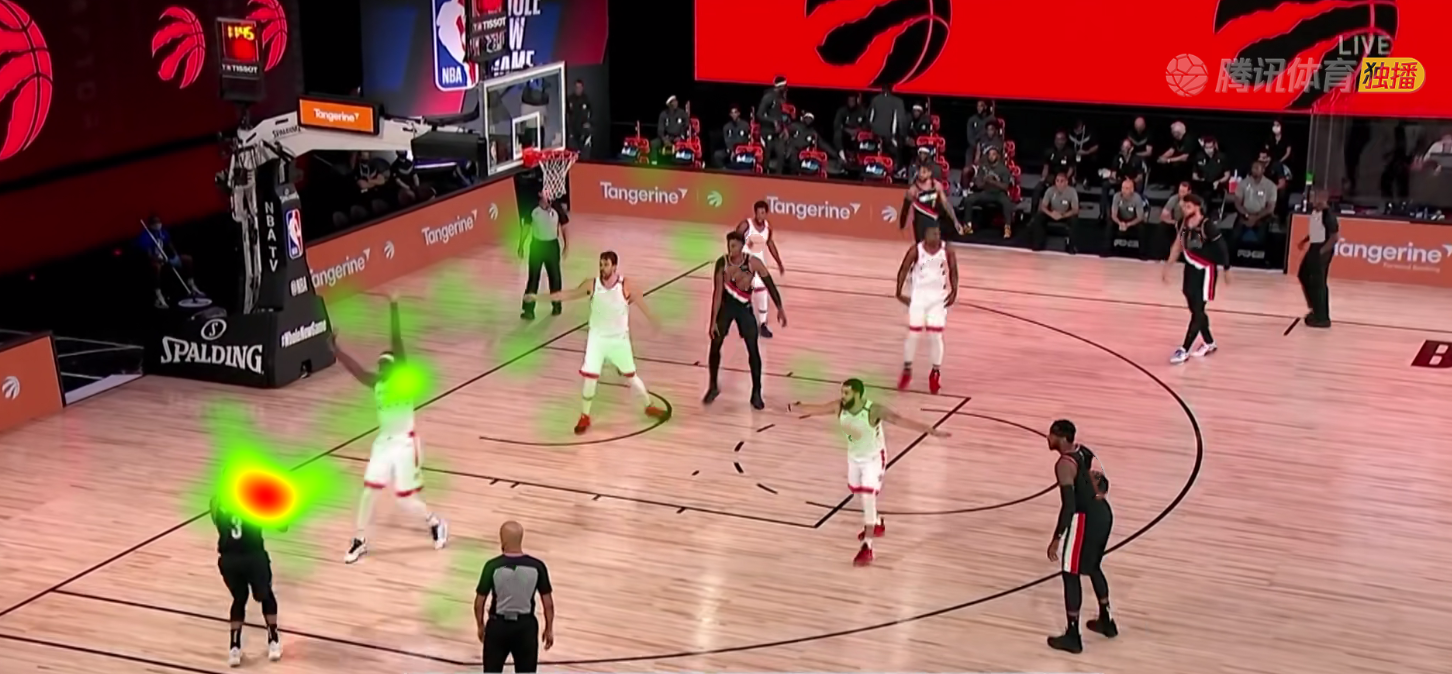

Supplement: Supplementary file 3 — Supplementary Information 3. [file 41598_2023_28754_MOESM3_ESM.zip › Heat map/N2.png]

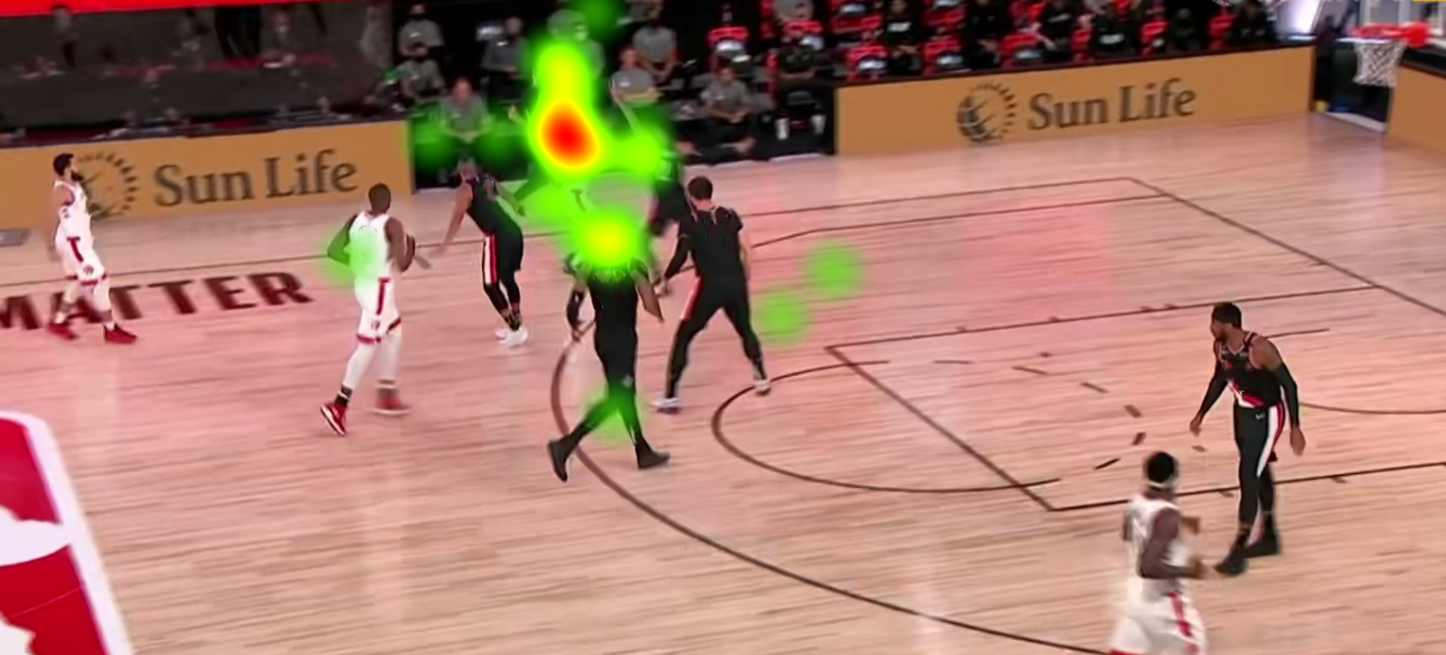

Supplement: Supplementary file 3 — Supplementary Information 3. [file 41598_2023_28754_MOESM3_ESM.zip › Heat map/N20.png]

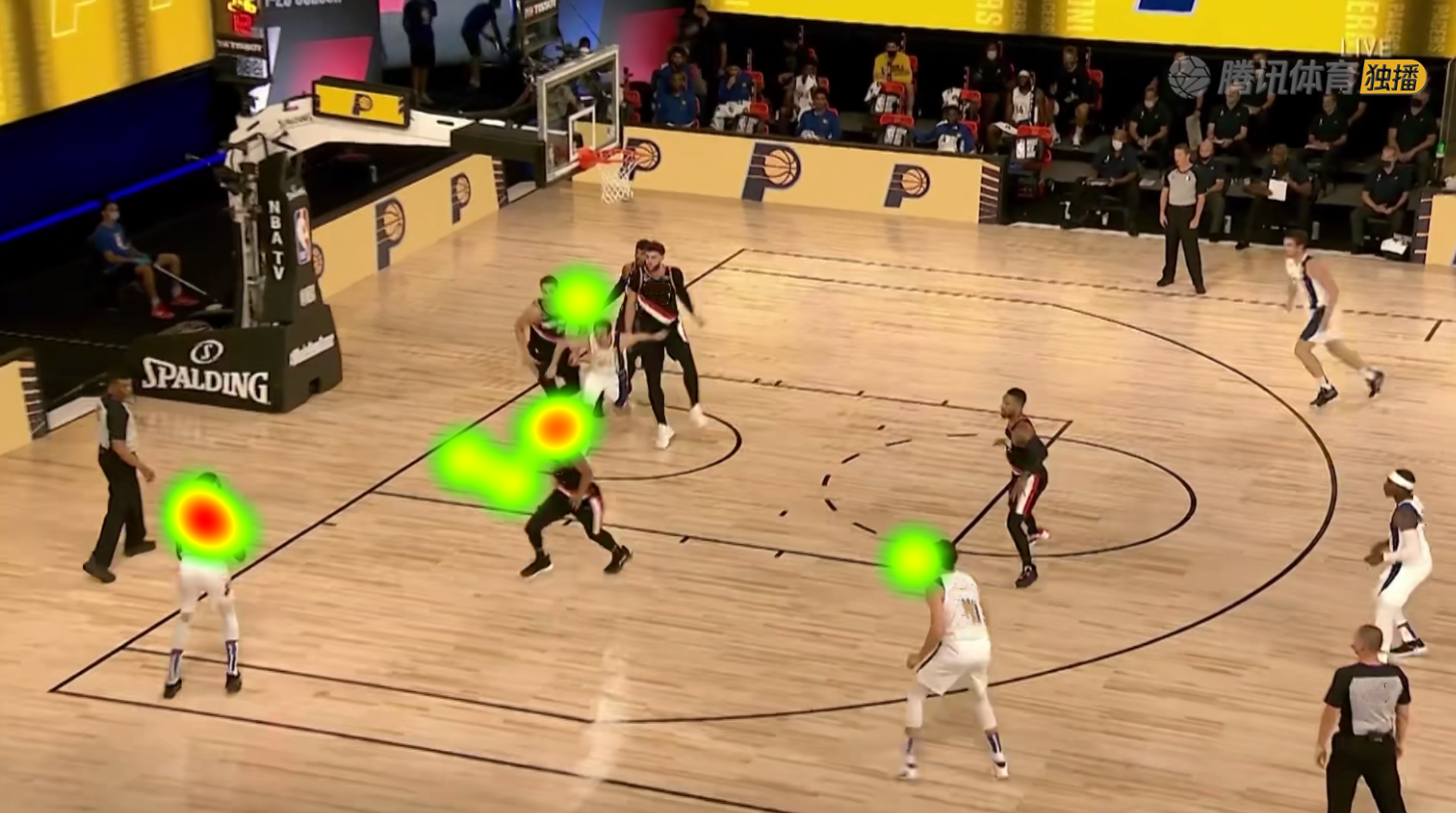

Supplement: Supplementary file 3 — Supplementary Information 3. [file 41598_2023_28754_MOESM3_ESM.zip › Heat map/N21.png]

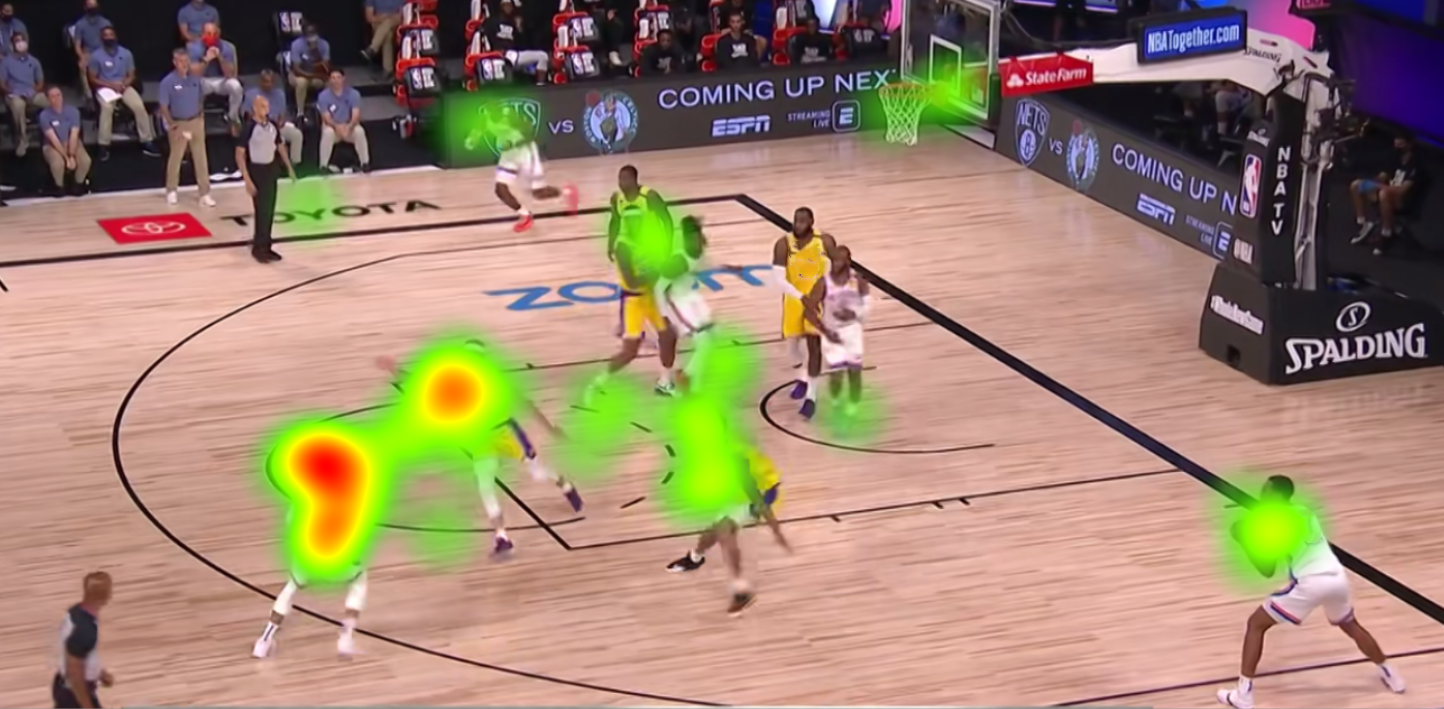

Supplement: Supplementary file 3 — Supplementary Information 3. [file 41598_2023_28754_MOESM3_ESM.zip › Heat map/N3.png]

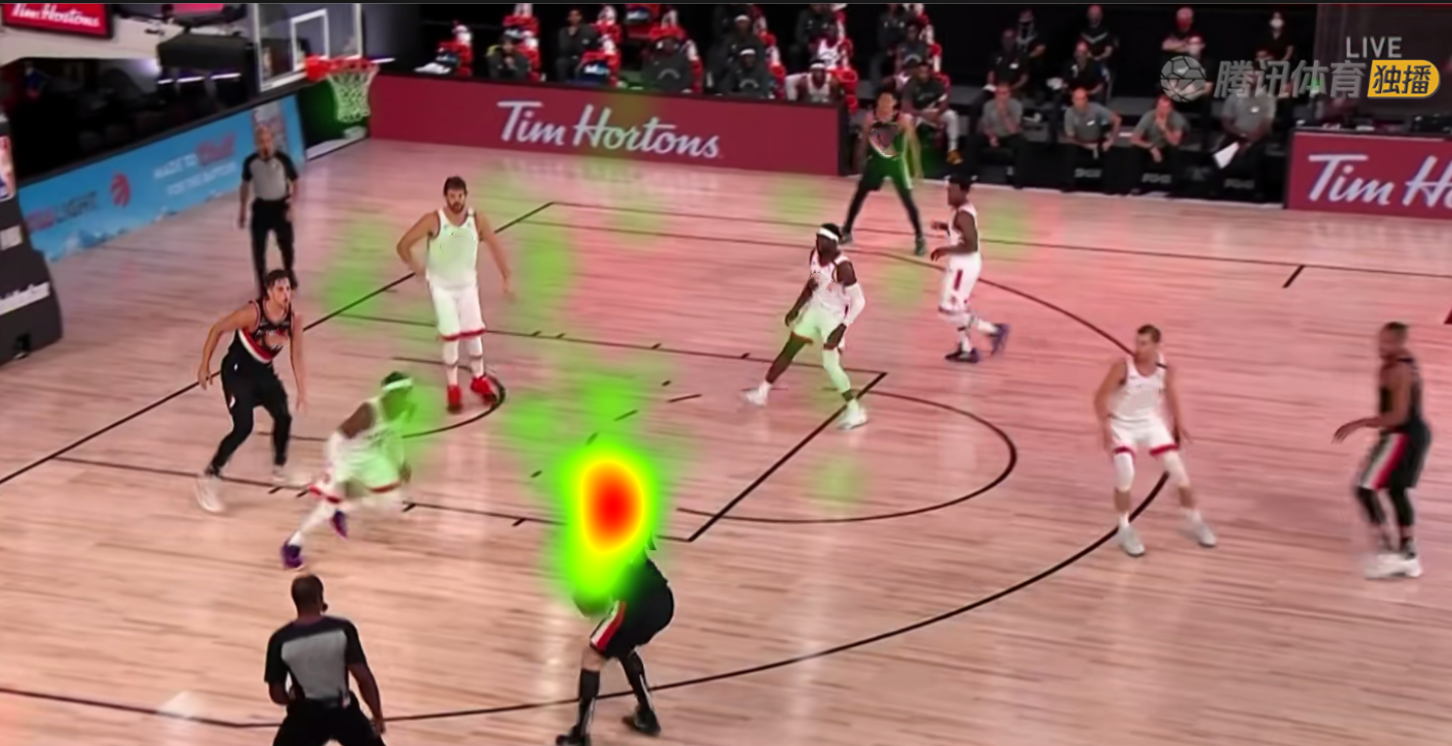

Supplement: Supplementary file 3 — Supplementary Information 3. [file 41598_2023_28754_MOESM3_ESM.zip › Heat map/N4.png]

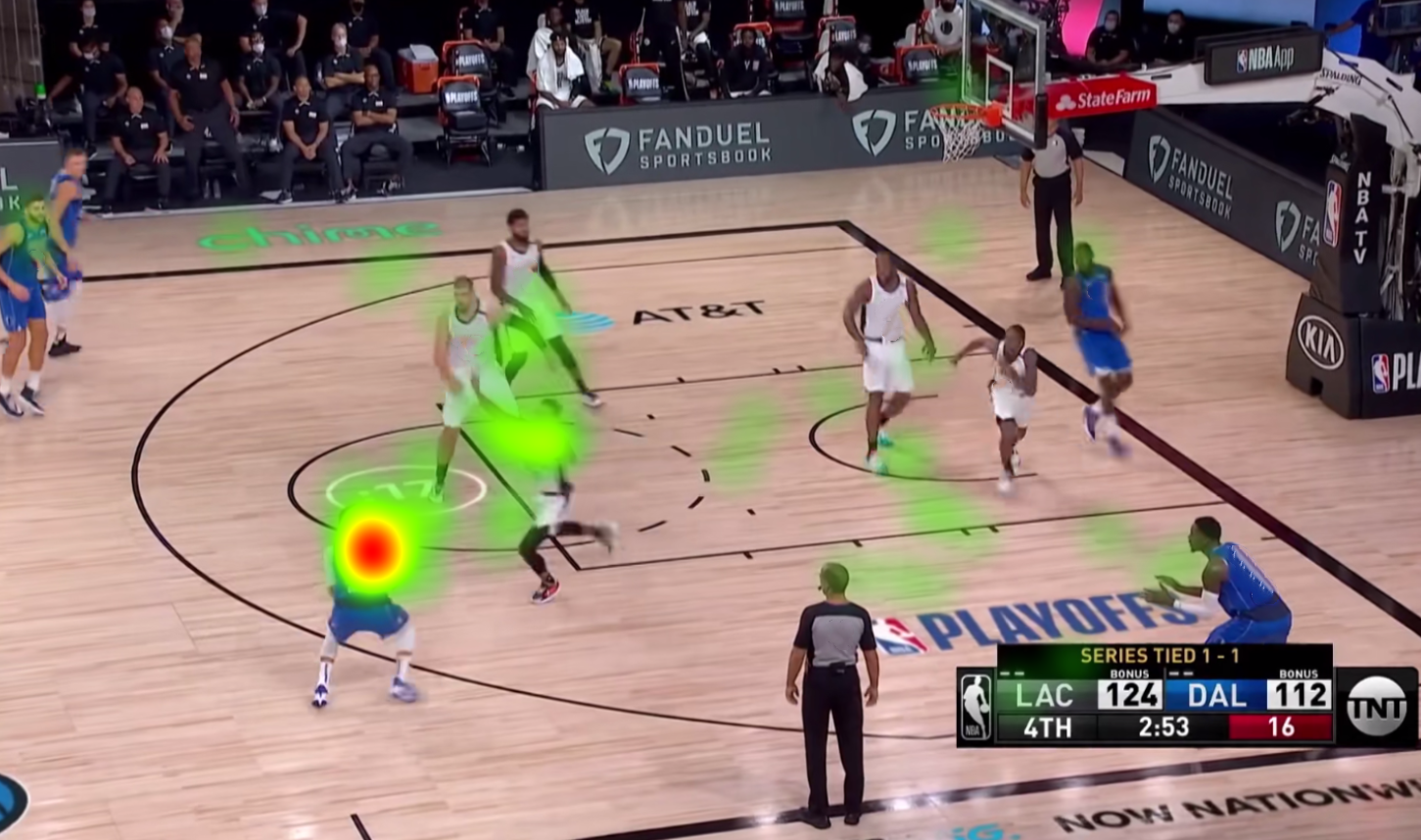

Supplement: Supplementary file 3 — Supplementary Information 3. [file 41598_2023_28754_MOESM3_ESM.zip › Heat map/N5.png]

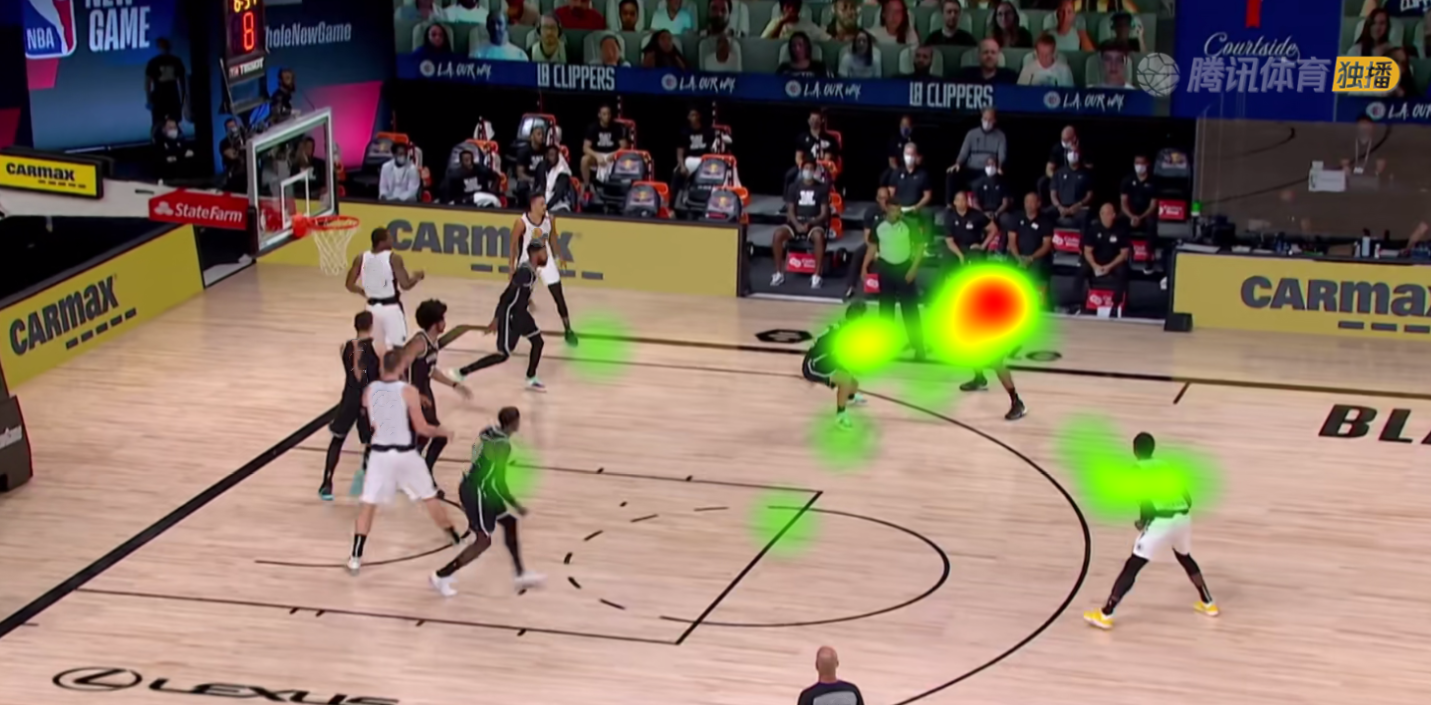

Supplement: Supplementary file 3 — Supplementary Information 3. [file 41598_2023_28754_MOESM3_ESM.zip › Heat map/N6.png]

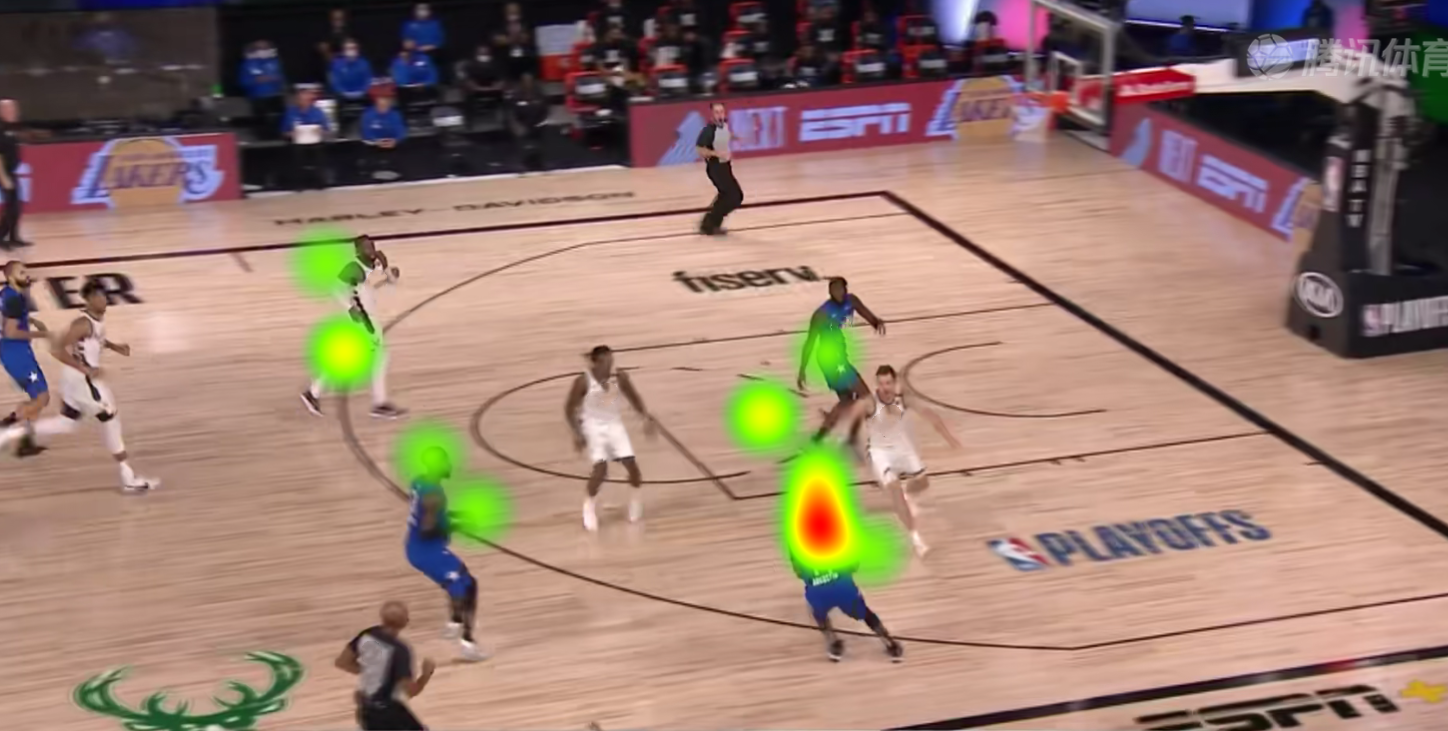

Supplement: Supplementary file 3 — Supplementary Information 3. [file 41598_2023_28754_MOESM3_ESM.zip › Heat map/N7.png]

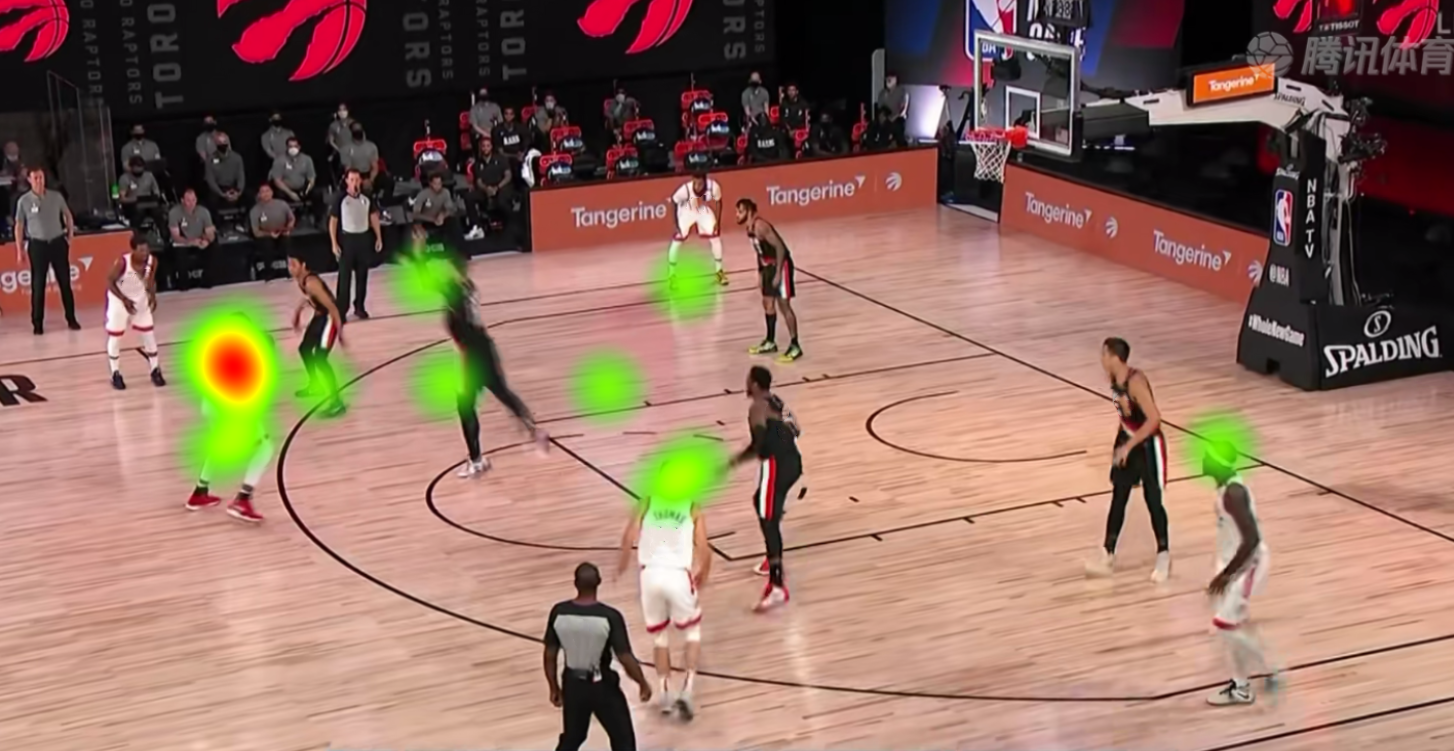

Supplement: Supplementary file 3 — Supplementary Information 3. [file 41598_2023_28754_MOESM3_ESM.zip › Heat map/N8.png]

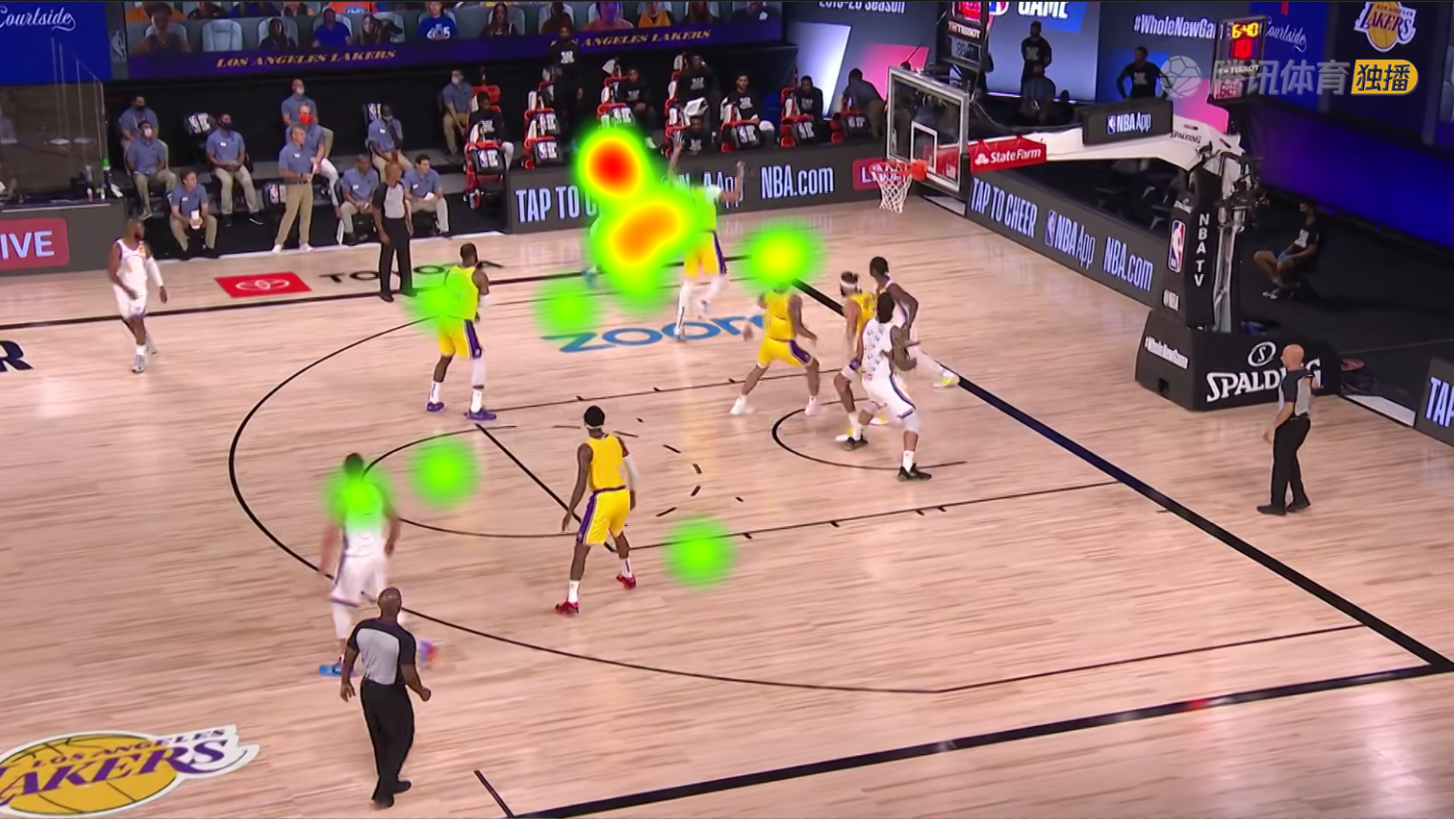

Supplement: Supplementary file 3 — Supplementary Information 3. [file 41598_2023_28754_MOESM3_ESM.zip › Heat map/N9.png]

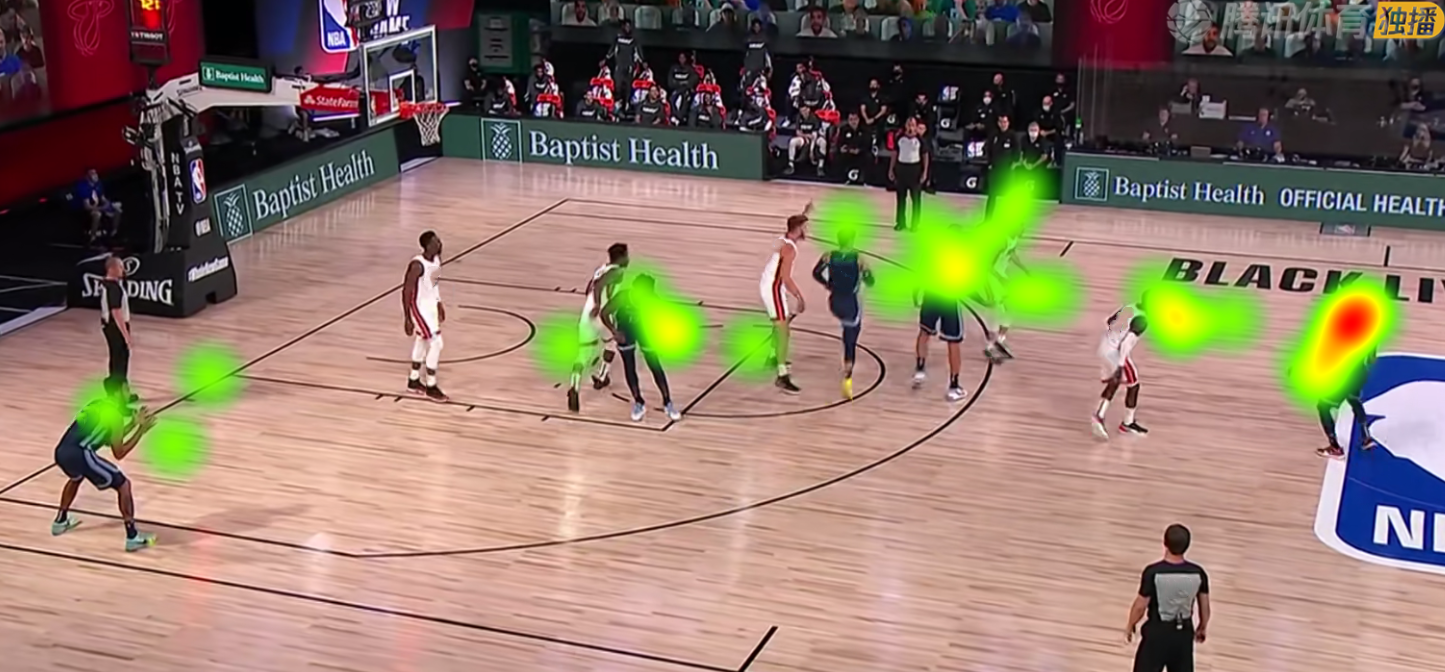

Supplement: Supplementary file 3 — Supplementary Information 3. [file 41598_2023_28754_MOESM3_ESM.zip › Heat map/Novice 1.png]
